# Adult/child ratio and group size in early childhood education or care to promote the development of children aged 0–5 years: A systematic review

**DOI:** 10.1002/cl2.1239

**Published:** 2022-05-04

**Authors:** Nina T. Dalgaard, Anja Bondebjerg, Rasmus Klokker, Bjørn C. A. Viinholt, Jens Dietrichson

**Affiliations:** ^1^ VIVE—The Danish Centre for Social Science Research Copenhagen Denmark

## Abstract

**Background:**

Worldwide, a large number of infants, toddlers, and preschoolers are enroled in formal non‐parental early childhood education or care (ECEC). Theoretically, lower adult/child ratios (fewer children per adult) and smaller group sizes are hypothesised to be associated with positive child outcomes in ECEC. A lower adult/child ratio and a smaller group size may increase both the extent and quality of adult/child interactions during the day.

**Objectives:**

The objective of this review is to synthesise data from studies to assess the impact of adult/child ratio and group size in ECEC on measures of process characteristics of quality of care and on child outcomes.

**Search Methods:**

Relevant studies were identified through electronic searches of bibliographic databases, governmental and grey literature repositories, Internet search engines, hand search of specific targeted journals, citation tracking and contact to experts. The primary searches were carried out up to September 2020. Additional searches were carried out in February 2022.

**Selection Criteria:**

The intervention was changes to adult/child ratio and group size in ECEC with children aged 0–5 years old. All study designs that used a well‐defined control group were eligible for inclusion.

**Data Collection and Analysis:**

The total number of potential relevant studies constituted 14,060 hits. A total of 31 studies met the inclusion criteria and were critically appraised by the review authors. The 31 studies analysed 26 different populations. Only 12 studies analysing 8 different populations (*N* = 4300) could be used in the data synthesis. Included studies were published between 1968 and 2019, and the average publication year was 1992. We used random‐effects meta‐analysis, applying both robust‐variance estimation and restricted maximum likelihood procedures to synthesise effect sizes. We conducted separate analyses for process quality measures and language and literacy measures.

**Main Results:**

The meta‐analysis using measures of process quality as the outcome included 84 effect sizes, 5 studies, and 6256 observations. The weighted average effect size was positive but not statistically significant (effect size [ES] = 0.10, 95% confidence interval [CI] = [−0.07, 0.27]) using robust‐variance estimation. The adjusted degrees of freedom were below 4 (*df* = 1.5), meaning that the results were unreliable. Similarly, the low number of studies made the estimation of heterogeneity statistics difficult. The *I* ^2^ and *τ* ^2^ estimates were both 0, and the *Q*‐statistic 2.3 (*p* = 0.69). We found a similar, but statistically significant, weighted average effect size using a restricted maximum likelihood procedure (ES = 0.10, 95% CI = [0.004, 0.20]), and similar low levels of heterogeneity (*Q* = 0.7, *I* ^2^ = 0%, *τ* ^2^ =  0). The meta‐analysis of language and literacy outcomes is based on three studies exploring different changes to group size and/or adult/child ratio in ECEC. The meta‐analysis of language and literacy measures included 12 effect sizes, 3 studies, and 14,625 observations. The weighted average effect size was negative but not statistically significant (ES = −0.04, 95% CI = [−0.61, 0.53]) using the robust variance estimation procedure. The adjusted degrees of freedom were again below 4 (*df* = 1.9) and the results were unreliable. The heterogeneity statistics indicated substantial heterogeneity (*Q* = 9.3, *I* ^2^ = 78.5%, *τ* ^2^ = 0.07). The restricted maximum likelihood procedure yielded similar results (ES = −0.06, 95% CI = [−0.57, 0.46], *Q* = 6.1, *I* ^2^ = 64.3%, *τ* ^2^ = 0.03).

**Authors' Conclusions:**

The main finding of the present review is that there are surprisingly few quantitative studies exploring the effects of changes to adult/child ratio and group size in ECEC on measures of process quality and on child outcomes. The overall quality of the included studies was low, and only two randomised studies were used in the meta‐analysis. The risk of bias in the majority of included studies was high, also in studies used in the meta‐analysis. Due to the limited number of studies that could be used in the data synthesis, we were unable to explore the effects of adult/child ratio and group size separately. No study that examined the effects of changes of the adult/child‐ratio and/or group size on socio‐emotional child outcomes could be included in the meta‐analysis. No high quality study examined the effects of large changes in adult/child ratio and group size on measures of process quality, or explored effects for children younger than 2 years. We included few studies (3) in the meta‐analysis that investigated measures of language and literacy and results for these outcomes were inconclusive. In one specification, we found a small statistically significant effect on process quality, suggesting that fewer children per adult and smaller group sizes do increase the process quality in ECEC. Caution regarding the interpretation must be exerted due to the heterogeneity of the study designs, the limited number of studies, and the generally high risk of bias within the included studies. Results of the present review have implications for both research and practice. First, findings from the present review tentatively support the theoretical hypothesis that lower adult/child ratios (fewer children per adult) and smaller group sizes beneficially influence process quality in ECEC. This hypothesis is reflected in the existence of standards and regulation on the minimum requirements regarding adult/child ratios and maximum group size in ECEC. However, the research literature to date provides little guidance on what the appropriate adult/child ratios and group sizes are. Second, findings from the present review may be seen as a testimony to the urgent need for more contemporary high‐quality research exploring the effects of changes in adult/child ratio and group size in ECEC on measures of process quality and child developmental and socio‐emotional outcomes.

## 1 PLAIN LANGUAGE SUMMARY

### 1.1 Adult‐to‐child ratios and group sizes in early childhood education and care (ECEC) need more high‐quality research

There are surprisingly few high‐quality studies exploring the effects of adult/child ratio and group size in ECEC using a methodologically suitable study design.

Based on the available evidence, it is not possible to draw any definitive conclusions regarding the impact of adult/child ratio and group size on children in ECEC. However, the results of a meta‐analysis tentatively suggest that fewer children per adult and smaller group sizes do increase process quality—defined as more positive adult/child and child/child interactions, less coercive and controlling adult interference, and less aggressive and more prosocial child behaviour.

### 1.2 What is this review about?

Every day around the globe, a large number of children aged 0–5 years old spend a majority of their waking hours in ECEC. Theoretically, structural features of ECEC settings, such as lower adult/child ratios (fewer children per adult) and smaller group sizes, are proposed to be associated with increased process quality.

In this review, increased process quality is defined as an increase in nurturing and stimulating adult/child interactions, meaning less detached and controlling caregiver behaviours, fewer conflicts and aggressive child behaviour, more prosocial child behaviour and fewer children who are aimlessly wandering around without being meaningfully engaged in activities.

**What is the aim of this review?**

This systematic review examines the effects of reducing adult/child ratios and group sizes on process quality and on individual children's psychosocial adjustment, development and well‐being in ECEC for children aged 0‐5 years old. The review analyses evidence from 12 studies, two of which were randomised control trials, representing eight different populations.

### 1.3 What studies are included?

Very few high‐quality quantitative studies have examined the effects of different adult/child ratios and group sizes in ECEC. High‐quality studies did not cover all age groups and no high‐quality studies have explored the effects of the adult/child ratio on children's socio‐emotional adjustment and well‐being.

Similarly, no high‐quality studies have explored the long‐term effects of adult/child ratios and group sizes in ECEC.

In total, 31 studies met the inclusion criteria, for example, they were quantitative studies using a well‐defined control group. The studies analysed 26 different populations. Only 12 studies (analysing eight different populations) could be used in the data synthesis. The included studies were from Australia (1), Denmark (1), England (1), Italy (1), Korea (1), New Zealand (2), Portugal (1), Sweden (2), The Netherlands (1) and the USA (20).

### 1.4 What are the main findings of this review?

The main finding of the review is that there are surprisingly few high‐quality studies exploring this study question using a methodologically suitable study design. Furthermore, the existing studies on the topic are on average almost 30 years old, and there is not a single high‐quality study exploring the effects of different adult/child ratios and group sizes for children younger than 2 years old.

Similarly, no high‐quality studies explored the long‐term effects of adult/child ratio and group size in ECEC.

Results of the meta‐analysis on language and learning outcomes are inconclusive, while the results of the meta‐analysis analysing process quality outcomes suggest an effect in the expected direction.

### 1.5 What do the findings of this review mean?

The review should be interpreted cautiously due to the low quality of the evidence. However, results tentatively support the theoretical impact of two structural features—adult/child ratio and group size—on process quality, which is reflected in legislation and quality standards imposing minimum requirements on the adult/child ratio and a maximum group size in ECEC settings.

Findings from the review serve as a testimony to the urgent need for more contemporary research on the effects of adult/child ratio and group size in ECEC. Reducing the adult/child ratio and group size in ECEC is costly, and we do not know if they lead to improvement, as the research literature to this day provides little guidance on optimal adult/child ratio and group size in ECEC.

### 1.6 How up‐to‐date is this review?

The primary searches were carried out until September 2020. Additional searches were performed in February 2022.

## 2 BACKGROUND

### 2.1 The problem, condition or issue

Worldwide, a large number of infants, toddlers, and preschoolers are enroled in formal non‐parental early childhood education or care (ECEC). Formal ECEC is defined as professional early childcare or education settings with paid caretakers or teachers as opposed to more informal arrangements such as private babysitters or caretakers consisting of members of the child's extended family. On average across OECD countries, around 33% of children aged 0‐2 years old are enroled in ECEC, but this ranges from lower than 1% in Turkey to as high as roughly 60% in Belgium and Denmark. For children aged 3–5 years old, the enrolment rates are even higher with an average of 87.2% across the OECD.^1^

Average hours in ECEC also differ across countries. In most OECD countries, children (0–2‐year‐olds) attend ECEC for an average of 25 and 35 h during a usual week, with the OECD average just under 30 h per week (see footnote 1). An overall average is not available for 3–5‐year‐olds in the OECD countries, but in Denmark children aged 3–5 years old spend an average of 7.5 h each day in kindergarten.^2^ In the developing countries, formal childcare is also increasing. In the past 20 years, at least 13 developing countries have instituted compulsory preschool or preprimary programmes (Engle et al., 2011), and according to The World Bank, roughly half of all children in the relevant age range around the globe were enroled in preschool in 2017.^3^ Thus, with a large number of children spending a substantial number of hours every day in non‐parental care, it becomes important to examine the impact of the quality of care on the development and well‐being of children.

Quality of care in ECEC may be defined by both structural and process characteristics (Vermeer et al., 2016). Structural characteristics include the adult/child ratio, group size, the formal educational level of staff, years of working experience and in‐service professional development of the caretakers/teachers, and the physical child care facilities (Slot et al., 2015). Process characteristics include the caretakers' sensitivity and the quality of the child‐caretaker interactions during the day (de Schipper et al., 2006). The two aspects of quality of care are associated with each other (NICHD, 1996). Both structural and process characteristics are associated with positive child outcomes (Auger et al., 2014; Burchinal et al., 1996; Burchinal et al., 2002; Howes et al., 1992; Phillips et al., 2000). However, some studies have also failed to find a positive association between a lower adult/child ratio (fewer children per adult) and positive child outcomes (Clarke‐Stewart et al., 1994; Dunn, 1993; Mashburn et al., 2008) or have reported mixed results (Howes, 1997).

Structural characteristics are readily observable and easier to regulate than process characteristics. However, the specific impact of different structural characteristics on both process characteristics and on child outcomes has yet to be rigorously examined in a systematic review, which is where the present review contributes. In the present review, we examined the effect of two central structural characteristics: adult/child ratio and group size on both process characteristics and on child outcomes.

### 2.2 Description of the intervention

In this systematic review, we examined the impact of adult/child ratio and group size on child development and well‐being in formal non‐parental ECEC settings. Thus, the intervention was defined as any change to adult/child ratio and/or group size which had been reliably measured within an eligible setting.

Interventions may change the adult‐child ratio, the group size, or both simultaneously. That is, to increase the group size while keeping the ratio constant, the number of children needs to increase by exactly the same proportion as the number of adults (e.g., by doubling both the number of children and adults). If an intervention only increases the number of children, the adult/child ratio and the group size increases. If the number of adults increases, the adult‐child ratio decreases while the group size is constant.

As stated in the protocol (Dalgaard et al., 2020), we aimed to be able to distinguish between interventions that change the adult‐child ratio, the group size, or both the ratio and the group size. However, this was not possible due to the low number of included studies, which could be used in the meta‐analysis.

### 2.3 How the intervention might work

Theoretically, lower adult/child ratios (fewer children per adult) and smaller group sizes are hypothesised to improve child outcomes. A lower adult/child ratio and a smaller group size are proposed to increase both the extent and quality of adult‐child interactions during the day. The younger the children are, the more their development and well‐being are proposed to be dependent on adequate, nurturing and stimulating adult‐child interactions. The extent and quality of adult‐child interactions are proposed by some scholars to be the single most important determinants for the child's development and well‐being within ECEC settings (de Schipper et al., 2006; Christoffersen et al., 2014; Karoly, 1998; Lamb, 1998; Munton et al., 2002; Vandell & Wolfe, 2000).

Studies suggest that when the adult/child ratio and group sizes are decreased, the number of interactions between each child and an adult increases and the nature of the exchanges becomes more stimulating and nurturing for the child. Thus, caregivers with fewer children in their care have been found to be more sensitive, responsive, warm, nurturing, and encouraging towards the children. Furthermore, a lower adult/child ratio has been found to be associated with adults exhibiting more positive and less negative affect, and with adults who provide more varied and developmentally appropriate activities for the children. Previous studies further suggest that when fewer adults are in charge of a larger group of children, the caregivers become more focussed on managing and controlling the children's behaviour. This means that the adults will give more commands and corrections, exert more negative control, and spend less time engaged in reciprocal conversations or playful interactions with the children. With higher ratios (more children per adult)) and larger group sizes, the adults will be more likely to ignore or overhear children's questions and they will spend less time engaged in positive affirmation. Furthermore, early studies suggest that with higher ratios and group sizes, children will have more conflicts during free play situations and thus the adults may need to spend more time on acute problem solving (Christoffersen et al., 2014; Dawe, 1934; Gevers et al., 2005; Ghazvini & Mullis, 2002; Howes, 1983, 1997; Howes & Rubenstein, 1985; Howes et al., 1995; NICHD ECCRN, 1996, 2000; Palmeérus, & Hägglund, 1991; Phillipsen et al., 1997; Roudinesco, & Appel, 1950; Sjølund, 1969; Stallings & Porter, 1980; Volling & Feagans, 1995; Williams, & Mattson, 1942).

Furthermore, previous studies have also found lower adult child/ratio and group size to be associated with positive child outcomes such as decreased levels of anxiety, aggressive behaviour and distress, greater social competence, and better receptive and expressive language skills (Burchinal et al., 1996; Vernon‐Feagans et al., 1996; Volling & Feagans, 1995). Theoretically, this may be explained by both the quality and frequency of the adult/child interactions. However, some scholars also suggest that a smaller group size, regardless of the adult/child ratio, may be beneficial to the group dynamic and may decrease the children's stress levels (Christoffersen et al., 2014).

However, findings regarding the impact of adult/child ratio and group size are far from unequivocal, as a number of observational studies have failed to find significant positive associations between adult/child ratio and group size and the expected process quality and child outcomes (Barros & Anguiar, 2010; Fukkink et al., 2013; Pessanha et al., 2007; Pianta et al., 2005; Vermeer et al., 2008). An example of a study which does not support the association between group size and adult/child ratio and positive process quality outcomes is Slot et al. (2015). In this study, based on a national Dutch cohort study of preschool education and care provisions, child‐to‐teacher ratio and group size did not explain variance in emotional or educational process quality between ECEC classrooms. Similarly, Blau (2000) found a small and statistically insignificant association between group size and child care quality and only a small positive association between adult/child ratio and child care quality in a study based on data from a random sample of day care centres in four different states in the United States.

In summary, despite some previous contradictory findings, the adult/child ratio and group size are hypothesised to affect the process characteristics of quality of care, meaning that a reduced adult/child ratio and group size are associated with an increase in positive child‐caretaker interaction and in caretaker sensitivity, responsiveness, warmth, nurture, and encouragement towards the children, and with more positive and less negative affect. Furthermore, a reduced adult/child ratio and group size are hypothesised to be associated with positive cognitive, behavioural, and socio‐emotional child outcomes.

### 2.4 Why it is important to do this review

To our knowledge, no systematic review of the effects of adult/child ratio and group size in ECEC on the process quality and on child outcomes has previously been carried out.

Perlman et al. (2017) conducted a systematic review and meta‐analysis of adult/child ratio in ECEC settings on child outcomes. The purpose of this systematic review was to evaluate the association between adult/child ratios and children's outcomes. Searches revealed 29 relevant studies, with only three studies eligible for inclusion in the meta‐analysis. These three studies focused exclusively on associations between child/staff ratios and children's receptive language, thus not allowing for broader conclusions regarding child outcomes in other areas.

While the review by Perlman et al. provides important insights, the scope of the present review was broader as we sought to examine the causal effects of both adult/child ratio and group size and included process quality measures as outcomes. Furthermore, while the review by Perlman et al. only examined children aged between 30 and 72 months, we included children within a broader age range. Finally, the present review included an extensive risk of bias assessment.

Whereas process characteristics of quality of care are difficult to measure and regulate, the structural characteristics are readily observable and easier to regulate. However, reducing the adult/child ratio and group sizes is costly. Therefore, it is important to determine the overall and relative efficacy of such reductions in facilitating optimal development and well‐being in children attending ECEC.

## 3 OBJECTIVES

The objective of the present review was to synthesise data from studies to assess the impact of adult/child ratio and group size in ECEC on measures of process characteristics of quality of care and on child outcomes.

## 4 METHODS

### 4.1 Criteria for considering studies for this review

#### 4.1.1 Types of studies

To summarise what is known about the causal effects of adult/child ratio and group size on process quality characteristics and child outcomes in ECEC settings with children aged 0–5 years old, quantitative studies with a well‐defined control group were eligible. The study designs eligible for inclusion were:

1. Controlled trials
  Randomised controlled trials (RCTs)
  Quasi‐randomised controlled trial designs (QRCTs). Here participants are allocated by means, which are not expected to influence outcomes, for example, alternate allocation, participant's birth data, case number, or alphabetic order.
2. Quasi‐experimental studies (QES). This category refers to both studies, where participants are allocated by other actions controlled by the researcher, or where allocation to the intervention and control group are not controlled by the researcher (e.g., allocation according to time differences or policy rules). This definition implies that the process, or mechanism, by which the difference in adult/child ratio or group size, between the treatment and control groups, was altered must be clearly elaborated in studies that apply a QES study design. Examples could be studies in which a state‐level policy change mandated minimum requirements for adult/child ratio or group size or a threshold indicating when daycare centres would be eligible to receive additional resources that they could spend on hiring additional caregivers. Conversely, observational studies that seek to estimate causal effects via, for example, adjustment by regression or matching typically do not include such descriptions of mechanisms or assignment procedures. While both regression adjustment and matching seek to eliminate confounding, and thereby make treatment status ‘as good as random’, these methods typically do not address how the observed differences in adult/child ratio or group size came about. As such, methods such as regression adjustment and matching would typically not be eligible for inclusion in this review.

To be included in the meta‐analysis, QRCTs and QESs must credibly demonstrate that outcome differences between intervention and control groups are the effect of the intervention and not the result of systematic baseline differences between groups. That is, selection bias should not be driving the results. This assessment is included as part of the risk of bias tool, which we elaborate on in the Risk of bias section.

To include all relevant data, we also included studies using a repeated measures experimental design in which the same caregiver and/or children were observed under different conditions within a short time span. In such a single‐group design, children and caregivers act as their own control group. As children and caregivers develop their skills over time, single‐group repeated measures designs are prone to confounding intervention effects with naturally occurring child and caregiver development (e.g., Morris & DeShon, 2002). Therefore, we paid special attention to the risk of confounding intervention effects with the natural skill development in single‐group repeated measures designs.

In accordance with the criteria stated above and the aim to study causal effects, we excluded studies reporting associations in cohort, cross‐sectional, and longitudinal study designs, if they did not include a relevant comparison group.

To minimise the risk of bias, we also excluded study designs in which only one unit was assigned to the intervention or control group. That is, there had to be at least two units in the intervention group and two units in the control group, otherwise there would be a very high risk of confounding treatment effects with ‘unit’ effects. Finally, we excluded studies using noncomparable treatment and control groups, for example, studies that compared highly selected groups, such as comparisons of at‐risk to not‐at‐risk children.

#### 4.1.2 Types of participants

This review aimed to include studies of children aged 0‐5 years old who were enroled in some form of formal non‐parental ECEC. Formal ECEC was defined as professional settings with paid caretakers or teachers. We included studies of children with special needs and children considered at risk. We excluded children living in any kind of residential care arrangements such as foster families or institutions.

#### 4.1.3 Types of interventions

We examined the impact of different adult/child ratios and group sizes on child development and well‐being in formal non‐parental ECEC settings. We defined eligible interventions as any changes in adult/child ratio and/or group size which had been reliably measured within an eligible setting.

To be eligible for inclusion, studies had to report either adult/child ratio and/or group size. In measuring these variables, we accepted studies using both direct observation and register‐based data in which the adult/child ratio was derived from information regarding the number of staff and the number of children within each ECEC facility. The reason for including studies using register‐based data is that we wanted the review to be as comprehensive as possible.

#### 4.1.4 Types of outcome measures

The objective of the review was to explore the impact of changes to adult/child ratio and group size on both process characteristics of quality of care as well as on child outcomes. The review aimed to explore both developmental child outcomes as well as child well‐being.

In the protocol, we stated that we would only extract outcomes, if they had been validated on other samples than the intervention sample (researcher observations, caregiver or parental ratings) (Dalgaard et al., 2020). However, due to the very limited number of included studies within this review, we decided to include measures, which had not been validated on other samples, if they were deemed high on face validity and provided a measure of interrater reliability. Examples of measures with a high face validity would be an observation schedule describing very concrete child and adult behaviours such as ‘crying’, ‘aimless wandering’, and ‘adult praises child’. This was the case with Russell (1990), Smith et al. (1988), and Smith & Connolly (1986), in which the authors stated that the observation schedules were designed specifically for their studies. One study, de Schipper et al. (2006), used an observation schedule which consisted of items from different previously validated scales measuring child‐caregiver interaction. Outcomes based on observation schedules were only included, when they were deemed high in face validity by two authors. Ambiguous outcomes, in which it was not possible to judge the direction of scores (e.g., is a high score beneficial?), such as ‘child plays with blocks’, were excluded.

In the five studies, which could be used in the meta‐analysis on process quality outcomes, we extracted the following outcomes in addition to the observation schedules designed for the specific studies:

Process quality:

– The Arnett Caregiver Interaction Scale (CIS) (Arnett, 1989)
– The Classroom Assessment Scoring System (Pianta et al., 2008)
– Child‐Focus Instrument (Prescott, 1975)
– Adult‐Focus Instrument (Stallings et al., 1975)

Child outcomes:

We did not include any study analysing the effects of changes to adult/child ratio and group size in ECEC on child level measures of socio‐emotional adjustment or well‐being.

In the three studies, which were used in the meta‐analysis of language and literacy outcomes, we extracted the following outcomes:

– Peabody Picture Vocabulary Test (Dunn & Dunn, 1997)
– Test of Preschool Emergent Literacy (Wilson & Lonigan, 2009)
– The Language Assessment of Children: 3–6 instrument (Bleses et al., 2010).

One study (Neuman & Kaefer, 2013) also used a vocabulary task to measure the number of curriculum‐specific words children learned throughout each unit of instruction, which was specifically designed for the study. This was also extracted for the present review

#### 4.1.5 Primary outcomes

Based on the objectives of the present review, we did not distinguish between primary and secondary outcomes.

#### 4.1.6 Secondary outcomes

##### Duration of follow‐up

We did not restrict the outcomes in terms of the duration of follow‐up but we did not include a single study with measurement at time points beyond the end of the intervention.

##### Types of settings

We examined the impact of changes to adult/child ratio and group size in formal ECEC settings with children aged 0–5 years old. Thus, we excluded studies of informal care arrangements such as private babysitters or family members. Furthermore, we excluded studies of children living in residential care arrangements such as foster families or institutions. The reason for excluding studies of children living in residential care arrangements was that our objective was to explore the impact of adult/child ratio and group size on the development and well‐being of children who were enroled in some form of formal non‐parental ECEC during the day, and not children being cared for around the clock by non‐parental caregivers.

### 4.2 Search methods for identification of studies

Relevant studies were identified through searches in electronic databases, governmental and grey literature repositories, Internet search engines, hand search in specific targeted journals, citation tracking, and contact to international experts.

#### 4.2.1 Electronic databases

We searched the following electronic databases:

- Socindex (through EBSCO)
- PsycINFO (through EBSCO)
- Econlit (through EBSCO)
- ERIC (through EBSCO)
- Teacher Reference Center (through EBSCO)
- Academic Search Premier (through EBSCO)
- Science Citation Index (through Web of Science)
- Social Science Citation Index (through Web of Science)
- Sociological Abstracts (through ProQuest)

Our selection of electronic databases was informed by Kugley, 2017.

All the primary searches on the electronic databases were performed between 23/01/2020 and 24/01/2020. Additional searches in PsychINFO and ERIC were carried out in February 2022.

#### 4.2.2 Electronic searches

The search string utilised to perform the searches contains three aspects, covering the population, the context of the intervention, and the intervention. We did not implement a facet for the study types due to the risk of over‐restricting the search. An example of the search strategy used for the PsycINFO database on the EBSCO‐host platform is shown below:

**Table:** 

S23	S7 AND S17 AND S22
S22	S18 OR S19 OR S20 OR S21 *INTERVENTION*
S21	DE ‘Class Size’
S20	AB (caretaker* OR teacher* OR staff* OR caregiver* OR adult*) AND AB ratio*
S19	AB ‘group size*’ OR ‘class size*’
S18	TI ‘group size*’ OR ‘class size*’ OR ratio*
S17	S8 OR S9 OR S10 OR S11 OR S12 OR S13 OR S14 OR S15 OR S16 *SETTING*
S16	((DE ‘Child Care’ OR DE ‘Child Day Care’) OR (DE ‘Kindergartens’)) OR (DE ‘Preschool Education’)
S15	AB (care N2 (center* OR centre* OR day* OR child*))
S14	TI (care N2 (center* OR centre* OR day* OR child*))
S13	AB (early N5 education)
S12	TI (early N5 education)
S11	AB ‘ECE’ OR ‘ECEC’ OR ‘ECCE’ OR ‘creche’ OR prekindergarten OR 'pre‐kindergarten’ OR ‘pre‐K’ OR ‘pre K’ OR ‘head start’ OR ‘community based child care’ OR ‘community‐based child care’ OR ‘center based child care’ OR ‘center‐based child care’ OR ‘family child care’ OR ‘home based child care’ OR ‘home‐based child care’
S10	AB preschool* OR ‘pre‐school*’ OR ‘non parental’ OR ‘non‐parental’ OR kindergarten* OR nurser* OR ‘early childhood education and care’
S9	TI ‘ECE’ OR ‘ECEC’ OR ‘ECCE’ OR ‘creche’ OR prekindergarten OR 'pre‐kindergarten’ OR ‘pre‐K’ OR ‘pre K’ OR ‘head start’ OR ‘community based child care’ OR ‘community‐based child care’ OR ‘center based child care’ OR ‘center‐based child care’ OR ‘family child care’ OR ‘home based child care’ OR ‘home‐based child care’
S8	TI preschool* OR ‘pre‐school*’ OR ‘non parental’ OR ‘non‐parental’ OR kindergarten* OR nurser* OR ‘early childhood’
S7	S1 OR S2 OR S3 OR S4 OR S5 OR S6 *POPULATION*
S6	(ZG ‘infancy (2‐23 mo)’) or (ZG ‘neonatal (birth‐1 mo)’) or (ZG ‘preschool age (2‐5 yrs)')
S5	DE ‘Preschool Students’ OR DE ‘Nursery School Students’ OR DE ‘Kindergarten Students’
S4	AB preschooler OR 'one‐year‐old*’ OR ‘one year old*’ OR ‘1 year* old*’ OR 'two‐year‐old*’ OR 'two year old*’ OR ‘2 year* old*’ OR ‘'three‐year‐old*’ OR ‘three year old*’ OR ‘3 year* old*’ OR 'four‐year‐old*’ OR 'four year old*’ OR ‘4 year* old*’ OR 'five‐year‐old*’ OR ‘five year old*’ OR ‘5 year* old*’
S3	AB infant* OR toddler* OR child* OR pupil* OR student* OR newborn* OR neonate* OR baby* OR babies
S2	TI preschooler OR ‘one‐year‐old*’ OR ‘one year old*’ OR ‘1 year* old*’ OR 'two‐year‐old*’ OR ‘two year old*’ OR ‘2 year* old*’ OR ‘'three‐year‐old*’ OR 'three year old*’ OR ‘3 year* old*’ OR ‘'four‐year‐old*’ OR 'four year old*’ OR ‘4 year* old*’ OR ‘five‐year‐old*’ OR ‘five year old*’ OR ‘5 year* old*’
S1	TI infant* OR toddler* OR child* OR pupil* OR student* OR newborn* OR neonate* OR baby* OR babies

A complete overview of the search strings and the search results for each electronic database and resource can be seen in the search documentation section of the appendix.

##### Limitations of the search‐string

No year or language restrictions were implemented in the database searches.

#### 4.2.3 Searching other resources

To identify relevant grey literature (dissertations, theses, working papers, conference proceedings, reports, government documents), we primarily utilised extensive searches on Google and Google Scholar. Furthermore, we searched specific resources for specified types of grey literature. The terms and search specifications for each resource can be found in the search documentation part of the appendix. When selecting outlets to search, we consulted the list of grey literature resources comprised in Kugley, 2017.

Most of the resources searched for unpublished literature contain multiple types of unpublished literature. For the sake of transparency, we have divided the resources into categories based on the type of literature expected to be most prevalent in the resource.

##### Search for reports, general grey literature and government documents

- Open Grey (http://www.opengrey.eu/)
- Google Scholar (https://scholar.google.com/)
- Google (https://www.google.com/)
- Social Care Online (https://www.scie-socialcareonline.org.uk/)
- OECD iLibrary—https://www.oecd-ilibrary.org/
- Eurydice Network ‐ https://eacea.ec.europa.eu/national-policies/eurydice/
- U.S. Department of Education ‐ https://www.ed.gov/
- Nordic Council of Ministers ‐ https://www.norden.org/en/nordic-council-ministers (searches made using English and Scandinavian language keywords, see Supporting Information Appendices).

##### Searches for dissertations

We searched the following resources for dissertations:

- Dissertations & Theses Global (through ProQuest)
- EBSCO Open Dissertations (through EBSCO)
- NB‐ECEC—Scandinavian research in early childhood education and care (https://www.nb-ecec.org/)

##### Searches for working papers and conference proceedings

The following resource(s) was (were) searched for working papers and conference proceedings:

- Social Science Research Network (https://www.ssrn.com/index.cfm/en/)
- European Educational Research Association (EERA)—https://eeraecer.de/

##### Search for existing systematic reviews or trials

We searched for existing systematic reviews that we could use for citation tracking. We searched the following resources:

- Campbell Systematic Reviews (https://onlinelibrary.wiley.com/journal/18911803)
- Cochrane Library (https://www.cochranelibrary.com/)
- Centre for Reviews and Dissemination Databases (https://www.crd.york.ac.uk/CRDWeb/)
- EPPI‐Centre Systematic Reviews—Database of Education Research (https://eppi.ioe.ac.uk/webdatabases/SearchIntro.aspx)
- Evidensbasen (The Evidence Base) https://dpu.au.dk/forskning/danskclearinghouseforuddannelsesforskning/evidensbasen/

The reviews we identified for citation tracking can be seen in the search documentation part of the appendix.

##### Hand searches

The journals we hand‐searched were selected during the pilot search process, in which we identified the journals with the highest frequency/hit rate in the pilot searches. Eighteen specific journals were hand‐searched for articles published within the last 2 years (September 2018 to September 2020). The 18 journals were:

- *Scandinavian Journal of Educational Research*
- *Nordic Studies in Education*
- *European Early Childhood Education Research Journal*
- *Early Child Development and Care*
- *Early Childhood Education Journal*
- *Journal of Early Childhood Research*
- *International Journal of Early Childhood*
- *International Research in Early Childhood Education*
- *Contemporary Issues in Early Childhood*
- *Journal of Early Childhood Teacher Education*
- *Child Care in Practice*
- *Childhood*
- *American Educational Research Journal*
- *Learning Environments Research*
- *Child Development*
- *Developmental Psychology*
- *Early Childhood Research Quarterly*
- *Early Education and Development*

##### Citation tracking

To identify both published studies and grey literature, we utilised citation tracking/snowballing strategies. Our primary strategy was to citation‐track related systematic reviews and meta‐analyses. The review team also checked reference lists of included primary studies for new leads.

##### Contact with international experts

We contacted or attempted to contact first authors of contemporary included primary studies, as well as authors of previous systematic reviews to identify unpublished and ongoing studies.

### 4.3 Data collection and analysis

#### 4.3.1 Selection of studies

Under the supervision of review authors, two review team assistants independently screened titles and abstracts and excluded studies that were clearly irrelevant. Studies considered eligible by at least one assistant or studies where there was insufficient information in the title and abstract to judge eligibility were retrieved in full text. The full texts were then screened independently by two review team assistants under the supervision of the review authors. Any disagreement of eligibility was resolved by the review authors. Studies were reviewed in any language which at least one member of the review team was able to read: Danish, Swedish, Norwegian, German and English.

**Figure 1:**
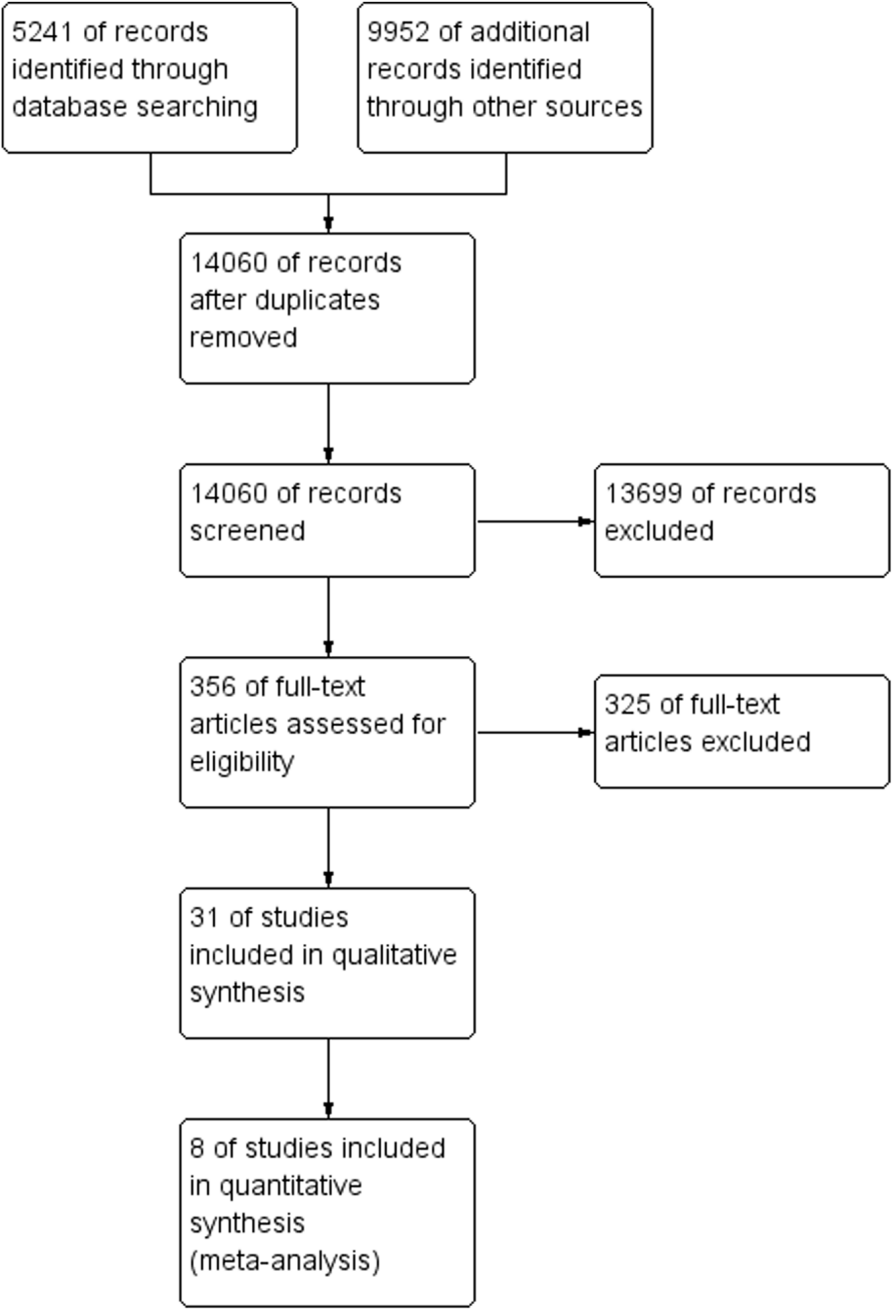
Study flow diagram

For a flow chart of the search and screening process, see Figure 1.

#### 4.3.2 Data extraction and management

Two review authors independently coded and extracted data from included studies. Disagreements were resolved by consulting a third review author with extensive content and methods expertise. For study level data, please see descriptive tables for Included studies. Data and information was extracted on: available characteristics of participants, intervention characteristics and control conditions, research design, sample size, risk of bias and potential confounding factors, outcomes, and results. Extracted data were stored electronically.

#### 4.3.3 Assessment of risk of bias in included studies

We assessed the risk of bias in randomised studies using Cochrane's revised risk of bias tool, ROB 2 (Higgins et al., 2019).

The tool is structured into five domains, each with a set of signalling questions to be answered for a specific outcome. The five domains cover different types of bias that can affect the results of randomised trials.

The five domains for individually randomised trials are:

(1) bias arising from the randomisation process;
(2) bias due to deviations from intended interventions (separate signalling questions for effect of assignment and adhering to intervention);
(3) bias due to missing outcome data;
(4) bias in measurement of the outcome;
(5) bias in selection of the reported results.

We assessed the risk of bias in non‐randomised studies using the model ROBINS–I, developed by members of the Cochrane Bias Methods Group and the Cochrane Non‐Randomised Studies Methods Group (Sterne et al., 2016a). We used the latest template (which was the version of 19 September 2016).

The ROBINS‐I tool is based on the Cochrane RoB tool for randomised trials, which was launched in 2008 and modified in 2011 (Higgins et al., 2011).

The ROBINS‐I tool covers seven domains (each with a set of signalling questions to be answered for a specific outcome) through which bias might be introduced into non‐randomised studies:

(1) bias due to confounding;
(2) bias in selection of participants;
(3) bias in classification of interventions;
(4) bias due to deviations from intended interventions;
(5) bias due to missing outcome data;
(6) bias in measurement of the outcome;
(7) bias in selection of the reported results.

The first two domains address issues before the start of the interventions and the third domain addresses bias due to misclassification of participants, that is, that some participants may be wrongly classified as being allocated to either the treatment or the control group. The last four domains address issues after the start of interventions and there is substantial overlap for these four domains between bias in randomised studies and bias in non‐randomised studies (although signalling questions are somewhat different in several places, see Sterne et al., 2016 and Higgins et al., 2019).

Randomised study outcomes are rated on a ‘Low/Some concerns/High’ scale on each domain, whereas non‐randomised study outcomes are rated on a ‘Low/Moderate/Serious/Critical/No Information’ scale on each domain. The level ‘Critical’ means that the study (outcome) was too problematic in this domain to provide any useful evidence on the effects of the intervention and we excluded it from the data synthesis. ‘Serious’ risk of bias in multiple domains in the ROBINS‐I assessment tool may lead to a decision of an overall judgement of ‘Critical’ risk of bias for that outcome and in this case, it was excluded from the data synthesis.

##### Confounding

An important part of the risk of bias assessment of non‐randomised studies is consideration of how the studies deal with confounding factors. Systematic baseline differences between groups can compromise comparability between groups. Baseline differences can be observable (e.g., age and gender) and unobservable (to the researcher; e.g., children's motivation and ‘ability’). There is no single non‐randomised study design that always solves the selection problem. Different designs represent different approaches to dealing with selection problems under different assumptions, and consequently require different types of data. There can be particularly great variations in how different designs deal with selection on unobservables. The ‘adequate’ method depends on the model generating participation, that is, assumptions about the nature of the process by which participants are selected into a programme.

A major difficulty in estimating causal effects of adult/child ratio and group size is the potential heterogeneity of both the different ECEC settings and of the children. In addition to the pre‐specified confounding factors, there may be unobservable factors affecting child development and well‐being, or selection mechanisms causing certain types of families to choose a specific ECEC setting for their child for reasons unavailable to the researcher.

As there is no universally correct way to construct counterfactuals for non‐randomised designs, we looked for evidence that identification was achieved, and that the authors of the primary studies justified their choice of method in a convincing manner by discussing the assumption(s) leading to identification (the assumption(s) that make it possible to identify the counterfactual). Preferably, the authors should make an effort to justify their choice of method and convince the reader that the children and settings with high versus low adult/child ratios and small vs. large group sizes were comparable.

In addition to unobservables, we identified the following observable confounding factors to be the most relevant: age/gender of the child, special needs status, structural characteristics of the ECEC setting (such as preschool, private or centre‐based care, educational level of teachers/caretakers), and socioeconomic background and ethnicity of the families (minority status or not). In each study, we assessed whether these factors had been considered, and in addition we assessed other factors likely to be a source of confounding within the individual included studies.

##### Importance of pre‐specified confounding factors

The motivation for focusing on age/gender of the child, special needs status, structural characteristics of the ECEC setting (such as preschool, private or centre‐based care, educational level of teachers/caretakers), and socioeconomic background and ethnicity of the families (minority status or not) is given below.

The younger the child, the more dependent the child is on stimulating adult/child interaction and basic nurture (Howes et al., 1992). Therefore, the impact of adult/child ratio and group size may vary depending on the age of the children, with younger children benefiting more from lower ratios and smaller group sizes than older children.

From a very early age, gender is associated with differences in child behaviour and cognition (Chaplin & Aldao, 2013; Ostrov & Keating, 2004; Silverman, 2003). Girls and boys in ECEC settings often show different toy and play preferences (Todd et al., 2017) and thus it is possible that gender may have an impact on what constitutes the best ECEC setting for each child.

Children with special needs such as physical or psychological disabilities are by definition considered to require more adult stimulation and care than children without any identified special needs and thus they may benefit more from an decreased adult/child ratio and smaller group sizes.

In previous research, other structural aspects of the ECEC settings have been found to be associated with both process quality and child outcomes (Cryer et al., 1999) and thus we consider the nature of the care setting (private vs. centre‐based daycare or preschool) as well as the educational level and continuous professional development of the teachers/caretakers to be potentially important confounders.

A large body of research documents the impact of parental socioeconomic background on almost all aspects of children's development (Renninger & Sigel, 2006), which is why we consider it important to control for this.

For children aged 0–5 years old, language acquisition is one of the most essential developmental tasks. Many ethnic minority children grow up to become bilingual and this may require more adult stimulation and interaction within ECEC settings. Thus, the potential impact of adult/child ratio and group size may vary depending on whether the child is monolingual or bilingual.

Children are often enroled in ECEC settings throughout the year based on their date of birth and not at a common point in time such as the beginning of the school year which would make the collection of true pre‐test scores (meaning pre‐enrolment scores) difficult. Therefore, we did not include pre‐test scores as a pre‐specified confounding factor. However, if pre‐test scores were available, these were taken into account when we evaluated the credibility of the between‐group comparability.

##### Assessment

At least two review authors independently assessed the risk of bias for each relevant outcome from the included studies. Any disagreements were resolved by a third reviewer with content and statistical expertise. For study level details on the assessment of risk of bias, please see the risk of bias table, which is available as a supplemental file.

#### 4.3.4 Measures of treatment effect

We did not include any dichotomous outcomes in the data synthesis. For continuous outcomes, we calculated effect sizes (ESs) with 95% confidence intervals (CIs) where means, adjusted means/regression coefficients, and standard deviations were available. If means and standard deviations were not available, we calculated standardised mean differences (SMDs) from *F*‐ratios, *t*‐values, *χ* ^2^ values and correlation coefficients where available, using the methods suggested by Wilson and Lipsey (2001). When the information was insufficient, we requested this information from the principal investigators, when these could be located. However, the only author who replied no longer had access to the data. We used Hedges' *g* for estimating SMDs. Hedges' *g* and its standard error are calculated as (Wilson & Lipsey, 2001, pp. 47–49):

(1) $$g=[1‐3/(4N-9)]\times \left(\beta /s_{p}\right),$$

(2) $$SE_{g}={\left[\left(N/\left(n_{1}n_{2}\right)\right)+\left(g^{2}/2N\right)\right]}^{0.5},$$

where *N* = *n*_*1*_ + *n*_*2*_ is the total sample size, *β* is an estimate of the intervention effect (e.g., the post‐intervention difference in means between the intervention and control group), and *s*_*p*_ is the pooled standard deviation defined as

(3) $$s_{p}={\left[\left((n_{1}-1)s_{1}^{2}+(n_{2}-1)s^{2})/(n_{1}-1+n_{2}-1\right)\right]}^{0.5}.$$

Here, *s*_*1*_ and *s*_*2*_ denote the raw standard deviation of the intervention and control group.

We used covariate adjusted means or regression coefficients for the intervention effect estimates and the unadjusted post‐test standard deviation whenever available. Because some studies did not include the pre‐intervention standard deviation, we used the post‐intervention standard deviation.

We used the same type of effect size measure for the single‐group repeated measures designs (as recommended by e.g., Lakens, 2013; Morris & DeShon, 2002). As the intervention group is its own control group in this design, standardisation with the intervention and control group post‐test standard deviation was not feasible. Instead, we calculated the effect size as (denoted Hedges' *g*_av_ in Lakens, 2013):

(4) $$g_{\mathrm{av}}=[1-3/(4N-9)]\times (M_{\mathrm{diff}}/[(sd_{1}+sd_{2})/2]),$$

where *M*_diff_ is the mean difference between an outcome measured at pre and post‐test, *sd*_1_ is the standard deviation at pre‐test, and *sd*_2_ is the standard deviation at post‐test. As the groups are not independent in the single‐group repeated measures design, it is not obvious how one should calculate a standard error for *g*_av_ that is comparable to *SE*_*g*_, and what *N* in the small sample correction in (4) should be. Hedges et al. (2013) suggested a version that for example takes into account the autocorrelation between the pre and post‐test. However, none of our included studies provided enough information to calculate this standard error. Instead, we opted for two versions with opposite assumptions: in our primary analysis, we calculated the standard error as for *g* with *n*_*1*_ = *n*_*2*_ = *n*, where *n* is the number of participants, and, consequently, *N* = 2*n*. Thus, we treated the pre and post‐test as if they were from independent groups. In a sensitivity analysis, we instead assumed that *n*_*1*_ = *n*_*2*_ = *n/2* and *N* = *n*.

We discuss further how and when we combined effect sizes from different research designs in the *Data synthesis* section and how we tested if our results were sensitive to combining effect sizes from different designs in the *Sensitivity analysis* section.

#### 4.3.5 Unit of analysis issues

To account for possible statistical dependencies, we examined a number of issues: whether the assignment of treatment was clustered, whether individuals had undergone multiple interventions, whether there were multiple treatment groups, and whether several studies were based on the same data source.

##### Clustered assignment of treatment

The assignment of treatment by cluster can result in an overestimation of the precision of the results (with a higher risk of a Type I error) if the unit of analysis is a within‐cluster unit (e.g., when the outcomes are child‐ or teacher‐level measures and the treatment is assigned by preschool). This was the case for all studies reporting child‐level language and literacy measures (Bleses et al., 2018; Francis & Barnett, 2019; Neuman & Kaefer, 2013) and two studies reporting process quality measures (Ruopp et al., 1980; Smith et al., 1989). We therefore used the formulas in Hedges (2007) to adjust the effect sizes and standard errors. As most studies did not report sufficient information to adjust them individually, and the few reported intra‐cluster correlations (ICCs) varied widely between and within studies, we used three ICCs. Our primary analysis included effect sizes and standard errors adjusted using an ICC = 0.1, and we report results from sensitivity analyses using ICCs ranging from 0 to 1. We assumed equal average cluster‐sizes in the intervention and control groups in all analyses.

##### Multiple intervention groups and multiple interventions per individual

Studies with multiple intervention groups with different individuals, and studies using multiple tests for the same intervention groups, were included in the review. To avoid problems with dependence between effect sizes, we used the robust variance estimation (RVE) methods developed by Hedges et al. (2010). We used the results in Tanner‐Smith and Tipton (2014) and Tipton (2015) to evaluate if there were enough studies for this method to estimate the standard errors reliably. That is, we report if the adjusted degrees of freedom are close to or below four, as Tanner‐Smith and Tipton (2014) and Tipton (2015) indicate that the standard errors are not reliable below this level. If the degrees of freedom were close to four, we conducted sensitivity analyses using study‐level average effect sizes and standard errors, and a restricted maximum likelihood (REML) estimation procedure with a Knapp and Hartung adjustment of standard errors (Knapp & Hartung, 2003; this procedure was recommended by e.g., Langan et al., 2019). We implemented the procedure using the *metafor* package in R (Viechtbauer, 2010; We describe these methods further in the *Data synthesis* section).

##### Multiple studies using the same sample of data

In some cases, several studies used the same sample of data or some studies used only a subset of a sample used in another study. We reviewed all such studies, but in the meta‐analysis we only included one estimate of the effect for each outcome from each sample of data. This means that if the same outcome was reported for a subgroup and for the full sample in separate studies, we only included the study using the full set of participants. In cases when two studies used the same sample (e.g., Francis, 2014; Francis & Barnett, 2019), we chose the study with the lowest overall risk of bias assessment or, if this assessment was the same, the most recent version.

#### 4.3.6 Dealing with missing data

Missing data in the individual studies was assessed using the risk of bias tool. Studies had to permit calculation of a numeric effect size for the outcomes to be eligible for inclusion in the meta‐analysis. Where studies had missing summary data, such as missing standard deviations, we derived these where possible from, for example, *F*‐ratios, *t*‐values, *χ* ^2^ values and correlation coefficients using the methods suggested by Wilson and Lipsey (2001). If these statistics were also missing, the review authors requested information from the study investigators.

If missing summary data necessary for the calculation of effect sizes could not be derived or retrieved, the study results were reported in as much detail as possible, that is, the study was included in the review but excluded from the meta‐analysis.

#### 4.3.7 Assessment of heterogeneity

We assessed heterogeneity with the *χ* ^2^ (*Q*) test, and the *I* ^2^ and *τ* ^2^ statistics (Higgins et al., 2003).

#### 4.3.8 Assessment of reporting biases

Reporting bias refers to both publication bias and selective reporting of outcome data and results. We assessed selective reporting as a part of the risk of bias assessment. We did not find a sufficient number of studies to construct funnel plots and thus we are unable to comment on the possibility of publication bias (Higgins & Green, 2011).

#### 4.3.9 Data synthesis

The overall data synthesis was conducted where effect sizes could be calculated. We performed multiple random‐effects meta‐analyses based on standardised mean differences (Hedges' *g*) and used the RVE procedure developed by Hedges et al. (2010). We used the *robumeta* package in R (Fisher et al., 2017) and the correlated effects weighting scheme to implement the RVE procedure. This weighting scheme uses estimates of the between and within‐study variance and an initial value of the within‐study effect size correlation (*ρ*) to calculate the weights used in the random‐effects analysis. We used the default value of *ρ* = 0.80 and conducted sensitivity tests with a variety of values to asses if the general results were robust to the choice of *ρ*. We also used the small sample adjustment to the residuals and the Satterthwaite degrees of freedom for significance tests in the RVE procedure (Tipton, 2015). We report 95% CIs throughout.

The corrections to the degrees of freedom enable us to assess when the RVE procedure performs well. As suggested by Tanner‐Smith and Tipton (2014) and Tipton (2015), if the degrees of freedom are fewer than four, the RVE results should not be trusted. As mentioned in the *Multiple intervention groups and multiple interventions per individual* section, when the degrees of freedom were below or close to four, we conducted analyses using study‐level average effect sizes and standard errors. We used the R package *metafor* (Viechtbauer, 2010) and the REML procedure with the Knapp and Hartung adjustment of standard errors (Knapp & Hartung, 2003) to conduct these analyses.

Different study designs may produce effect sizes that are not comparable. For example, in single‐group repeated measures designs, children and caregivers act as their own control group. In intervention‐control designs, other children and caregivers provide the estimate of the counterfactual situation in which the intervention group did not receive the intervention. As the standard deviation is based on a more homogeneous group of children/caregivers in single‐group designs than in intervention‐control group designs, there is a risk that the standard deviation is smaller in single‐group repeated measures designs. Consequently, effect sizes risk being inflated (i.e., the same effect will mechanically result in a larger effect size, if the standard deviation is smaller). However, if for example, time‐varying contextual factors have a strong influence on a measure, then there may instead be more variation in single‐group designs than in intervention‐control designs. Although the latter situation seems less likely in our case, it is difficult to rule out. It is also difficult to rule out the possibility that the standard deviations are approximately equal and that the two types of designs provide equally good estimates of the relevant counterfactual. We therefore included effect sizes from single‐group designs in our primary analysis. We tested the sensitivity of our results to this choice by analysing intervention‐control and single‐group designs separately.

In our primary analysis, we estimated the effects separately by two conceptual outcomes: process quality, and child language and literacy skills. We estimated the weighted average effect size with the RVE procedure and used meta‐regressions with a single indicator (i.e., just an intercept). We coded the effect sizes so that a positive coefficient represents beneficial effects of reductions to ratios and group sizes. That is, decreased adult/child ratios and decreased group sizes. Similarly, positive effect sizes represent beneficial effects of reducing ratios and group sizes in estimates from the REML procedure and the forest plots based on this procedure. (As some studies contributed a large number of effect sizes, it was difficult to produce legible forest plots based on the RVE procedure).

The resulting estimates mix large and small changes from different baselines of ratios and group sizes. In an analysis of this heterogeneity, described next, we used a strategy that took the size of the change into account. We also aimed to estimate the effects separately by intervention type (changes to adult/child ratio, group size, or both). However, no included intervention changed only the group size and due to the small number of included studies in each analysis, it was not possible to estimate the effects separately for interventions that either changed only the adult/child ratio or both the group size and the ratio. For the same reason, we could not estimate separate effects by categories defined by both the intervention type and the size of change to the adult/child ratio and group size, which our protocol specified (Dalgaard et al., 2020).

#### 4.3.10 Subgroup analysis and investigation of heterogeneity

Our primary analysis did not consider how much the included interventions changed the adult/child‐ratio. However, the magnitude of the change was heterogeneous across studies and ranged from 22% to 67%. To explore the association between the magnitude of the change and effect sizes, we used the following specification:

(5) $$g_{ios}=\beta_{1}\Delta AC_{ios}+e_{ios},$$

where *g*_*ios*_ is effect size *i* measured by outcome *o* from study *s*, *ΔAC*_*ios*_ is the difference in percent between the adult/child‐ratio in the intervention and control condition for this effect size, *β*_*1*_ is a parameter to be estimated, and *e*_*ios*_ is an error term (clustered by study in the RVE procedure).

A positive *ΔAC*_*ios*_ implied an improvement of the ratio. That is, increasing the ratio from one adult per five children to one adult per three children would imply a ([1/3 − 1/5]/(1/5)) × 100 = 66.7% increase.

Interventions sometimes intended to change the ratio by a certain amount but the children ended up receiving another ratio. Unfortunately, not all studies reported the intended ratio. We therefore used the received ratio for all studies, but our results were virtually identical if instead we used the intended ratio for those studies in which it was reported.

As mentioned, this analysis was explorative. As it entails a relatively strong assumption that the relation between the change in the ratio and effect sizes is linear and the received change in the ratio may have been influenced by unobserved factors that were related to both the magnitude of the change and the outcome, this analysis should not be given a causal interpretation. Furthermore, this analysis was not pre‐specified in our protocol. We were unable to conduct our pre‐specified moderator analyses because of the small number of included studies.

#### 4.3.11 Sensitivity analysis

We carried out a range of sensitivity analyses using the RVE procedure described in the *Data synthesis* section.

We restricted the sample to studies that had a low risk of bias to assess whether excluding studies with moderate or serious bias altered the main results of this review. We assessed the sensitivity of our results separately for each of the eight risk of bias domains.

We included both RCTs, QES, as well as single‐subject/repeated measures designs where all participants receive the treatment. Accordingly, we investigated how our results change, when we restrict the analysis to studies that use either of these designs. While we could have also investigated the sensitivity to combinations of design, such a fine‐grained sensitivity analysis would have resulted in a majority of sensitivity checks that only involved a single study. As mentioned in the *Measures of treatment effect* section, we also examined the sensitivity of our results to how we calculated the standard errors in single‐group designs.

We included studies that estimated effect sizes by using the observed mean values or by using adjusted mean values. We assessed how sensitive our main results were to differences in estimation methods by conducting analyses in which we restricted the included studies to only use one of the two estimation methods.

The RVE procedure has the distinct advantage of accounting for the dependence between effect sizes that arises when studies contribute multiple effect sizes. However, the procedure relies on specifying the correlation between effect sizes within studies. In the primary analysis, we used the default value of 0.8 and we investigated if the results were sensitive to the choice of *ρ* value.

As discussed in the *Unit of analysis issues* section, we used a fixed value of the ICC (0.1) to adjust effect sizes and standard errors from studies that analysed outcomes on a lower level than the unit of treatment assignment. To examine the sensitivity of our results to this choice, we conducted analyses using the full range of possible values of ICCs, that is, from 0 to 1.

The sensitivity checks were generally restricted by the small number of studies. We followed our protocol (Dalgaard et al., 2020) to the extent it was possible, but some pre‐specified analyses were not feasible or sensible in this review, as there are either none or just a single study available. For example, restricting the analysis to only include studies with ‘low risk of bias’ on a given risk of bias item sometimes reduced the sample to one study or no studies at all. We refrained from presenting the results from these analyses. Similarly, the protocol for this review stated that we would include both study design and estimation method as moderators in a meta‐regression model. Due to the small number of included studies, this sensitivity check was unfeasible however.

##### Treatment of qualitative research

This review does not include qualitative research.

#### 4.3.12 Summary of findings and assessment of the certainty of the evidence

Findings of the review were summarised and the certainty of the evidence was assessed as outlined in the protocol for the review Dalgaard 2020.

## 5 RESULTS

### 5.1 Description of studies

#### 5.1.1 Results of the search

We summarise the results of the search and screening process in Figure 1 in the appendix. The total number of potentially relevant records was 14,060 after excluding duplicates (database: 5241, grey, hand search, snowballing and other resources: 9952). All records were screened based on title and abstract; 13,699 were excluded for not fulfilling the screening criteria, 5 records were unobtainable despite efforts to locate them through libraries and searches on the Internet, and 356 records were ordered, retrieved, and screened in full text. Of these, 325 did not fulfil the screening criteria and were excluded. A total of 31 studies were included in the review. The references are listed in the section *References to included studies*.

#### 5.1.2 Included studies

The search resulted in a final selection of 31 studies, which met the inclusion criteria for this review. We present descriptive statistics for the included studies in Tables 1 and 2. The 31 studies analysed 26 different populations. Only 12 studies (analysing 8 different populations) could be used in the data synthesis. The remaining studies were not usable in the meta‐analysis for multiple reasons. For some studies there was more than one reason for exclusion from the meta‐analysis. Twelve studies studies could not be used in the data synthesis as they were judged to have too high risk of bias. Seven studies did not provide enough information to calculate effect sizes or standard errors, or did not provide results in a form we could use in the data synthesis. Three studies did not provide means/and or SE permitting us to calculate an effect size: Field (1980), Phillips and Twardosz (2003) and Love (1993). We attempted to contact the latter two authors and received a reply from Phillips and Twardosz (2003) stating that data was no longer available. In four studies, outcomes were judged to be ambiguous: Endsley (1973), McCabe et al. (1996), Brownell and Smith (1973), Pelligrino and Scopesi (1990). By ‘ambiguous outcomes’ we mean descriptions of child and adult behaviour in which it is unclear if higher/lower scores are beneficial/adverse or vice versa. Finally, of the 12 studies that could be used in the data synthesis, two pairs of studies used the same data set (Francis, 2014; Francis & Barnett, 2019) and (Smith et al., 1988, 1988) reported on the same outcome(s), and four studies used the same data set from the National Daycare Study and reported on the same outcomes (Asher, 1979, Smith & Spence, 1980; Travers et al., 1980) and thus in addition four studies were not used in the data synthesis, see below. Included studies were from Australia (1), Denmark (1), England (1), Italy (1), Korea (1), New Zealand (2), Portugal (1), Sweden (2), The Netherlands (1), and USA (20). Included studies were published between 1968 and 2019, and the publication year average was 1992.

**Table 1:** Summary risk of bias score ROBINS‐I

Judgement: Risk of bias item:	Low	Moderate/some concerns	Serious	Critical	Unclear/no information	Number of studies
Overall Judgement		8	7	12	1	28
Confounding Bias	2	9	5	9	3	28
Selection Bias	10	8	4	2	4	28
Classification Bias	17	7	3	1		28
Deviation Bias	9	9	5		5	28
Missing Data	9	5	3	2		28
Measurement Bias	7	11	8	1	1	28
Reporting Bias		19	7		2	28

**Table 2:** Summary risk of bias score ROB 2

Judgement: Risk of bias item:	Low	Some concerns	High	Unclear/no information	Number of studies
Overall Judgement		1	2		3
Randomisation Process	1		2		3
Deviations from intervention	1		2		3
Missing Data		3			3
Measurement of Outcome	1	2			3
Selection of Reported Results		3			3

#### 5.1.3 Excluded studies

Eight studies initially appeared eligible but were excluded with reasons. Please see the reference list for specification.

### 5.2 Risk of bias in included studies

The risk of bias coding for each of the 31 studies is available as an appendix.

Summary scores from the risk of bias assessment can be seen in Tables 1 and 2. Three studies reported outcomes from two RCTs, and thus were rated using the ROB 2 tool, whereas the remaining 28 studies were rated using the ROBINS‐I tool. No study had an overall low risk of bias, and only one study cited an a priori protocol or an a priori analysis plan. Two studies reporting on the same RCT had a high risk of bias due to problems with the randomisation and deviations from the intervention. One study (reported e.g., in Travers et al., 1980) was intended as an RCT but due to large scale differences between assigned and received treatment and some nonrandom assignment of treatment, we considered this study a QES and assessed the outcomes by ROBINS‐I.

We rated 12 QESs as having overall critical risk of bias. Nine of these studies had a critical risk of confounding bias, two studies had a critical risk of selection bias, one study had a critical risk of classification bias, two studies had a critical risk of bias caused by missing data and one study had a critical risk of measurement bias. Among the studies, which were included in the meta‐analysis, no study had an overall low risk of bias, five studies had a some concerns/moderate risk of bias, and three studies had high/serious risk of bias.

We assessed the risk of bias score separately for each outcome in included studies, however, we did not include any studies in which the judgement differed between outcomes.

### 5.3 Effects of interventions

#### 5.3.1 Synthesis of results

The meta‐analysis using measures of process quality as the outcome is based on five studies exploring different changes to group size and/or adult/child ratio in ECEC. We used the lowest ratio (most children per adult)/largest group size condition as the control condition in the analyses.

Russell (1990) explored the effects of small changes in child/staff ratio on observed child/staff behaviour in 27 preschools. The number of children was manipulated to produce a low ratio (7.7:1), an average ratio (9.2:1), and a high ratio (11.2:1). The total number of children was 675 and two teachers and one aide from each of the 27 preschools participated.

De Schipper et al. (2006) used a single‐group repeated measures design, in which the child‐caregiver ratio was manipulated by changing the number of children assigned to the same caregiver during two play episodes in the same classroom. The adult/child ratios in this experiment were 1:3 and 1:5. In total, 217 caregivers from 64 daycare centres participated.

Francis and Barnett (2019) used an experimental approach in which class size in preschool was reduced randomly for either an AM or a PM session for each teacher participating. In the reduced class sizes (treatment group), class size was intended to be 15, but the observed average was 12.61. In regular class sizes (control group), the intended class size was 20, but the observed average was 16.23. Children from 40 sessions in 20 classrooms were observed and each classroom had a lead teacher and an assistant teacher. In total, 161 children were observed in the reduced classrooms and 193 children were observed in regular classrooms.

Travers et al. (1980) report results from the National Day Care Study, a 49‐centre QES conducted across three study sites (Atlanta, Detroit, and Seattle) which compared three groups of centres (treatment, untreated low‐ratio, and untreated high‐ratio). The treatment group ratio was 1:5.9. In the comparison groups, the ratios were 1:9.1 (untreated high‐ratio centres) and 1:5.9 (low‐ratio centres). Our meta‐analysis contrasts the treatment and high‐ratio conditions. In total, 210 caregivers were observed in the Fall and 220 in the Spring. Similarly, 1310 children were observed in the Fall and 1108 in the Spring. It should be noted that we were able to use only one of the reported outcomes from this study. Similarly, we were unable to use any of the outcomes from an intervention in which children were originally randomised to classrooms which differed in terms of staff education and adult/child ratio, in the meta‐analysis. The reason was that we lacked information to calculate effect sizes.

Smith et al. (1988) used a quasi‐experimental approach in which four experimental and four control kindergartens were selected for participation in two locations. Experimental kindergartens hired a third kindergarten teacher while control kindergartens continued with their usual staffing of two teachers. Thirty‐five children and 13 teachers were observed across all three data collection sessions.

The meta‐analysis of process quality measures included 84 effect sizes, 5 studies, and 6256 observations. The weighted average effect size was positive but not statistically significant (effect size = 0.10, 95% CI = [−0.07, 0.27]). The adjusted degrees of freedom were below 4 (*df* = 1.5), meaning that the results were unreliable. Similarly, the low number of studies made the estimation of heterogeneity statistics difficult. The *I* ^2^ and *τ* ^2^ estimates were both 0, and the *Q*‐statistic 2.3 (*p* = 0.69).

The meta‐analysis of child level language and literacy outcomes is based on three studies exploring different changes to group size and/or adult/child ratio in ECEC. Francis and Barnett (2019), which was described above, also used measures of language and literacy outcomes.

Bleses et al. (2018) used a cluster‐randomised design to evaluate three variations of a language‐literacy focused curriculum (LEAP), in which adult/child ratios differed between 1:5 and 1:8. In total, 5436 3–6‐year‐old Danish children from 154 daycare centres in 8 municipalities participated.

Neuman and Kaefer (2013) used a single‐group repeated measures design to evaluate an 8‐week structured language intervention, in which each child received instructions on sets of words in a whole group (4 weeks) and small group (4 weeks). Group size in the small group conditions was 4‐5 children and 18 children in the whole group condition (on average). In total, 108 children participated.

The meta‐analysis of language and literacy measures included 12 effect sizes, 3 studies, and 14,625 observations. The weighted average effect size was negative but not statistically significant (ES = −0.04, 95% CI = [−0.61, 0.53]). The adjusted degrees of freedom were again below 4 (*df* = 1.9) and the results were unreliable. The heterogeneity statistics indicated substantial heterogeneity—*Q* = 9.3 (*p* = 0.009), *I* ^2^ = 78.5%, *τ* ^2^ = 0.07—but due to the low number of studies, these results should be viewed with caution.

Because the number of studies was low in both meta‐analyses, the RVE procedure we used may have problems estimating the standard errors and the heterogeneity statistics reliably. We therefore estimated alternative models using study level average effect sizes and an REML procedure with Knapp and Hartung adjusted standard errors. Using study level averages also allowed us to create legible forest plots for both types of measures.

The point estimates of the average effect sizes were close to the estimates from the RVE procedure in both cases (ES = 0.10 and ES = −0.06 for process quality and language and literacy, respectively). The effect estimate on process quality was significant (95% CI = [0.004, 0.20]), which the estimate for language and literacy was not (95% CI = [−0.57, 0.46]). The heterogeneity statistics indicated low levels of heterogeneity across process quality effect sizes (*Q* = 0.7 (*p* = 0.946), *I* ^2^ = 0%, *τ* ^2^ = 0) and relatively high levels across language and literacy effect sizes (*Q* = 6.1 (*p* = 0.048), *I* ^2^ = 64.3%, *τ* ^2^ = 0.03). The small number of studies still implies that these results should be viewed with caution.

**Figure 2:**
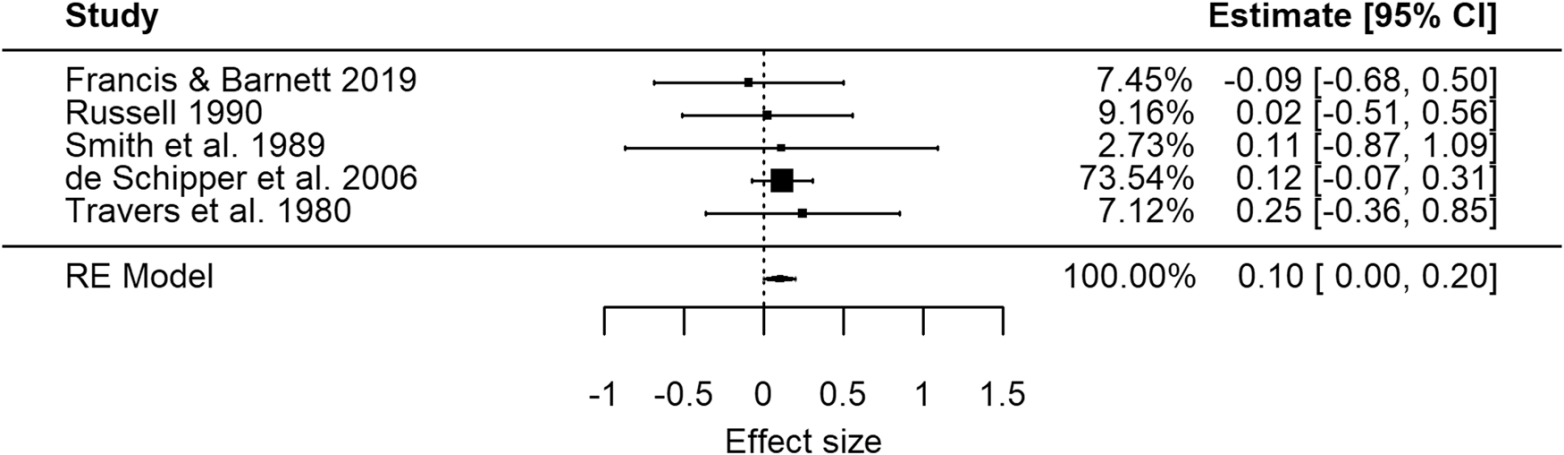
Forest plot of study level average effect sizes based on process quality measures

**Figure 3:**
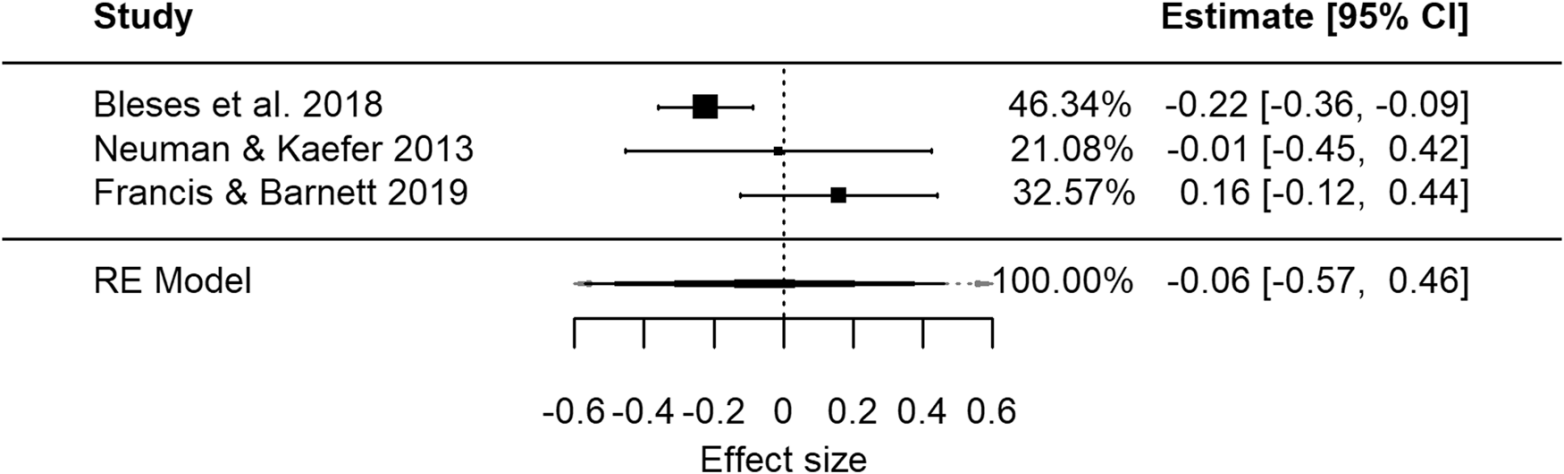
Forest plot of study level average effect sizes based on language and literacy measures

The forest plots shown in Figures 2 and 3 provide another illustration of the variation in effect sizes. The five process quality effect sizes ranged from −0.09 to 0.25 and the three language and literacy effect sizes ranged from −0.22 to 0.16. Figure 2 furthermore indicates that the estimate of the process quality effect size was heavily influenced by one study (de Schipper et al., 2006), which received a weight of 84.4% in the analysis.

A further, more qualitative, limitation to the results is that two interventions changing the adult/child ratio and group size did not occur in the everyday context of an ECEC program, but were implemented in the context of a specific language intervention (Bleses et al., 2018; Neuman & Kaefer, 2013). It is noteworthy that the one study that measured language and literacy outcomes and examined an intervention occurring in the everyday context of an ECEC program found a positive average effect (Francis & Barnett, 2019, see Figure 3). Furthermore, some interventions were relatively short, lasting at most a few weeks (e.g., Neuman & Kaefer, 2013; Russell, 1990), and in one case the outcomes were measured during a 10 min session of structured play (de Schipper et al., 2006).

#### 5.3.2 Results of the subgroup analysis and investigation of heterogeneity

The magnitude of the change in the adult/child‐ratio was positively associated with the process quality effect sizes (*β* = 0.0018, 95% CI = [−0.00029, 0.0039]), and negatively associated with the language and literacy effect sizes (*β* = −0.0020, 95% CI = [−0.020, 0.016]). The degrees of freedom were again below four. As in the primary analysis, we therefore used the REML procedure with study‐level effect sizes. This procedure yielded very similar results but the process quality estimate was statistically significant (*β* = .0018, 95% CI = [0.0005, 0.0031]) for process quality, and *β* = −0.0024, 95% CI = [−0.011, 0.0061] for language and literacy). The estimates imply that a 10% lower adult/child‐ratio was associated with, for example, a 0.018 increase in effect size for process quality measures.

The signs of the associations were thus in line with the signs of the weighted average effect sizes in the primary analysis. Although the changes in the received ratio were not always as large as the researchers intended, all interventions produced reduced adult/child‐ratios in the intervention group (the range of reductions was between 22% to 67%). Thus, the results in the primary analysis were not caused by failed interventions that did not change the adult/child‐ratios, or changed them in the wrong direction.

In the next section, we report the results of the sensitivity analysis described earlier in the Sensitivity analysis section.

#### 5.3.3 Results of the sensitivity analysis

##### Results across ROB items

**Figure 4:**
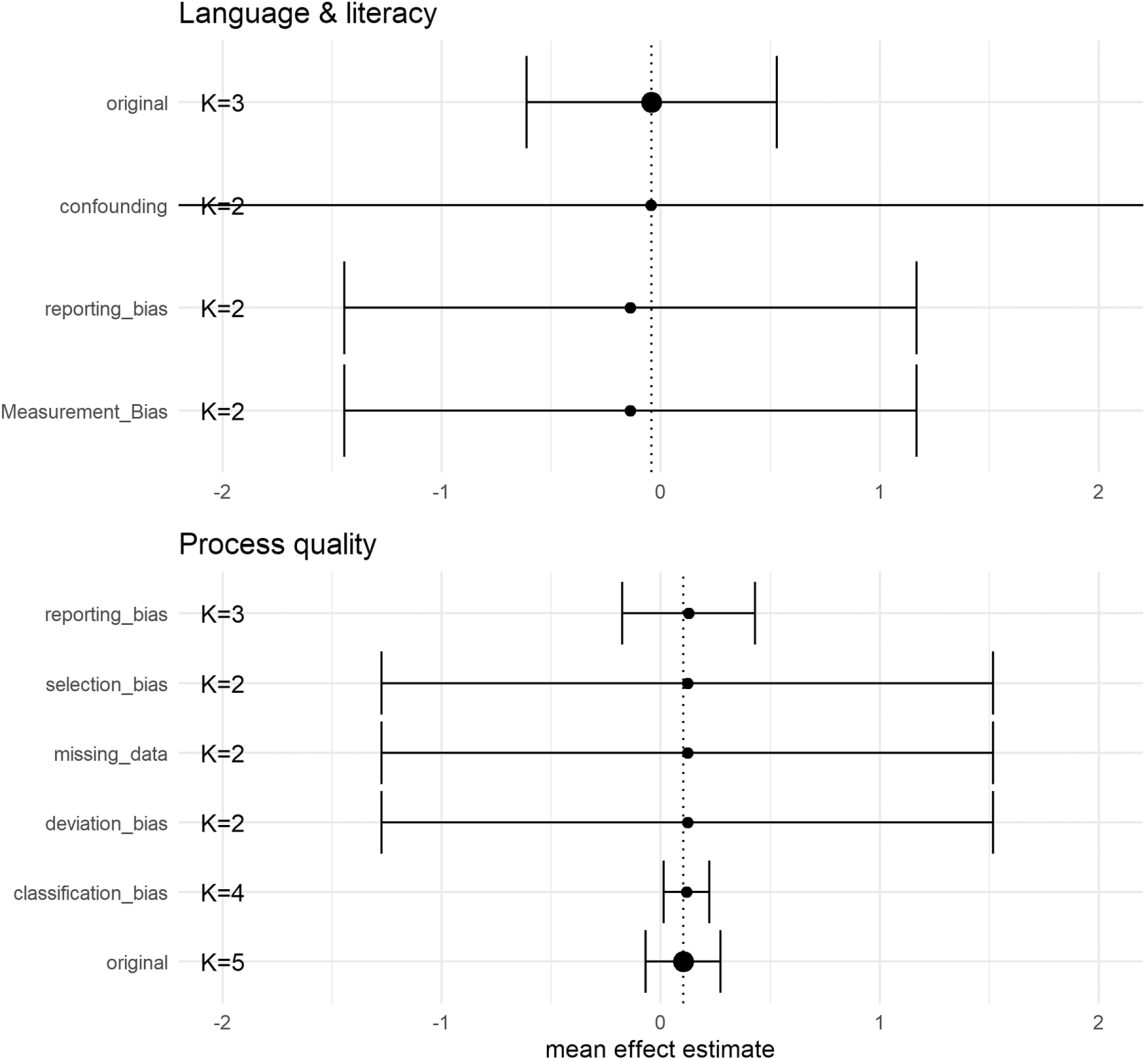
Mean effect sizes in analyses restricted to low risk of bias outcomes

Figure 4 shows the results of the sensitivity analysis concerning the sensitivity to risk of bias. In Figure 4, the points represent the mean effect sizes of the meta‐analysis, while the error bars represent the 95% CIs. Restricting the analysis to effect sizes assessed to have a low risk of bias changed the point estimate of the mean effect size in two cases for the language and literacy outcomes. In both, the mean effect size became more negative. For process quality outcomes, the changes were small. Restricting the analysis in this way reduces the sample size even further, and in several cases, the analysis is limited to just two studies. This reduction in sample size generally results in wider CIs and none of the estimates are statistically significant. Further, while we have not conducted a formal statistical test of the difference between the estimates from the primary analysis and those reported in Figure 4, given the lack of statistical precision, it is unlikely that we could detect any statistically significant difference between estimates of the mean effect.

##### Sensitivity to study design

**Figure 5:**
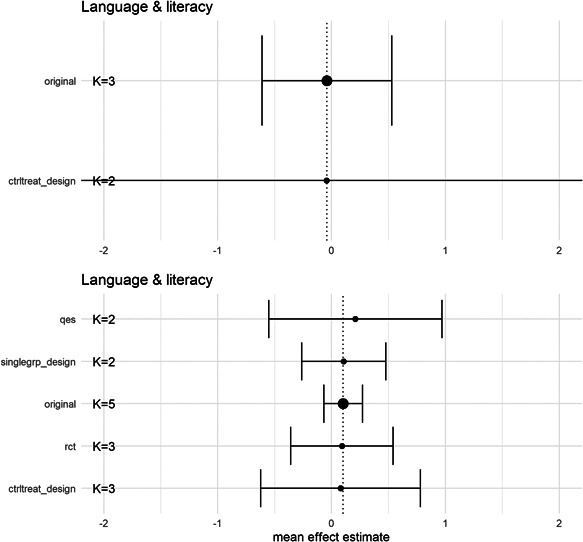
Sensitivity to study design

In Figure 5, there is one instance when the estimate changes in size when we restrict the analysis to studies of a certain design. The two QES have a larger mean effect size than in the primary analysis of process quality outcomes. However, while this estimate is roughly a doubling of the estimated mean effect size, the CI is wide and we thus lack the precision to provide strong evidence against the original analysis. As such, we can neither conclude that our original analysis is sensitive to the design applied by the included studies, nor that it is not sensitive.

We also examined the sensitivity of our results to how we calculated the standard errors in single‐group designs. In this analysis, we recalculated the standard errors using half the number of participants. Using these new standard errors implied in very small changes of both the process quality and the language and literacy results (ES = 0.10, 95% CI = [−0.07, 0.27], and ES = −0.04, 95% CI = [−0.70, 0.62], respectively).

##### Sensitivity to estimation methods

**Figure 6:**
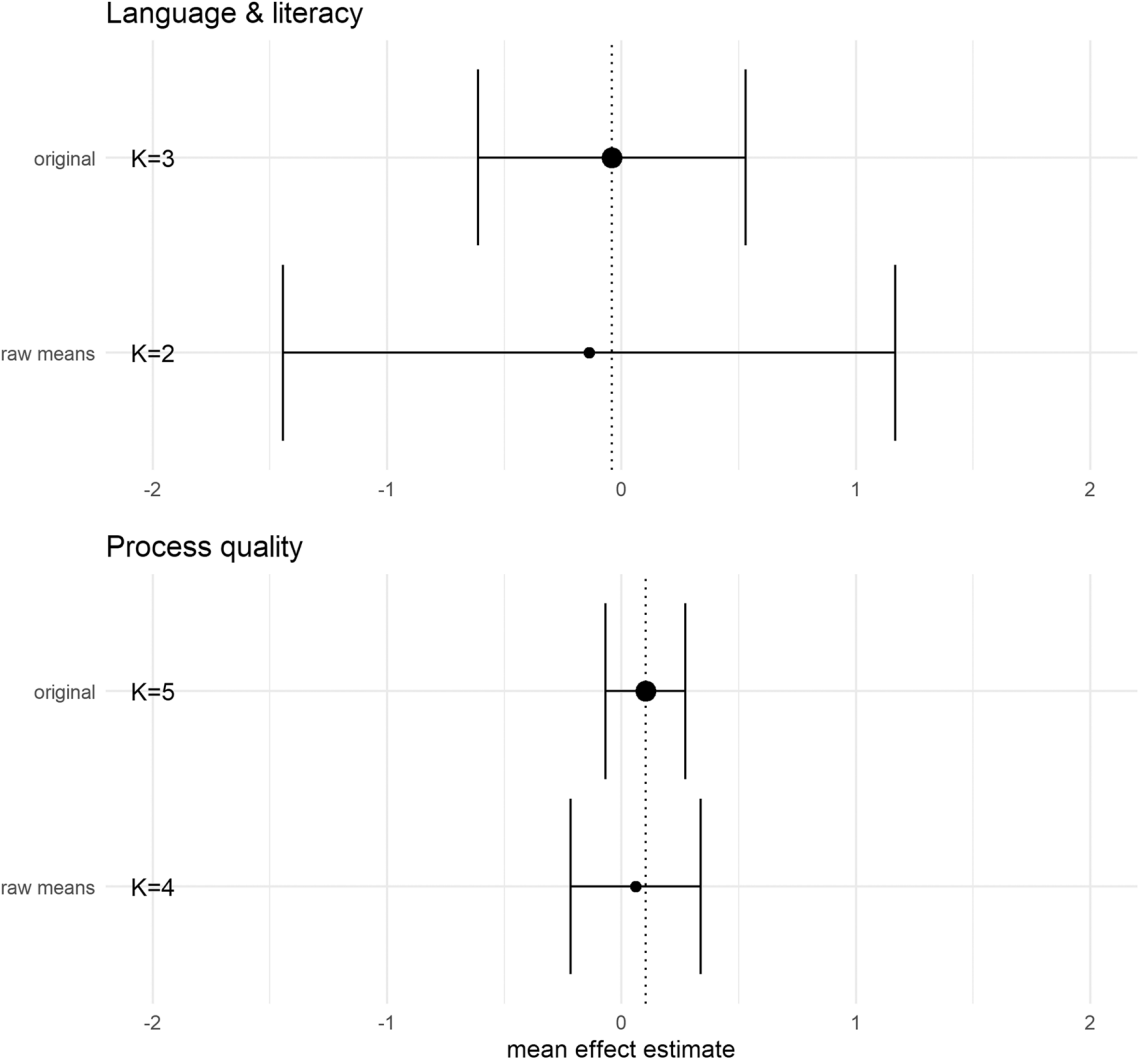
Sensitivity to estimation method

As can be seen in Figure 6, effect sizes estimated using the raw means yielded lower effect sizes for both process quality, and language and literacy outcomes. However, as for the previous sensitivity checks, we lack the statistical precision to conclude whether these estimated effect sizes were different than 0 or if they were different from the primary analysis.

##### Sensitivity to *ρ* when fitting RVE meta‐analytic models

**Figure 7:**
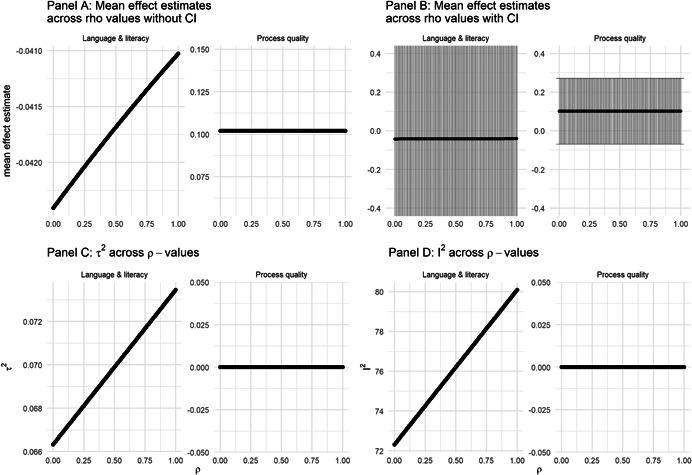
Sensitivity to rho‐values

As can be seen from the results in Figure 7, changing the value of the *ρ* parameter does change the estimate of the mean effect as well as the estimated heterogeneity. However, one should direct attention towards the scale of the y‐axis in the plots. Here we can see that the differences between the smallest and the largest values for both the mean effects and heterogeneity are very small. Regardless of the *ρ*‐value chosen, the estimated mean effects for the language and literacy effect sizes are small and negative, and there is a relatively large amount of heterogeneity present. For the process quality effect sizes, the mean effect continues to be positive, and the estimated heterogeneity continues to be very small. Only at very high levels of *ρ* do we see a slight change for the process quality effect sizes, but the change is not large enough to alter any conclusions.

##### Sensitivity to values of intraclass correlation for cluster randomised studies

**Figure 8:**
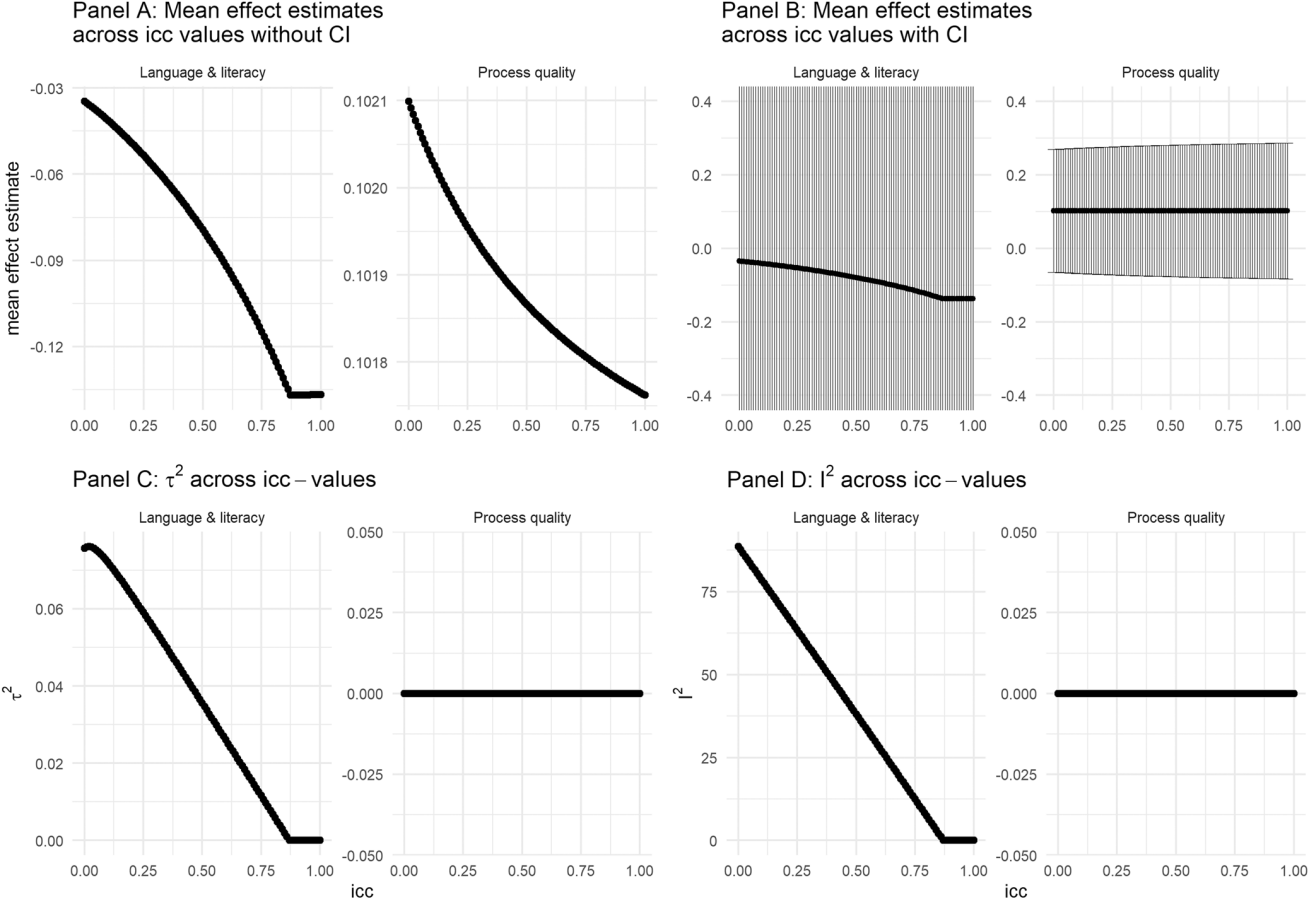
Sensitivity to the choice of ICC value

In the primary analysis, we specified a fixed value of the ICC, 0.1, and used this value to adjust all effect size estimates. In this section, we investigate whether specifying a different value of ICC change the results of the primary analysis. We report the results in Figure 8.

While at first the plots in panels A, C, and D may suggest that changing the ICC values does change the results, it is important to draw attention to the y‐axis of the plots. Here we see that the difference between the largest and smallest value is small, unless we increase the ICC to very high levels (>0.5) and even so, the difference is only visible for language and literacy outcomes. Further, panels C and D indicate that the heterogeneity estimates for language and literacy outcomes are reduced, the more we increase the ICC. The ICC has no impact on the heterogeneity estimates for process quality outcomes. Thus, the results in the primary analysis do not appear to be particularly sensitive to the choice of ICC‐value.

##### Summary of sensitivity analysis

In summary, we found some indications that the estimated mean effects were sensitive to the risk of bias assessment of studies and the estimation method, and, for process quality outcomes, the study design of the effect sizes. Neither the estimated mean effect nor estimated heterogeneity showed any substantial sensitivity to the choice of *ρ*‐value in the RVE procedure or the choice of ICC value when adjusting for clustered assignment of treatment/control conditions.

However, the sensitivity checks are limited by the small sample size in this review. The changes in estimates that we are seeing may as well be a result of omitting or limiting the analysis to one or two influential studies as much as it is the result of restricting the analysis to a certain type of study (e.g., only RCTs). Additionally, the characteristics of the studies investigated here, risk of bias, study design, and so forth, may be confounded. It was not possible to fit all the characteristics investigated here as moderators in a meta‐regression model, however. In other words, the sensitivity checks could not provide strong evidence that the estimated mean effects differed from the primary analysis, nor that they did not. But the checks do imply that we should be cautious when interpreting the results.

## 6 DISCUSSION

### 6.1 Summary of main results

Our main finding was that more internally and ecologically valid studies of adult/child ratios and group size are needed. We could only include information from 12 studies covering 8 different populations in the meta‐analyses. Furthermore, several included studies examined children and caregivers in contexts that were not representative of everyday life in ECEC programs. We found no studies of long‐term effects and the duration of the intervention was short in a few studies. No study changed the group size while keeping the adult/child‐ratio constant, and no study examined child level socio‐emotional outcomes.

In our meta‐analysis, we found a positive and, in one analysis, statistically significant average effect of reducing the adult/child‐ratio and group size on outcomes measuring process quality (ES = 0.10). We found no evidence of heterogeneity of the process quality effect sizes. The average effect on outcomes measuring children's language and literacy skills was negative (ES = −0.04) but with wide CIs regardless of estimation method. We also found evidence of heterogeneity among the language and literacy effect sizes, and two out of three interventions had relatively low ecological validity. Our sensitivity analyses were limited by the small number of studies, but indicated lack of robustness in some areas.

Although our meta‐analytic results were uncertain, it may still be of interest to discuss the magnitude of the effects. Effect sizes based on standardised means are not easy to interpret. In addition, our results indicated that larger changes of the adult/child ratio and group size were associated with larger effects, which means that the magnitude of the intervention may also be important for the interpretation of the effect sizes. Therefore, we provide a few ‘translation’ examples for the process quality measures, where we express the effect sizes of an intervention that is, in our sample, typical regarding both the ratio change and the effect using scales that we believe are easier to interpret. Due to the small number of studies, the heterogeneity, and the lack of ecological validity, we refrained from providing examples for the language and literacy measures.

We used the estimate of the association between the percent change in the ratio and process quality effect sizes (0.0018), and the median change of the adult/child ratio across interventions (a 45.5% decrease in the ratio) in the examples. We chose the outcome measures in Russell (1990), which we believed were easiest to interpret in terms of being beneficial/harmful. Russell (1990) measured process quality by the frequencies of behaviour in the observed child and caregiver group. We used the pooled standard deviation for each outcome measure to convert the estimate of the association to a change in the frequency, and to a proportion of the control group mean. The results imply that a typical intervention was, depending on the measure, associated with reductions of 0.3–1.6 percentage points (22%–65% of the control group mean) of the frequency of waiting passively, aimless wandering, annoying/teasing behaviour, and being disciplined, and with increases of 0.4–0.7 percentage points (26%–64%) of the frequency of praise, giving affection, and cooperative behaviour.

Although the change in the frequencies of behaviour of a typical intervention were mostly small, the relative effects seem larger and the magnitude of the effects were in our view educationally or developmentally meaningful. Especially considering that the duration of most interventions was short. This translation exercise was intended to help the interpretation and discussion of effect sizes found in the literature on adult/child ratios and group sizes in ECEC, not to imply that the associations we estimated were the true effects. As mentioned in the *Subgroup analysis and investigation of heterogeneity* section, the analyses rests on some strong assumptions and so do the calculations above.

### 6.2 Overall completeness and applicability of evidence

We performed a comprehensive electronic database search, combined with grey literature searching, and hand searching of key journals. All citations were screened by two independent screeners from the review team (FLW, KGE, MHC, MWK), and one review author (NTD) assessed all included studies against inclusion criteria.

We believe that all the publicly available studies on the effect of changes to adult/child ratio and group size in ECEC up to the censor date were identified during the review process. However, five references were not obtained in full text and two studies provided insufficient information to permit us to calculate an effect size. Despite attempts to contact the authors of these two studies with insufficient information, these could not be included. One author (Love, 1993) could not be located and one author replied that she no longer had the data and thus a standard error could not be calculated (Phillips & Twardosz, 2003).

### 6.3 Quality of the evidence

The overall quality of the included studies was low and we only included two randomised studies in the meta‐analysis. The risk of bias in the majority of included studies was high even in the studies used in the meta‐analysis; three studies (Francis & Barnett, 2019; Smith, 1988; Smith & Connolly, 1986) were judged to be at high/serious risk of bias. Due to the limited number of eligible studies that could be used in the data synthesis, we were unable to explore the effects of adult/child ratio and group size separately. Furthermore, only one randomised study measured process quality as an outcome (Francis, 2014; Francis & Barnett, 2019), and the intervention in this study was a relatively small change in adult/child ratio and group size (20 students vs. 15 students). Thus, no high‐quality study explored the effects of large changes in adult/child ratio and group size on measures of process quality, and not a single high‐quality study explored the effects of any changes in adult/child ratio and group size on socio‐emotional child outcomes. On average, the studies were almost 30 years old, and not a single high‐quality study explored the effects of adult/child ratio and group size in ECEC for children younger than 2 years of age. Given that the mainstream theory and guidelines, in the field of ECEC, suggest that younger children may benefit even more from increased adult‐child interaction, the complete lack of included studies regarding children younger than 2 years of age seems quite remarkable

### 6.4 Potential biases in the review process

We are unable to comment on the possibility of publication bias as at most five studies were included in the same meta‐analysis. Thus, we cannot rule out that there are still some missing studies.

We believe that there are no other potential biases in the review process as two members of the review team independently coded the included studies. Any disagreements were resolved by discussion. Further, decisions about inclusion of studies were made by two members of the review team and one review author. Assessment of study quality and numeric data extraction was made by the review authors (JSD, RHK, AB, and NTD) and was checked by a second review author.

### 6.5 Agreements and disagreements with other studies or reviews

Perlman et al. (2017) conducted a systematic review and meta‐analysis of child‐staff ratio in ECEC settings on child outcomes. The purpose of this systematic review was to evaluate the association between child‐staff ratios and children's outcomes. Searches revealed 29 relevant studies, with only three studies eligible for inclusion in the meta‐analysis. These three studies focused exclusively on associations between child/staff ratios and children's receptive language, thus not allowing for broader conclusions regarding child outcomes in other areas, for example, inter‐personal skills or child well‐being. Perlman et al. noted that the methodological properties of studies within the ECEC literature may pose a challenge to researchers wishing to conduct statistical meta‐analyses. The methodological issues encountered by Perlman et al arose from e.g., the operationalisation of child‐staff ratios, the child outcome domains measured, the psychometric properties of outcome measures and overall study design, leading the authors to call for more comparative effectiveness research designs, such as prospective cohorts or cluster randomised studies (Perlman et al., 2017). In the present review, we encountered similar methodological challenges, and in line with Perlman's conclusion, we want to emphasise that results of the present review should not be interpreted as an indication that adult/child ratio and group size in ECEC do not have an impact on child outcomes. Results of the present review may be seen as a confirmation of the fact that very few if any high‐quality studies have examined this question, and that there is an urgent need for more contemporary research in this field.

Bowne et al. (2017) conducted a meta‐analysis of class sizes in early childhood education programs based on a sample consisting of 38 evaluation studies of ECEC programs published between 1960 and 2007. The effect sizes in this meta‐analysis assessed the overall impact of each program compared to a passive control group (i.e., in children who did not attend the program), and the analyses estimate differences in the effect size impacts by the reported class sizes and child–teacher ratios. Results are thus not directly comparable to findings from the present review, but in line with findings regarding the very limited number of included studies within the present review, the authors state that they did not include studies that compared two alternative ECEC programs, because the comparisons available did not differ on class size or child–teacher ratios and therefore could not inform the question of interest.

Bowne et al. (2017) were able to extract 328 effect sizes; 270 effect sizes (within 50 contrasts) were found for cognitive and achievement outcomes and 58 effect sizes (within 20 contrasts) were found for socio‐emotional and behavioural outcomes. Of the 53 contrasts included in the study, three only included socio‐emotional effect sizes, 33 only included cognitive and achievement effect sizes, and 17 included outcomes in both domains.

Results of the meta‐analysis suggest a nonlinear relationship between adult/child ratio and group size and child outcomes with a stronger association at the lower end of the slope, indicating that reductions in adult/child ratio and group size in the lower end of the slope (such as a reduction of one child from a ratio 1:7 rather than a reduction of one child from a ratio of 1:10) are more strongly associated with positive child outcomes.

For cognitive and achievement outcomes, child–teacher ratio and class size were associated with more positive outcomes for children at the lower end of the distribution of class size and child–teacher ratio; that is, only very low child–teacher ratios (7.5:1 and lower) or very small class sizes (15 or less) were associated with significant, although not large, differences for children's cognitive and achievement outcomes. Small changes in class size or ratio (the reduction by one child) in very small, well‐staffed classrooms (i.e., 15 children and two teachers) were only associated with small effect sizes (0.22 and 0.10, respectively). For socio‐emotional outcomes, there was the suggestion that very small classes, but not child–teacher ratios, might be important, but the authors also state that their sample was too small to warrant confidence in the conclusions for socio‐emotional outcomes.

These results may be used to generate hypotheses for future research and may tentatively be used to explain the relatively small effect size for process quality and the insignificant results for language and literacy outcomes from the present review, as the limited number of studies, which could be used in our meta‐analyses, did not allow us to distinguish between changes in group size and adult/child ratio at the higher and lower end of the slope.

## 7 AUTHORS' CONCLUSIONS

### 7.1 Implications for practice

Findings from the present review tentatively support the theoretical hypothesis that reducing adult/child ratio and group size increases process quality in ECEC. This hypothesis is reflected in the existence of standards and regulation on the minimum requirements regarding adult/child ratios and limits on the maximum group size in ECEC. However, our results for process quality were not robust across specifications, and our findings regarding the effect on language and literature were inconclusive.

The present review sought to explore the causal effects of reductions in adult/child ratio and group size in ECEC, and thus did not include qualitative or correlational studies. However, in a systematic review of qualitative research on the impact of adult/child ratio and group size in Scandinavian ECEC, based on 12 studies using interviews with caregivers and observations in ECEC, it can be concluded, that there is remarkable consistency in the findings supporting the theoretical assumptions. From the perspective of staff in ECEC and based on independent observations in ECEC, reducing the adult/child ratio and group size is associated with increased process quality, more developmentally appropriate and stimulating activities for children, better opportunities for forming a closer bond between staff and each child in their care, whereas larger group sizes and more children per adult is perceived to be associated with reduced process quality such as fewer positive adult/child interactions and with a lower job satisfaction for the staff. In the 12 studies only one observational study noted that in some cases reducing the group size may have some negative consequences for children, as it limits the child's choice of play mates. No studies suggested any negative effects of reducing the adult/child ratio. However, despite considerable variation in the adult/child ratio and group size in the ECEC settings studied in the included studies, almost all the caregivers interviewed seemed to consider a reduction from the one they currently worked with to be ideal Dalgaard 2022.

Based on the findings from the present review and the review of qualitative studies, in can be concluded that the research literature to date provides little guidance on what the specific appropriate adult/child ratios and group sizes are, but findings suggest that reducing adult/child ratio and group size is perceived as being beneficial from the perspective of caregivers and independent observers, which is tentatively supported by findings from the present meta‐analyses using process quality outcomes.

### 7.2 Implications for research

The main finding of the present review is that there are surprisingly few quantitative studies exploring the effects of changes to adult/child ratio and group size in ECEC on measures of process quality and on child outcomes. This finding may be seen as a testimony to the urgent need for more contemporary high‐quality research exploring the effects of adult/child ratio and group size in ECEC on measures of process quality and on child socio‐emotional outcomes. Future research exploring the effects of changes to adult/child ratio and group size for children younger than 2 years of age is especially needed. Generally, there is a need for study designs to focus on strengthening the ecological validity of studies meaning that interventions should take place in the naturally occurring everyday life of children and last for a longer period in order for the children to adjust. Very short interventions are unlikely to capture the full range of potential effects and there is a strong risk that children/caregivers behave differently if they are aware that they are being observed for a very short period (e.g., there is a strong risk of experimenter bias). The present data did not include any study measuring outcomes at time points past the end of the intervention, and thus there is no evidence regarding the long term effects of changes to adult/child ratio and group size in ECEC. This should also be explored in future longitudinal studies.

## CONTRIBUTIONS OF AUTHORS

- Content:

Nina T. Dalgaard is a psychologist, Ph.D. Nina has previously worked as both an educational psychologist within a primary school setting and as a clinical child psychologist and thus has knowledge about the socio‐emotional and cognitive development of children.

Anja Bondebjerg holds a Master's degree in Sociology and has worked extensively with systematic reviews and research mappings in the fields of education and early childhood education and care. She is knowledgeable regarding the structure and process of conducting systematic reviews

Rasmus Klokker is M.Sc. in Sociology, has worked on systematic reviews mapping research on daycare and preschool in the Nordic countries, and has general knowledge of the field of sociology of education. Rasmus Klokker is thus knowledgeable on the scholarly literature concerning daycare and preschool, and has general knowledge of educational institutions within a sociological framework. Rasmus has worked on and assisted the completion of several systematic reviews within the Campbell framework. Rasmus Klokker has been involved in all facets of conducting systematic reviews, and has completed a course, lead by Michael Borenstein, on meta‐analysis.

- Systematic review methods:

Jens Dietrichson holds a Ph.d. in economics and is an experienced systematic reviewer and methodologist, having completed a number of systematic reviews as well as primary studies in the fields of education and early childhood education and care. He is currently the lead reviewer on one ongoing Campbell Systematic Reviews and is knowledgeable regarding all major facets of meta‐analytic methods and their application.

Anja Bondebjerg (please see description above)

- Statistical analysis:
  Jens Dietrichson (please see description above)
  Rasmus Klokker (please see description above)
- Information retrieval:

Bjørn C. A. Viinholt (information specialist), holds a master in library and information science and has 4 years of experience in developing and writing systematic reviews. As a part of undertaking systematic reviews, Bjørn has experience in developing systematic search strategies and processes of reference management. Bjørn will contribute with assisting and development of the systematic search strategy, executing the searches, and assist with reference management and grey literature searches. Bjørn will also assist with aspects relating to systematic literature searches in Campbell review methodology.

## DECLARATIONS OF INTEREST

We do not have any potential conflicts of interest.

## DIFFERENCES BETWEEN PROTOCOL AND REVIEW

In the protocol, we stated that we would only extract outcomes if they had been validated on other samples than the intervention sample (researcher observations, caregiver or parental ratings). However, due to the very limited number of included studies within this review, we decided to include measures, which to our knowledge had not been validated on other samples, if they were deemed high in face validity and had a measure of interrater reliability. Examples of measures with a high face validity would be an observation schedule describing very concrete child and adult behaviours such as ‘crying’, ‘aimless wandering’, and ‘adult uses praise’. This was the case with Russell (1990), Smith et al. (1988), and Smith and Connolly (1986) in which the authors state that the observation schedules were designed specifically for their studies.

In our primary analysis, we estimated the effects separately by two conceptual outcomes: process quality and child learning. Based on the protocol, we also aimed to estimate the effects separately by intervention type (changes to adult/child ratio, group size, or both); however, due to the small number of studies, which could be used in the data synthesis, this was not possible. Similarly, we were also unable to conduct separate analyses based on the size of the changes to adult/child ratio and group size.

Furthermore, the protocol for this review stated that we would include both study design and estimation method as moderators in a meta‐regression model. Due to the small number of included studies, this sensitivity check was unfeasible however.

We did not search Medline, even though it was listed in the protocol as a database that would be searched for this review. Medline should have been removed from the list during the revision process of the protocol, since our pilot searches did not identify any unique relevant references.

Furthermore, a few modifications to the search facet structure were implemented in the database searches that differ from the exemplified search string in the protocol. The search facets in the final searches were structured with a clearer distinction between the individual facets/aspects, and we removed some proximity operators to be less restrictive in the searches. This resulted in searches with a higher sensitivity than originally intended.

In the published protocol we did not specify that references and studies were only screened and included if they were published in a language which at least one member of the review team was able to read, for example, Danish, Swedish, Norwegian, German and English. This information has now been added to the methods section.

## PUBLISHED NOTES

**Characteristics of studies**

**Characteristics of included studies**

Allhusen 1991

**Table:** 

Methods	QES (assignment clustered by state), non‐randomised study, daycare centres in two neighbouring states with different requirements regarding adult/child ratios in daycare centres were selected from comparable middle income neighbourhoods to reflect two different ratio conditions: 1:4 versus 1:7.
Participants	Participants were 32 infants (20 girls) and a caregiver from their daycare classroom
Interventions	Ratio: 1:4 and 1:7 (p. 7); Group size is not explicitly mentioned, but if both daycare centres have only one group, both T and C are in groups of 16 (p. 7). They also compare small (<14) and large (**≥**14) groups, but this comparison seems to be across the T and C daycares.
Outcomes	Child‐Rearing Scales, Attachment Q‐set, and Caregiving Effectiveness Scale.
Notes	

Risk of bias table

**Table:** 

Bias	Authors' judgement	Support for judgement
Random sequence generation (selection bias)	Unclear risk	
Allocation concealment (selection bias)	Unclear risk	
Blinding of participants and personnel (performance bias)	Unclear risk	
Blinding of outcome assessment (detection bias)	Unclear risk	
Incomplete outcome data (attrition bias)	Unclear risk	
Selective reporting (reporting bias)	Unclear risk	
Other bias	Unclear risk	

Asher 1979

**Table:** 

Methods	'naturalistic, nonexperimental investigation’ (p. 69)
	14 of the relatively high‐ratio centres in the sample were provided the means to increase their staff and decrease their ratio (p.62)
Participants	14 daycare centres. Control group 35 daycare centres (p. 61)
	700 children out of 1200 observed (p. 99)
Interventions	Unclear how many teachers were added.
Outcomes	26 behaviours from the Prescott Child Focus Inventory (p. 64, Appendix B).
	Behaviours were compiled into 11 factors (p. 77).
Notes	

Risk of bias table

**Table:** 

Bias	Authors' judgement	Support for judgement
Random sequence generation (selection bias)	Unclear risk	
Allocation concealment (selection bias)	Unclear risk	
Blinding of participants and personnel (performance bias)	Unclear risk	
Blinding of outcome assessment (detection bias)	Unclear risk	
Incomplete outcome data (attrition bias)	Unclear risk	
Selective reporting (reporting bias)	Unclear risk	
Other bias	Unclear risk	

Asher, 1979a

**Table:** 

Methods	An experimental design with 16 children observed in three different experimenter‐controlled ratios and two group sizes at one ratio level. The children were observed through 16 64‐min observation sessions during midmorning free play.
Participants	*N* = 16 children (p. 518)
Interventions	Ratios (4:1, 8:1, and 12:1) and two group sizes at one ratio level (8:1 and 16:2)
Outcomes	Independent observation, 10 child and ten teacher variables were recorded, measuring vocalising, touching positively, play etc.
Notes	

Risk of bias table

**Table:** 

Bias	Authors' judgement	Support for judgement
Random sequence generation (selection bias)	Unclear risk	
Allocation concealment (selection bias)	Unclear risk	
Blinding of participants and personnel (performance bias)	Unclear risk	
Blinding of outcome assessment (detection bias)	Unclear risk	
Incomplete outcome data (attrition bias)	Unclear risk	
Selective reporting (reporting bias)	Unclear risk	
Other bias	Unclear risk	

Bleses 2018

**Table:** 

Methods	Cluster‐randomised trial
Participants	*N* = 5436 3–6‐year‐old Danish children from 154 daycare centres in 8 municipalities
Interventions	Three variations of a language‐literacy focused curriculum (LEAP). LEAP LARGE and SMALL involved educators implementation of 40 scripted 30 min lessons per week to small groups or entire classes. LEAP OPEN: the educators were not provided soft‐scripted lessons to use within the 40 lessons: they received the scope and sequence instruction, but had the autonomy to decide which learning domains they would address in each lesson.
Outcomes	The Language Assessment of Children: 3–6 instrument (Bleses et al., 2010)
Notes	

Risk of bias table

**Table:** 

Bias	Authors' judgement	Support for judgement
Random sequence generation (selection bias)	Unclear risk	
Allocation concealment (selection bias)	Unclear risk	
Blinding of participants and personnel (performance bias)	Unclear risk	
Blinding of outcome assessment (detection bias)	Unclear risk	
Incomplete outcome data (attrition bias)	Unclear risk	
Selective reporting (reporting bias)	Unclear risk	
Other bias	Unclear risk	

Brownell 1973

**Table:** 

Methods	Single group, repeated measures design
Participants	*N* = 56 (p. 314)
Interventions	Communications patterns were measured in three conditions all with one teacher: dyad 1:1 (no peers), triad 1:2 (one peer), role‐playing triad 1:2 (one peer) and the small group 1:3 (two peers).
Outcomes	The outcomes are mean length of verbalisation and mean length of verbalisation minus repetitions, details on p. 312.
Notes	

Risk of bias table

**Table:** 

Bias	Authors' judgement	Support for judgement
Random sequence generation (selection bias)	Unclear risk	
Allocation concealment (selection bias)	Unclear risk	
Blinding of participants and personnel (performance bias)	Unclear risk	
Blinding of outcome assessment (detection bias)	Unclear risk	
Incomplete outcome data (attrition bias)	Unclear risk	
Selective reporting (reporting bias)	Unclear risk	
Other bias	Unclear risk	

Cederblad 1980

**Table:** 

Methods	QES. 10 kindergartens were examined to study the effect of different ratios.
Participants	100 children (47 boys and 53 girls). The youngest child was 2,5 years old and the oldest 4.5 years old
Interventions	Half of the kindergartens received an extra caregiver in the first 9 weeks. After the 9 weeks the extra caregivers were re‐located to the remaining kindergartens.
Outcomes	Observations by a child‐psychologist and project‐assistants and descriptions of stress‐factors in the child's life from the parents. In the beginning, middle and end of the study both parents and caretakers are interviewed about the kids' behaviour and well‐being. Two urine samples per child were collected everyday.
Notes	

Risk of bias table

**Table:** 

Bias	Authors' judgement	Support for judgement
Random sequence generation (selection bias)	Unclear risk	
Allocation concealment (selection bias)	Unclear risk	
Blinding of participants and personnel (performance bias)	Unclear risk	
Blinding of outcome assessment (detection bias)	Unclear risk	
Incomplete outcome data (attrition bias)	Unclear risk	
Selective reporting (reporting bias)	Unclear risk	
Other bias	Unclear risk	

De Schipper 2006

**Table:** 

Methods	Single group, repeated measures design. An experimental study where the child‐caregiver ratio is being manipulated (manipulating the number of children assigned to the same caregiver during two play episodes in the same classroom) (p. 863). The children are randomly allocated from the caregiver's usual group and caregivers are assigned randomly to different orders. The play episodes was first examined by correlations between caregiver behaviour (during the structured play episodes) and during mere natural settings (lunch time) (p. 864). Examined the effect of child‐caregiver ratio on caregiver‐child interactions and how it affects child well‐being etc. – also investigating how the effect is different on levels of child age (interaction)
Participants	64 daycare centres. 217 female caregivers.
Interventions	Groups: Ratios of 3:1 and 5:1 experimentally manipulated the number of children assigned to the same caregiver during two play episodes in the same classroom (p. 863). The caregiver‐child interactions were among other variables controlled for group size.
Outcomes	The Caregiver Interaction Scale (CIS) (p. 864).
Notes	

Risk of bias table

**Table:** 

Bias	Authors' judgement	Support for judgement
Random sequence generation (selection bias)	Unclear risk	
Allocation concealment (selection bias)	Unclear risk	
Blinding of participants and personnel (performance bias)	Unclear risk	
Blinding of outcome assessment (detection bias)	Unclear risk	
Incomplete outcome data (attrition bias)	Unclear risk	
Selective reporting (reporting bias)	Unclear risk	
Other bias	Unclear risk	

Endsley 1976

**Table:** 

Methods	Singe group, repeated measures design.
Participants	32 preeschool children (16 boys, and 16 girls) ranging in age from 3.1 to 6.2 years
Interventions	Three experimental sessions with different adult/child ratios. In each session the children were shown a set of ‘interesting’ materials. The children were shown the materials by their teachers while alone, with one other same‐sex peer and with three other same‐sex peers
Outcomes	children's frequency of asking questions (observational measure)
Notes	

Risk of bias table

**Table:** 

Bias	Authors' judgement	Support for judgement
Random sequence generation (selection bias)	Unclear risk	
Allocation concealment (selection bias)	Unclear risk	
Blinding of participants and personnel (performance bias)	Unclear risk	
Blinding of outcome assessment (detection bias)	Unclear risk	
Incomplete outcome data (attrition bias)	Unclear risk	
Selective reporting (reporting bias)	Unclear risk	
Other bias	Unclear risk	

Field 1980

**Table:** 

Methods	QES. The subjects were 80 out of 96 children who had been randomly assigned by the university to four different preschool classrooms used as teacher training facilities. Twenty children were selected for the study from each classroom.
Participants	*n* = 80 (20 children in each classroom).
Interventions	Four different preschool classrooms used as teacher training facilities. The classrooms varied on dimensions of teacher/child ratio and organisation of space, p. 193. Classroom A: a partitioned space and classroom B an open space, both featuring low child/teacher ratios (1/12). Classroom C was a partitioned space and classroom D an open space, both featuring a high teacher/child ratio (1/4).
Outcomes	Parten's play behaviours + interactions
Notes	

Risk of bias table

**Table:** 

Bias	Authors' judgement	Support for judgement
Random sequence generation (selection bias)	Unclear risk	
Allocation concealment (selection bias)	Unclear risk	
Blinding of participants and personnel (performance bias)	Unclear risk	
Blinding of outcome assessment (detection bias)	Unclear risk	
Incomplete outcome data (attrition bias)	Unclear risk	
Selective reporting (reporting bias)	Unclear risk	
Other bias	Unclear risk	

Francis, 2014

**Table:** 

Methods	RCT.
Participants	188 student were in reduced class sizes (39 AM;149 PM) and 226 in regular class sizes (184 AM; 42 PM) (p. 66).
Interventions	Class size is reduced for one session for each teacher participating and was randomly assigned to AM and PM sessions (p. 65)
Outcomes	Peabody Picture Vocabulary Test ‐ Third edition (PPVT‐III), Test of Preschool Emergent Literacy (TOPEL), Woodstock‐Johnson Psycho‐Educational Battery‐Third Edition (WJ‐III), Classroom Assessment Scoring System (CLASS), and coding with Emergent Academics Snapshot
Notes	

Risk of bias table

**Table:** 

Bias	Authors' judgement	Support for judgement
Random sequence generation (selection bias)	Unclear risk	
Allocation concealment (selection bias)	Unclear risk	
Blinding of participants and personnel (performance bias)	Unclear risk	
Blinding of outcome assessment (detection bias)	Unclear risk	
Incomplete outcome data (attrition bias)	Unclear risk	
Selective reporting (reporting bias)	Unclear risk	
Other bias	Unclear risk	

Francis 2019

**Table:** 

Methods	RCT.
Participants	188 student were in reduced class sizes (39 AM;149 PM) and 226 in regular class sizes (184 AM; 42 PM) (p. 66).
Interventions	Class size is reduced for one session for each teacher participating and was randomly assigned to AM and PM sessions (p. 65)
Outcomes	Peabody Picture Vocabulary Test ‐ Third edition (PPVT‐III), Test of Preschool Emergent Literacy (TOPEL), Woodstock‐Johnson Psycho‐Educational Battery‐Third Edition (WJ‐III), Classroom Assessment Scoring System (CLASS), and coding with Emergent Academics Snapshot
Notes	Same data as Francis, 2014

Risk of bias table

**Table:** 

Bias	Authors' judgement	Support for judgement
Random sequence generation (selection bias)	Unclear risk	
Allocation concealment (selection bias)	Unclear risk	
Blinding of participants and personnel (performance bias)	Unclear risk	
Blinding of outcome assessment (detection bias)	Unclear risk	
Incomplete outcome data (attrition bias)	Unclear risk	
Selective reporting (reporting bias)	Unclear risk	
Other bias	Unclear risk	

Howes 1992

**Table:** 

Methods	QES. Quality measures were compared in childcare centres in two states with different standards for adult:child ratios.
Participants	*N* = 414 children
Interventions	Californian standard for adult/child ratio was 1:8 and the Georgian standard was 1:9
Outcomes	ECERS and ITERS (p. 452)
Notes	

Risk of bias table

**Table:** 

Bias	Authors' judgement	Support for judgement
Random sequence generation (selection bias)	Unclear risk	
Allocation concealment (selection bias)	Unclear risk	
Blinding of participants and personnel (performance bias)	Unclear risk	
Blinding of outcome assessment (detection bias)	Unclear risk	
Incomplete outcome data (attrition bias)	Unclear risk	
Selective reporting (reporting bias)	Unclear risk	
Other bias	Unclear risk	

Kim 2001

**Table:** 

Methods	QES. Three classes each of size 30 and 40, and one class of size 20 were compared. Ten children in each class were observed.
Participants	A total of 70 children and seven head teachers in seven different preschools were subjects (p. 92).
Interventions	Four different children‐teacher ratios: 15:1, 20:1 (1 teacher for 20 children and 2 teachers for 40 children), 30:1 and 40:1 (p. 95‐96)
Outcomes	Peer nomination sociometric interviews and observation of subject children. First observation: 3 weeks after school started. Second observation: more than 3 weeks before the closing day (p. 93)
Notes	

Risk of bias table

**Table:** 

Bias	Authors' judgement	Support for judgement
Random sequence generation (selection bias)	Unclear risk	
Allocation concealment (selection bias)	Unclear risk	
Blinding of participants and personnel (performance bias)	Unclear risk	
Blinding of outcome assessment (detection bias)	Unclear risk	
Incomplete outcome data (attrition bias)	Unclear risk	
Selective reporting (reporting bias)	Unclear risk	
Other bias	Unclear risk	

Love 1993

**Table:** 

Methods	Cluster randomised trial. In 1990 trained observers spend a week in 122 classrooms throughout the state. A couple of months later the classrooms was randomly assigned to a new child‐staff ratio configuration.
Participants	122 classrooms
Interventions	One‐third of the classrooms increased their ratio to 9:1, one‐third went to 10:1 and the other third maintained a ratio of 8:1.
Outcomes	Six observational instruments to provide data on classroom structure and dynamics, caregiver and children's behaviour. They include measures of class‐size, caregiver‐child interactions, ratings of caregiver behaviour or style and measures of child behaviour.
Notes	

Risk of bias table

**Table:** 

Bias	Authors' judgement	Support for judgement
Random sequence generation (selection bias)	Unclear risk	
Allocation concealment (selection bias)	Unclear risk	
Blinding of participants and personnel (performance bias)	Unclear risk	
Blinding of outcome assessment (detection bias)	Unclear risk	
Incomplete outcome data (attrition bias)	Unclear risk	
Selective reporting (reporting bias)	Unclear risk	
Other bias	Unclear risk	

McCabe 1996

**Table:** 

Methods	Single group. The study is a 2 × 2 × 2 mixed factorial design.
Participants	Participants *n* = 24 (100%) children with developmental disabilities. Along with these 12 playmates with disabilities and 12 playmates without disabilities (p. 334) Developmental pre‐test scores were assessed before the intervention (Table 3, p. 335)
Interventions	Group size (pair vs. quartets) is examined as within‐subject variable and group composition (segregated or integrated) and type of play activity (functional or dramatic) are between‐subjects variables. Thus, in total 8 different play sessions. Each child was videotaped twice, in the two group sizes.
Outcomes	Utterance rate (RATE); mean length of utterance (MLU); different words spoken (DIFF).
Notes	

Risk of bias table

**Table:** 

Bias	Authors' judgement	Support for judgement
Random sequence generation (selection bias)	Unclear risk	
Allocation concealment (selection bias)	Unclear risk	
Blinding of participants and personnel (performance bias)	Unclear risk	
Blinding of outcome assessment (detection bias)	Unclear risk	
Incomplete outcome data (attrition bias)	Unclear risk	
Selective reporting (reporting bias)	Unclear risk	
Other bias	Unclear risk	

McCartney 1997

**Table:** 

Methods	QES. 40 child‐care centres were sampled in 3 states each. Settings were selected and target children were randomly selected within settings
Participants	718 children participated in the study.
Interventions	Child:Teacher ratio data were obtained by observation as a part of the classroom observations of social behaviour (p. 431)
Outcomes	ECERS, ITERS (p. 432).
Notes	

Risk of bias table

**Table:** 

Bias	Authors' judgement	Support for judgement
Random sequence generation (selection bias)	Unclear risk	
Allocation concealment (selection bias)	Unclear risk	
Blinding of participants and personnel (performance bias)	Unclear risk	
Blinding of outcome assessment (detection bias)	Unclear risk	
Incomplete outcome data (attrition bias)	Unclear risk	
Selective reporting (reporting bias)	Unclear risk	
Other bias	Unclear risk	

Neuman 2013

**Table:** 

Methods	Single group, repeated measures design, each child receives instructions on sets of words in a whole‐group and small group condition and serves as his/her own control (p. 593).
Participants	*N* = 108 (p. 600)
Interventions	Ratio 1:4/5 and 1:18, Group size in the small‐group = 4–5 and the whole‐group = 18 (on average). 8 weeks with instruction 10–12 min per day, 4 weeks in a whole‐group and 4 weeks in small‐group (p. 594)
Outcomes	Curriculum‐related word knowledge; Conceptual knowledge; Categories and properties knowledge (p. 596–597)
Notes	

Risk of bias table

**Table:** 

Bias	Authors' judgement	Support for judgement
Random sequence generation (selection bias)	Unclear risk	
Allocation concealment (selection bias)	Unclear risk	
Blinding of participants and personnel (performance bias)	Unclear risk	
Blinding of outcome assessment (detection bias)	Unclear risk	
Incomplete outcome data (attrition bias)	Unclear risk	
Selective reporting (reporting bias)	Unclear risk	
Other bias	Unclear risk	

Palmerus, 1996

**Table:** 

Methods	QES. Adult/child ratio: two caregivers in the same day care centre unit were observed in year one and two. The same caregivers and mostly the same children were observed.
Participants	*N* = 17 children
Interventions	The mean of children per caregiver was 2.2−/+0,8 during (low ratio) the first period and 4.2−/+1.9 (high ratio) during the second period
Outcomes	Observations of verbal interactions between caregiver and children. With a low ratio: 210 min. With a high ratio: 207 min. Observations during three periods of 4 h (morning, mid‐day and afternoon). Scoring: Definitions of ‘monologue, dialogue, turn etc’ (pp. 49‐50).
Notes	

Risk of bias table

**Table:** 

Bias	Authors' judgement	Support for judgement
Random sequence generation (selection bias)	Unclear risk	
Allocation concealment (selection bias)	Unclear risk	
Blinding of participants and personnel (performance bias)	Unclear risk	
Blinding of outcome assessment (detection bias)	Unclear risk	
Incomplete outcome data (attrition bias)	Unclear risk	
Selective reporting (reporting bias)	Unclear risk	
Other bias	Unclear risk	

Pellegrino 1990

**Table:** 

Methods	QES. 5 teachers at a day‐care centre were observed during play with children using toys. The teachers were observed during 6 different sessions.
Participants	14 children participated—7 younger (1 year) and 7 older (2.5 years).
Interventions	Three different ratio conditions: 1:1, 1:3 and 1:7
Outcomes	The adults' recorded speech was transcribed (p. 103). For the functional analysis, utterances were classified in 4 categories: (a) empathetic behaviour, (b) conversational behaviour, (c) didactic behaviour, (d) organisational behaviour (p. 104).
Notes	

Risk of bias table

**Table:** 

Bias	Authors' judgement	Support for judgement
Random sequence generation (selection bias)	Unclear risk	
Allocation concealment (selection bias)	Unclear risk	
Blinding of participants and personnel (performance bias)	Unclear risk	
Blinding of outcome assessment (detection bias)	Unclear risk	
Incomplete outcome data (attrition bias)	Unclear risk	
Selective reporting (reporting bias)	Unclear risk	
Other bias	Unclear risk	

Pessanha 2017

**Table:** 

Methods	QES. Infant childcare classrooms from the greater metropolitan area of Porto, Portugal, participated in this study. Each classroom was observed twice (6‐month interval between Time 1 and Time 2)
Participants	90 infant childcare classrooms from the greater metropolitan area of Porto, Portugal, participated in this study. Each classroom was observed twice (6‐month interval between Time 1 and Time 2)
Interventions	From Time 1 to Time 2 the infant:adult ratio and group size increased. For time one the average group size was 6.44, and the infant/adult ratio was 1: 2.65, at T2 the average group size was 8.76 and the infant/adult ratio was 1: 3.57 (p. 91).
Outcomes	The Infant/Toddler Environment Rating Scale—Revised (ITERS‐R; Harms et al., 2006), the Classroom Assessment Scoring System—Infant (CLASS‐Infant; Hamre et al., 2014), and the Caregiver Interaction Scale (CIS; Arnett, 1989). Additionally, teachers provided demographic information about themselves and structural characteristics of the classroom.
Notes	

Risk of bias table

**Table:** 

Bias	Authors' judgement	Support for judgement
Random sequence generation (selection bias)	Unclear risk	
Allocation concealment (selection bias)	Unclear risk	
Blinding of participants and personnel (performance bias)	Unclear risk	
Blinding of outcome assessment (detection bias)	Unclear risk	
Incomplete outcome data (attrition bias)	Unclear risk	
Selective reporting (reporting bias)	Unclear risk	
Other bias	Unclear risk	

Phillips, 1992

**Table:** 

Methods	QES.
Participants	227 child care centres in 5 metropolitan areas were examined.
Interventions	Structural features of staff: child ratio and group size were assessed with classroom observations in which the numbers of adults and children were recorded at regular intervals during a 2‐hour observation period. The observations were averaged to create a ratio and group size score for each age group of children (p. 33). The participating centres where classified by whether they met the provisions (ratio, group size) (p. 34).
Outcomes	ECERS, ITERS and staff interviews
Notes	

Risk of bias table

**Table:** 

Bias	Authors' judgement	Support for judgement
Random sequence generation (selection bias)	Unclear risk	
Allocation concealment (selection bias)	Unclear risk	
Blinding of participants and personnel (performance bias)	Unclear risk	
Blinding of outcome assessment (detection bias)	Unclear risk	
Incomplete outcome data (attrition bias)	Unclear risk	
Selective reporting (reporting bias)	Unclear risk	
Other bias	Unclear risk	

Phillips 2000

**Table:** 

Methods	QES that compares centres in states with different ratio requirements. A representative sample of day care centres in three different states was recruited, the three different locations have different regulation of adult/child ratio and group size (among other things) and thus represent different ratio + group size conditions
Participants	*N* = 87 infant classrooms, N = 104 toddler classrooms, and *N* = 96 preschool classrooms (p. 481).
Interventions	See table 5, p. 485, for ratios and group sizes in the three states and the three types of classrooms. The three different locations have different regulation of adult/child ratio and group size (among other things) and thus represent different ratio + group size conditions, measurement was carried out at one time point in which two observers observed the group size and adult/child ratio during a full day, and an average was computed for each classroom
Outcomes	ITERS; ECERS; Assessment Profile for Early Childhood Programs
Notes	

Risk of bias table

**Table:** 

Bias	Authors' judgement	Support for judgement
Random sequence generation (selection bias)	Unclear risk	
Allocation concealment (selection bias)	Unclear risk	
Blinding of participants and personnel (performance bias)	Unclear risk	
Blinding of outcome assessment (detection bias)	Unclear risk	
Incomplete outcome data (attrition bias)	Unclear risk	
Selective reporting (reporting bias)	Unclear risk	
Other bias	Unclear risk	

Phillips 2003

**Table:** 

Methods	Single group, repeated measures manipulating group size—the number of children present at storybook reading (large group/small group) (p. 458). Collection of data in all settings at the same time but introducing the intervention in only one setting at a time while continuing measures of the other settings (p. 457).
Participants	Fifteen 2‐year‐old children and six teachers in two classrooms participated.
Interventions	Reducing group size (p. 456). Baseline: 4 weeks in Classroom One and 8 weeks in Classroom Two. Large group storybook reading was conducted with all children present. In classroom one: 7‐4 children present (median 6) and classroom two: 8–3 (median 6). After baseline the small storybook reading began and implemented for 10 weeks in classroom one and for 6 weeks in classroom two. Two teachers to approximately half of the children in different parts of the classroom. Classroom one: 5–3 children present (median 3) and classroom two 4–3 (median 3) (p. 458).
Outcomes	Children's comments and questions were coded with Morrow and Smith's (1990) four major categories of verbal participation (p. 459). Teacher speech: coded into Morrow and Smith's (1990) seven categories (p. 459). Non‐verbal participation was coded with Strauss and Corbin's categorisation (1990) (p. 465).
Notes	

Risk of bias table

**Table:** 

Bias	Authors' judgement	Support for judgement
Random sequence generation (selection bias)	Unclear risk	
Allocation concealment (selection bias)	Unclear risk	
Blinding of participants and personnel (performance bias)	Unclear risk	
Blinding of outcome assessment (detection bias)	Unclear risk	
Incomplete outcome data (attrition bias)	Unclear risk	
Selective reporting (reporting bias)	Unclear risk	
Other bias	Unclear risk	

Pierce‐Jones 1968

**Table:** 

Methods	QES, comparing two types of Head start programs with different ratios.
Participants	Sample size: 70 culturally deprived subjects from poverty level income families (p. 63). The group size differs from pre‐test to post‐test for both treatment and control group (p. 70).
Interventions	Intervention: Ratio and type of Head start program. Treatment Group: 39 subjects enroled in Head Start 6 weeks summer program (mothers from the community with a small adult‐child ratio 1 to 4). Control group: 30 subjects enroled in regular Head start program (teachers with a teacher‐student ratio 25 to 1).
Outcomes	Independent observation (observing whether the subject matches the presented card with an appropriate part of own body or doll ‐ afterwards drawing a picture of the subject itself ‐ scoring it by body differentiation) (p. 64).
Notes	

Risk of bias table

**Table:** 

Bias	Authors' judgement	Support for judgement
Random sequence generation (selection bias)	Unclear risk	
Allocation concealment (selection bias)	Unclear risk	
Blinding of participants and personnel (performance bias)	Unclear risk	
Blinding of outcome assessment (detection bias)	Unclear risk	
Incomplete outcome data (attrition bias)	Unclear risk	
Selective reporting (reporting bias)	Unclear risk	
Other bias	Unclear risk	

Russell, 1990

**Table:** 

Methods	QES. The study investigated the effects of small changes in child staff ratio on observed child/staff behaviour in 27 preschools. Numbers of children were manipulated to produce a low ratio (7.7:1), an average ratio (9.2:1) and a high ratio (11.2:1).
Participants	25 children in each of the 27 preschools.
Interventions	Intended ‘normal’ group size = 30. Intended staff/child ratios 1:8, 1:10, and 1:12. Received on average: 1:7,7; 1:9,2; 1:11,2 (p. 78). Intended high and low group sizes not mentioned, but should be 24 (=3 × 8) and 36 (=3 × 12).
Outcomes	Different literature influenced the observation schedule designed for this study (p. 79).
Notes	

Risk of bias table

**Table:** 

Bias	Authors' judgement	Support for judgement
Random sequence generation (selection bias)	Unclear risk	
Allocation concealment (selection bias)	Unclear risk	
Blinding of participants and personnel (performance bias)	Unclear risk	
Blinding of outcome assessment (detection bias)	Unclear risk	
Incomplete outcome data (attrition bias)	Unclear risk	
Selective reporting (reporting bias)	Unclear risk	
Other bias	Unclear risk	

Smith 1980

**Table:** 

Methods	Two experiments. A 49‐centre quasi‐experiment including Atlanta, Detroit and Seattle—Natural experiment: variations in group sizes, ratios and qualifications because of the different local regulatory policies (p. 719). The Atlanta Public School 8‐centre experiment—RCT—experimental manipulation of staff‐child ratio and years of caregiver education. Children randomly assigned to classrooms within the 8 centres (p. 719).
Participants	The study sample included more than 1600 children and 300 caregivers in 150 classrooms (p. 719). 32 day care centres in Atlanta, 16 in Detroit and 16 in Seattle (p. 718).
Interventions	A comparison of three groups: Treatment group 14 low‐ratio centres increasing ratios from an average of 1:9 to 1:6. Two different groups of naturally occurring ratios: A matched group of 14 low‐ratio (1:9) centres and a group consisting of 21 high‐ratio (1:6) centres (p. 719)
Outcomes	Child behaviour was measured with the Child Focus Observation Instrument. The caregiver behaviour was measured with an Adult Focus Observation instrument. The child test scores were in the Preschool Inventory (PSI) and the Peabody Picture Vocabulary Test (PPVT) (p. 719).
Notes	data from: National day care study

Risk of bias table

**Table:** 

Bias	Authors' judgement	Support for judgement
Random sequence generation (selection bias)	Unclear risk	
Allocation concealment (selection bias)	Unclear risk	
Blinding of participants and personnel (performance bias)	Unclear risk	
Blinding of outcome assessment (detection bias)	Unclear risk	
Incomplete outcome data (attrition bias)	Unclear risk	
Selective reporting (reporting bias)	Unclear risk	
Other bias	Unclear risk	

Smith 1986

**Table:** 

Methods	QES. The whole study is 3 years long and contains different interventions/designs. 2 educational play groups of children participated in the study. The interventions containing group size and adult child ratio was ‘The number of children in the playgroup’ (p. 38–42) and ‘Effects of varying staff‐child ratio’ (pp. 54–59).
Participants	*N* = 24 children
Interventions	In the ‘Effects of varying staff‐child ratio’ study the class size ratios were 2:3 and 1:2 respectively during 2 terms. Given the number of children present (Table 2), the actual staff‐child ratio varied from 1:4 in the ‘best’ conditions and 1:14 in the ‘worst’ (p. 55)
Outcomes	Observations during focal‐staff samples (pp. 31, 55) of different behaviour categories (p. 31).
Notes	

Risk of bias table

**Table:** 

Bias	Authors' judgement	Support for judgement
Random sequence generation (selection bias)	Unclear risk	
Allocation concealment (selection bias)	Unclear risk	
Blinding of participants and personnel (performance bias)	Unclear risk	
Blinding of outcome assessment (detection bias)	Unclear risk	
Incomplete outcome data (attrition bias)	Unclear risk	
Selective reporting (reporting bias)	Unclear risk	
Other bias	Unclear risk	

Smith 1988

**Table:** 

Methods	Four experimental and four control Kindergartens were selected for participation in two locations. Experimental Kindergartens hired a third Kindergarten teacher while control Kindergartens continued with their usual staffing of two teachers. 35 children and 13 teachers were observed across all three data collection sessions
Participants	48 children at baseline (24 intervention, 24 control), but 35 (17 Boys, 18 girls) in the final data collection (intervention + control group membership not reported)
Interventions	Adult/child ratio in treatment and control group. At baseline all eight kindergartens had two teachers (ratio 1:20). Four kindergartens received a third teacher after the first observation (ratio 1:13.33). This is the ITT ratio (observed was different, see Table 3, p. 134).
Outcomes	Child observations; Teacher observations; Parent questionnaires. Independent observation+ parental questionnaires and teacher interviews
Notes	

Risk of bias table

**Table:** 

Bias	Authors' judgement	Support for judgement
Random sequence generation (selection bias)	Unclear risk	
Allocation concealment (selection bias)	Unclear risk	
Blinding of participants and personnel (performance bias)	Unclear risk	
Blinding of outcome assessment (detection bias)	Unclear risk	
Incomplete outcome data (attrition bias)	Unclear risk	
Selective reporting (reporting bias)	Unclear risk	
Other bias	Unclear risk	

Smith 1988a

**Table:** 

Methods	Four experimental and four control Kindergartens were selected for participation in two locations. Experimental Kindergartens hired a third Kindergarten teacher while control Kindergartens continued with their usual staffing of two teachers. 35 children and 13 teachers were observed across all three data collection sessions
Participants	48 children at baseline (24 intervention, 24 control), but 35 (17 Boys, 18 girls) in the final data collection (intervention + control group membership not reported)
Interventions	Adult/child ratio in treatment and control group. At baseline all eight kindergartens had two teachers (ratio 1:20). Four kindergartens received a third teacher after the first observation (ratio 1:13.33). This is the ITT ratio (observed was different, see Table 3, p. 134).
Outcomes	Child observations; Teacher observations; Parent questionnaires. Independent observation + parental questionnaires and teacher interviews
Notes	

Risk of bias table

**Table:** 

Bias	Authors' judgement	Support for judgement
Random sequence generation (selection bias)	Unclear risk	
Allocation concealment (selection bias)	Unclear risk	
Blinding of participants and personnel (performance bias)	Unclear risk	
Blinding of outcome assessment (detection bias)	Unclear risk	
Incomplete outcome data (attrition bias)	Unclear risk	
Selective reporting (reporting bias)	Unclear risk	
Other bias	Unclear risk	

Travers 1980

**Table:** 

Methods	QES. 49‐centres across three study sites (Atlanta, Detroit, and Seattle) which compared three groups of centres (treatment, untreated low‐ratio and untreated high‐ratio).
Participants	At the beginning of Phase 3, approximately 1600 three‐ and four‐year‐old children were enroled in the 57 study centres (QES and randomised trial combined). About 300 staff were employed as teachers or aides (from Children at the Centre). From the report: 210 caregivers were observed in the fall and 220 in the Spring. In the Fall, 1310 children were observed, and 1108 in the Spring.
Interventions	Treatment group ratio 1:5,9. Comparison groups: untreated low‐ratio centres (1:9,1), and high‐ratio centres (1:5,9)
Outcomes	Direct observations by trained observers and gains from Fall to Spring on two standardised tests: The Preschool Inventory and the Peabody Picture Vocabulary Test
Notes	Research results of the National Day Care Study

Risk of bias table

**Table:** 

Bias	Authors' judgement	Support for judgement
Random sequence generation (selection bias)	Unclear risk	
Allocation concealment (selection bias)	Unclear risk	
Blinding of participants and personnel (performance bias)	Unclear risk	
Blinding of outcome assessment (detection bias)	Unclear risk	
Incomplete outcome data (attrition bias)	Unclear risk	
Selective reporting (reporting bias)	Unclear risk	
Other bias	Unclear risk	

Travers 1982

**Table:** 

Methods	Randomised experiment conducted in 8 centres (29 classrooms) in Atlanta. Children were randomly assigned, within centres, to classrooms which differed systematically in level of staff education (high/medium/low) and ratio (high vs. low).
Participants	At the beginning of Phase 3, approximately 1600 three‐ and four‐year‐old children were enroled in the 57 study centres (QES and randomised trial combined). About 300 staff were employed as teachers or aides (from Children at the Centre). From the report: 210 caregivers were observed in the fall and 220 in the Spring. In the Fall, 1310 children were observed, and 1108 in the Spring.
Interventions	high ratio: 1:5.4, and low ratio: 1:7.4
Outcomes	Child‐Focus Instrument, Adult‐Focus Instrument, Preschool Inventory, and Peabody Picture Vocabulary Test
Notes	Research results of the National Day Care Study

Risk of bias table

**Table:** 

Bias	Authors' judgement	Support for judgement
Random sequence generation (selection bias)	Unclear risk	
Allocation concealment (selection bias)	Unclear risk	
Blinding of participants and personnel (performance bias)	Unclear risk	
Blinding of outcome assessment (detection bias)	Unclear risk	
Incomplete outcome data (attrition bias)	Unclear risk	
Selective reporting (reporting bias)	Unclear risk	
Other bias	Unclear risk	

**Characteristics of excluded studies**

**Table:** 

Bauchmüller 2014	
Reason for exclusion	Wrong study design (Cohort)
Cassidy 1977	
Reason for exclusion	Wrong setting, children too old
Gay 2018	
Reason for exclusion	Wrong study design (Cohort)
Goelman 2000	
Reason for exclusion	Wrong study design (correlational)
Holloway and Reichhart‐Erickson (1988)	
Reason for exclusion	Wrong study design (correlational)
Lera 1996	
Reason for exclusion	Wrong study design (correlational)
Maligalig 2010	
Reason for exclusion	Wrong setting, children too old
Wolf 2019	
Reason for exclusion	Qualitative Study

## ADDITIONAL TABLE

**1 Number of participants by study**

**Table:** 

Study	Condition	Assignment level	Test level	outcome category	*N* control	*N* Intervention	N Total
Travers, J. et al. (1980)	Treatment versus Natural low—Lead teachers	group	staff	Process quality	32	33	65
Travers, J. et al. (1980)	Treatment versus Natural low—teacher aides	group	staff	Process quality	13	20	33
Smith A.B., McMillan B. W; Kennedy,S. & Ratcliffe, B. (1988)	Treatment1	kindergarten	Child group	Process quality	14	21	35
Smith A.B., McMillan B. W; Kennedy,S. & Ratcliffe, B. (1988)	Treatment1	kindergarten	teacher	Process quality	7	6	13
Russell, A. (1990)	Treatment 1 (ratio of 7,7 child) vs. control	individual	Child group	Process quality	27	27	54
Russell, A. (1990)	Treatment 1 (ratio of 7,7 child) vs. control	individual	staff	Process quality	27	27	54
Neuman, S. B. & Kaefer, T. (2013)	Treatment 1	classroom	child	academic outcome	108	108	216
Francis, J. & Barnett, W. S. (2019)	Treatment 1	classroom	child	academic outcome	181	161	340
Francis, J. & Barnett, W. S. (2019)	Treatment 1	classroom	classroom	Process quality	22	22	44
de Schipper, E. J. et al. (2006)	Treatment1	Individual/Teacher	Child group	Process quality	217	217	434
de Schipper, E. J. et al. (2006)	Treatment1	Individual/Teacher	Teacher	Process quality	217	217	434
Bleses, D. et al. (2018)	Treatment 1	day care centres	child	academic outcome	1217	1361	2578
Total					2082	2220	4300

**2 Studies by country**

**Table:** 

		Reduction due to			
Country	Total	Cannot calculate effect size	Too high risk of bias	Used same data sets	Used in data synthesis
(South) Australia	1				1
Denmark	1				1
England	1				1
Italy	1	1			0
Korea	1		1		0
New Zealand	2			2	2
Portugal	1		1		0
Sweden	2		2		0
The Netherlands	1				1
USA	20	4	10	7	6
Total	31	5	14	9	12

## SOURCES OF SUPPORT

Internal sources

- VIVE Campbell, Denmark

External sources

- No sources of support provided

## Supporting information

Supporting information.

## Footnotes

^1^

https://www.oecd.org/els/soc/PF3_2_Enrolment_childcare_preschool.pdf

^2^

https://www.boerneraadet.dk/media/30309/Miniboernepanel-Mellem-hjem-og-boernehave.pdf

^3^

https://data.worldbank.org/indicator/SE.PRE.ENRR
