# Supplementary material for: Adult/child ratio and group size in early childhood education or care to promote the development of children aged 0–5 years: A systematic review
Source: Campbell Syst Rev. 2022 May 4;18(2):e1239. doi: 10.1002/cl2.1239 (PMC9066244; doi:10.1002/cl2.1239)
Supplement: Supplementary file 1 — Supporting information. [file CL2-18-e1239-s001.docx]

# Appendices

## 1 Risk of Bias in included studies

## Studies assessed with ROB 2

### Bleses et al. (2018)

| **Item** | **Authors’ judgment** | **Support for judgment** |
| --- | --- | --- |
| Overall study rating | Moderate risk of bias | Overall this is a sound trial, but the problem is that the ecological validity is questionable, as the intervention (LEAP contains different versions) is an intervention to support children's language acquisition and thus differs from the normal everyday life in daycare. |
| Randomization process | Low risk of bias | 156 daycare centres from 8 municipalities were stratified and then randomized to 4 conditions. |
| Deviations from intervention | Low risk of bias | Fidelity to the LEAP interventions was ensured using both independent observation and educator questionnaires. |
| Missing outcome data | Some concerns | 2 daycare centres withdrew after randomization and 9 daycare centres withdrew between pre- and post-tests, furthermore some withdrew due to children's attendance, moving etc. (between 16-21 % in each condition). |
| Measurement of outcome | Low risk of bias | Measures are standardized. |
| Selection of reported results | Moderate risk of bias | No reference to a pre-registered analysis plan. Nothing to suggest reporting bias. |

### Francis, & Barnett (2019)/ Francis (2014)

| **Item** | **Authors’ judgment** | **Support for judgment** |
| --- | --- | --- |
| Overall study rating | High risk of bias | The lack of blinding of participants and testers and the subsequent switch of session time and the resulting imbalance are most problematic. Although blinding would be impossible and it must be questioned whether the imbalances are caused in a systematic way. Perhaps the switching of reduced class from AM to PM had more to do with the children's parents' timetables than with teacher preference. |
| Randomization process | High risk of bias | Teachers’ AM or PM sessions were randomized to be small or large class size. There is no information provided about the method for sequence generation. Allocation sequence is not concealed over time. 6 out of 22 teachers switched their assigned sessions for unclear reasons. |
| Deviations from intervention | High risk of bias | It is unclear if the children knew they were participating in a study and their treatment status, while teachers and parents were aware of treatment status. There were no co-interventions. Six out of 22 teachers switched their assigned sessions for unclear reasons (p. 51) - resulting in a large imbalance between the reduced and regular class sizes in terms of AM/PM sessions. 80.3% in AM sessions in the regular class size group, 21.7% in the reduced class size group (Table 2, p. 51). The analysis does not take the switched assignment status into account. |
| Missing outcome data | Some concerns | 2 out of 44 classrooms were not included in all measurements, due to the lack of returned consent forms (p. 51). Attrition from pre- to post-test is 15% in regular classes and 14% in reduced class size classes (it is not clear how they have calculated the different numbers in table 1, p. 51). No test of differentiel attrition. |
| Measurement of outcome | Some concerns | The measurement of outcomes seem appropriate, and did probably not differ between groups. There is no information about whether testers where blind to treatment status, and it is possible that the assessment could have be affected by knowledge of treatment status but unclear if this was the case. |
| Selection of reported results | Some concerns | There is no reference to a pre-specified analysis plan. The authors select the best score on the English or Spanish version of the PPVT. They mention many analyses but report only one. However, they report that there were no substantial differences between specifications. |

## Studies assessed with ROBINS-I

Allhusen, & Cochran (1991)

| **Bias** | **Authors’ judgment** | **Support for judgment** |
| --- | --- | --- |
| Overall study rating | Critical risk of bias | Critical risk of confounding bias |
| Confounding | Critical risk of bias | The assignment mechanism is that the children are in two different states, which have different ratio requirements. Participants were not able to switch between groups. However, it seems unlikely that the ratio is the only difference between any two states. If so, then the intervention is not just the ratio, but everything else that is different between the two states. No adequate statistics shown on the balance between groups and no pre-tests scores available. Mentioned that "the only difference between ratio groups was the number of changes in daycare arrangements", which "affected the length of time infants had attended the current center" (p. 7). No adjustment for confounders in the analysis we can use (the regression results are reported in figures, which we cannot use, and these analyses do not adjust for all important confounders either). Furthermore, the group size comparison (p. 11) don't take into account the ratio, and the ratio comparison don't take group size into account. Hence, the two are confounded. |
| Selection | No information |  |
| Classification | No information |  |
| Deviation | No information | There is no information regarding potential "co-interventions" for TOT. |
| Missing data | No information |  |
| Measurement | Moderate risk of bias | Observers (outcome assessors could not be blinded to the intervention). |
| Reporting | Moderate risk of bias | There is no reference to a pre-specified analysis plan. No indication of reporting bias, but a lot of information is missing. |

Asher, & Erickson (1979)

| **Bias** | **Authors’ judgment** | **Support for judgment** |
| --- | --- | --- |
| Overall study rating | Critical risk of bias | The outcome measure - recording behavior (such as touching, vocalization, restraining child, bringing oneself physically down to child's level) would in principle be a useable outcome, but vital information is missing. The whole description of the study is lacking basic information. There is no information on how the groups were created and whether child characteristics were balanced across conditions. Several results for group sizes are not reported (only reported as not significant). |
| Confounding | No information | Single-group design. Not much information about how the groups were put together or about whether they were balanced when smaller group sizes were used. |
| Selection | Critical risk of bias | All seems to be followed from start to finish (p. 518), but no information about how the groups were put together. It is uncertain if were all children observed in all conditions |
| Classification | Serious risk of bias | The intervention groups were not clearly defined, the information used to define intervention groups was not recorded at the start of the intervention, but the classification of intervention status was probably not affected by knowledge of the outcome. |
| Deviation | No information |  |
| Missing data | No information |  |
| Measurement | No information |  |
| Reporting | Serious risk of bias | Several results for the group size conditions are only reported as not significant. |

Asher (1979)

| **Bias** | **Authors’ judgment** | **Support for judgment** |
| --- | --- | --- |
| Overall study rating | Critical risk of bias | This is a dissertation based on a part of a larger study. The design of the study is poorly described, N is unclear and even the intervention is only vaguely described making it impossible to figure out if all classes had extra teachers assigned or if only some of the classrooms had additional teachers present and for how long the intervention lasted. |
| Confounding | Critical risk of bias | Not really described and no mentioning of confounders by intervention status. |
| Selection | No information |  |
| Classification | Critical risk of bias | N is unclear and even the intervention is only vaguely described making it impossible to figure out if all classes had extra teachers assigned or if only some of the classrooms had additional teachers present |
| Deviation | No information |  |
| Missing data | Critical risk of bias | Attrition unclear/not reported. |
| Measurement | Moderate risk of bias |  |
| Reporting | Critical risk of bias | This is a ph.d dissertation based on a larger study, and it is unclear how and why data for this dissertation was obtained from the full dataset. |

Brownell, & Smith (1973)

| **Bias** | **Authors’ judgment** | **Support for judgment** |
| --- | --- | --- |
| Overall study rating | Serious risk of bias | It is not certain whether the outcome can be used and another outcome is described, but not reported. The reliability and validity of the measures are unclear and highly problematic, testers are not blind to treatment status. The ecological validity is questionable as this experiment contains a task, which may as well have been performed in a laboratory setting. There is nothing to suggest or justify that this task/ situation is similar to the everyday preschool environment of the participants. Furthermore, the number of words per utterance does not equal language ability as stated in the discussion. Therefore, there is no ground to conclude that more words is positive in the analysis. It is suggested that there is a critical risk of bias. |
| Confounding | Moderate risk of bias | Single-group design. All children participate in all group conditions, therefore, the mean over sessions should be un-confounded by child characteristics, given that the sessions are close in time. Session order randomized across centers (p. 313). Reliability and validity of measure are not discussed, but it is unclear why it would cause confounding. |
| Selection | Low risk of bias | All subjects included in all conditions. There is no information about lack of observations or attrition of subjects, so perhaps none. |
| Classification | Low risk of bias | The intervention groups were clearly defined and the information used to define intervention groups was recorded at the start of the intervention, and the classification of intervention status was not affected by knowledge of the outcome. |
| Deviation | Low risk of bias | No deviations and no co-interventions are reported (if role-playing condition is deemed a co-intervention, this can be excluded from analysis). No information about adherence problem. |
| Missing data | Low risk of bias | No missing data reported. |
| Measurement | Serious risk of bias | Outcomes could have been influenced by knowledge of the received intervention, outcome assessors were aware of the intervention received. Methods of outcome assessment were comparable across groups, and there were probably not any systematic errors in the measurement of the outcome that were related to intervention group. |
| Reporting | Serious risk of bias | The verbal task described in the methods section is not reported in the results section. |

Cederblad et al. (1980)

| **Bias** | **Authors’ judgment** | **Support for judgment** |
| --- | --- | --- |
| Overall study rating | Critical risk of bias | Cross-over design between ratios 1:5 and 1:3. 81 children, 18 weeks. Overall well-designed, but one major issue is the removal from the analysis of four out of ten daycares in which the intended change in ratio was not achieved (meaning that N in the analyses is 59 children). Furthermore, the large variation in achieved ratios is problematic (deviation from intended intervention). |
| Confounding | No information | It is not clear how the authors handle confounders. |
| Selection | Moderate risk of bias | Not entirely clear how departments were selected for participation in the study, but selection is stated to be random. The placement of departments in the cross-over design (which departments start with intervention and which finish) is not entirely well-described. There could potentially be a risk of sequence effects. |
| Classification | Low risk of bias | Nothing suggests classification bias. |
| Deviation | Moderate risk of bias | Relatively large variation in ratios between departments and from day to day. Occurrence of unplanned changes in number of children and staff. Intervention implementation therefore does not seem entirely successful (ratio was not as intended). Four out of ten departments were excluded from the analyses because the intended ratio changes were not achieved. |
| Missing data | Critical risk of bias | Four out of ten departments were excluded from analyses because the intended ratio changes were not achieved. There is a risk of the missing data not being random (systematic attrition, due to day cares not achieving ratio being special in some way). |
| Measurement | Moderate risk of bias | Ratio is measured by using a diary that is filled in each day. Sick days and behaviour are also reported in diary. Behaviour also measured through interviews with staff and parents (here satisfaction is also measured) and through observation. Finally, urine samples are drawn for levels of adrenalin and noradrenalin (stress). Blinding not possible. Relevant, sound measures. |
| Reporting | Critical risk of bias | Four out of ten departments were excluded from analyses because the intended ratio changes were not achieved. This is a large portion of data being removed - authors should show analyses both with and without these data included and account for differences in results. There is a risk that the analysis may be biased. |

de Schipper et al. (2006)

| **Bias** | **Authors’ judgment** | **Support for judgment** |
| --- | --- | --- |
| Overall study rating | Moderate risk of bias | Repeated measures design. An experimental study where the child-caregiver ratio is being manipulated (manipulating the number of children assigned to the same caregiver during two play episodes in the same classroom) (p. 863). The children are randomly allocated from the caregiver's usual group and caregivers are assigned randomly to different orders. The play episodes was first examined by correlations between caregiver behavior (during the structured play episodes) and during mere natural settings (lunch time) (p. 864). Examined the effect of child-caregiver ratio on caregiver-child interactions and how it affects child well-being etc. – also investigating how the effect is different on levels of child age (interaction) |
| Confounding | Low risk of bias | The authors use appropriate methods when controlling for the risk of child age and caregiver experience and education. |
| Selection | Low risk of bias | Nothing to suggest selection bias |
| Classification | Low risk of bias | Nothing to suggest classification bias |
| Deviation | Low risk of bias | Nothing to suggest deviation bias |
| Missing data | Moderate risk of bias | 28 caregivers and 1 male were excluded from the analyses due to incomplete data, no explanations provided. |
| Measurement | Moderate risk of bias | Observers/ assessors were not blind to the intervention as this would be impossible. |
| Reporting | Moderate risk of bias | No a priori plan could be found. But otherwise no indications of selective reporting. |

Endsley, & Gupta (1976)

| **Bias** | **Authors’ judgment** | **Support for judgment** |
| --- | --- | --- |
| Overall study rating | Moderate risk of bias | Single group design with three experimental sessions with different child/teacher ratios. In each session the children were shown a set of "interesting" materials. The children were shown the materials by their teachers while alone (1G), with one other same-sex peer (2G) and with three other same-sex peers(4G). The number of questions asked (by the children) concerning the materials were tested. |
| Confounding | Low risk of bias | Presumably all children participated in each condition |
| Selection | Low risk of bias |  |
| Classification | Low risk of bias |  |
| Deviation | Moderate risk of bias | Not a lot of information regarding co-interventions. |
| Missing data | No information |  |
| Measurement | Moderate risk of bias | Outcome is not based on a standardized measure but has high face value and inter-rater reliability. |
| Reporting | Moderate risk of bias | There is no reference to a pre-specified analysis plan. Nothing to suggest reporting bias. |

Field (1980)

| **Bias** | **Authors’ judgment** | **Support for judgment** |
| --- | --- | --- |
| Overall study rating | Serious risk of bias | We lack information to calculate an effect size for the adult/child-ratio treatment. Serious risk of bias in the confounding and measurement domains. |
| Confounding | Serious risk of bias | Children (3-4 year old) and teachers randomly assigned to four university lab classrooms (teacher training facilities), but risk of non-random selection post-randomization (see Selection bias-domain). No pre-treatment outcomes or characteristics are reported. Possible confounding by teacher effects (personality and teaching style), regardless of the selection issue. |
| Selection | Moderate risk of bias | 80 children, 20 children from each classroom, out of 96 randomly assigned by the university were selected to participate in the study. Selection is based on age and gender, but the specifics are unclear. So there is risk of non-random selection of participants. |
| Classification | Low risk of bias | No indication of classification bias. |
| Deviation | Low risk of bias | Classrooms were organized to vary on teacher/child ratio (low: 1:12, and high: 1:4) and organization of classroom space (large, open vs. partitioned). The effects found on outcome measures (play behavior and interaction) are therefore a combination of space configuration and ratio. This co-intervention was balanced across the high and low ratio groups. |
| Missing data | Low risk of bias | Three children dropped out of the program during the second month and were immediately replaced, and four children dropped out of the program during the eighth month of observation. Since only two observations were missing for the latter children, group means for their sex and classroom were used for their missing data points. |
| Measurement | Serious risk of bias | 9 months observations by graduate students naive to the study design (but not blind). Good interobserver reliabilities. Uses Parten's classification of play (probably standardized or used on other samples) and observation of interaction (unclear if used on other samples). |
| Reporting | Moderate risk of bias | No pre-registered analysis plan, otherwise no indication of selective reporting. |

Howes et al. (1992)

| **Bias** | **Authors’ judgment** | **Support for judgment** |
| --- | --- | --- |
| Overall study rating | Critical risk of bias | Critical risk of bias. This is essentially also a correlational study as the "intervention" is defined in existing data sets from 3 different states, leaving it impossible to determine if intervention/ control groups are comparable on confounders (not reported). |
| Confounding | Critical risk of bias | Confounders not really accounted or controlled for. |
| Selection | Serious risk of bias | "Selection" into the study was not random (e.g. children lived in different states). |
| Classification | Moderate risk of bias | Measurements of adult/child ratio and group size are based on observations (but only for a short period of time) and may in principle vary a lot. |
| Deviation | Moderate risk of bias | There is no way of knowing if adult/child ratio and group size were actually varying a lot in the specific classrooms and if the observed ratios or group sizes were representative of the children's general experience. |
| Missing data | Serious risk of bias | Attrition is not mentioned, no missing data is mentioned and it appears that data from the previous studies was selected based on the fact that it was available. |
| Measurement | Moderate risk of bias | Measures of outcomes: attachment Q-set is validated, social orientation is measured using a method used in another study. |
| Reporting | Moderate risk of bias | No a priori study plan is mentioned, otherwise nothing to suggest reporting bias. |

Kim (2001)

| **Bias** | **Authors’ judgment** | **Support for judgment** |
| --- | --- | --- |
| Overall study rating | Critical risk of bias | This should be critical as there is only one unit of allocation (one class with 20 students) and the adult/child ratio varies within the different conditions. In addition, it is not certain whether these outcomes can be used for the purpose. |
| Confounding | Serious risk of bias | Ratio varies and confounders by intervention group status are not accounted for. |
| Selection | Moderate risk of bias | "Selection" into the study was not random (e.g. children lived in different places). |
| Classification | Moderate risk of bias | The intervention, when defined as group size, may be considered problematic when not based on observation and there is no record of absent children. |
| Deviation | Moderate risk of bias | Fidelity (counting actual group size) is not accounted for. |
| Missing data | Moderate risk of bias | Some children were not present for observation at time 2 (226-201 = attrition= 25). |
| Measurement | Moderate risk of bias | No indication that outcome measures were influenced by knowledge of received intervention. Outcome assessors were not blind to the intervention. For the peer nomination interviews, assessors were most likely not blind to class size status. Methods of outcome assessment were comparable across intervention groups. Seemingly no systematic errors in measurement of the outcome related to the intervention received. Validity of measures uncertain. |
| Reporting | Moderate risk of bias | No a priori analysis plan mentioned, but otherwise nothing to suggest reporting bias. |

Love (1993)

| **Bias** | **Authors’ judgment** | **Support for judgment** |
| --- | --- | --- |
| Overall study rating | Unclear | This is a conference paper presentation and a lot of information is missing. The full technical report was not accessible. However, the study was funded by the authorities, who have a strong economic incentive favoring a finding of no negative outcomes of lower ratios. |
| Confounding | Moderate risk of bias | Moderators are not shown by intervention group. |
| Selection | Low risk of bias | Nothing to suggest selection bias |
| Classification | Low risk of bias | Nothing to suggest classification bias |
| Deviation | Moderate risk of bias | Observed ratios differed from the specified intervention, no measure of fidelity. |
| Missing data | Moderate risk of bias | Attrition: 10 out of 122 class rooms. |
| Measurement | Low risk of bias | Nothing to suggest measurement bias |
| Reporting | Serious risk of bias | A lot of information is missing |

McCabe et al. (1996)

| **Bias** | **Authors’ judgment** | **Support for judgment** |
| --- | --- | --- |
| Overall study rating | Moderate risk of bias | Experiment 1 is not relevant for our purposes, as it does not manipulate either group size or ratio. Some risk of bias in confounding, deviation, and measurement domains in Experiment 2. |
| Confounding | Moderate risk of bias | Experiment 2 uses a single-group design to examine group size, where the order "in which a child participated in the two- and four-person play sessions was counterbalanced." (p. 335). As children are compared to themselves, baseline characteristics are balanced by design. Play sessions are videotaped one week apart, so low risk of confounding the effects with developmental trends. There are adults present but no information about whether the number of adults differ between sessions. |
| Selection | Low risk of bias | Single-group design, so the same children are observed in both group sizes and are followed throughout the intervention. |
| Classification | Low risk of bias | No indication of classification bias. |
| Deviation | Moderate risk of bias | There are two co-interventions: type of group (segregated or integrated), and type of play (functional or dramatic). Children seem to be randomly assigned to these conditions, they are not counter-balanced (i.e., children only participate in one out of four conditions/combinations of group type and play type, once in a large group and once in a small group) but with an equal number of children in each condition. The group size effect should not be biased by these interventions, as the single-group design compares children to themselves and averages across the other two conditions. There is no mention of implementation problems or of children switching groups. |
| Missing data | Low risk of bias | No missing data reported. |
| Measurement | Moderate risk of bias | Measure language use/opportunity to use language by counting the frequency of utterances, measure the mean length of utterances, and the number of different words spoken using videos. Transcribe the videotapes using the Codes for the Human Analysis of Transcripts (CHAT) system, a standard transcription system. One third of the sessions was double-coded, which showed high correlations with original transcripts (> 0.91). There is one outlier that does not seem to significantly alter the results. Perhaps more problematic that they are omitting all utterances adressed to adults and routines such as counting, reciting, or singing. If these types of utterances are more frequent in one of the conditions, they take up time from other utterances, and may bias the measured effects. |
| Reporting | Moderate risk of bias | No pre-registered analysis plan found. No indication of selective reporting. |

McCartney et al. (1997)

| **Bias** | **Authors’ judgment** | **Support for judgment** |
| --- | --- | --- |
| Overall study rating | Critical risk of bias | This is a correlational design in which four different types of child care (Profit chain, profit local, Non-profit and church sponsored) in 3 different states (Massachusetts, Virginia & Georgia) are compared (there are separate analyses for infant, toddler and preschool rooms). The states have different regulations regarding adult/child ratio, but a lot of other parameters also vary and the study is not aimed at studying the separate effects of adult/child ratio. |
| Confounding | Critical risk of bias | The states have different regulations regarding adult/child ratio, but a lot of other parameters also vary and the study is not aimed at studying the separate effects of adult/child ratio. |
| Selection | Critical risk of bias |  |
| Classification | Serious risk of bias |  |
| Deviation | Serious risk of bias |  |
| Missing data | No information |  |
| Measurement | Moderate risk of bias |  |
| Reporting | Low risk of bias |  |

Neuman, & Kaefer (2013)

| **Bias** | **Authors’ judgment** | **Support for judgment** |
| --- | --- | --- |
| Overall study rating | Moderate risk of bias | Overall this seems like a well-thought out experiment. Single group design, each child receives instructions on sets of words in a whole-group and small group condition and thus serves as his/her own control. |
| Confounding | Low risk of bias | Each child receives instructions on sets of words in a whole-group and small group condition and thus serves as his/her own control. |
| Selection | Low risk of bias | Nothing to suggest selection bias |
| Classification | Low risk of bias | Nothing to suggest classification bias |
| Deviation | Low risk of bias | Nothing to suggest deviation bias |
| Missing data | Moderate risk of bias | 9 % attrition (p. 598). |
| Measurement | Low risk of bias | Reliability is provided for Curriculum-related word knowledge; Conceptual knowledge; Categories and properties knowledge (p.596-597) |
| Reporting | Moderate risk of bias | No reference to a pre-registered analysis plan. Nothing to suggest reporting bias. |

Palmérus (1996)

| **Bias** | **Authors’ judgment** | **Support for judgment** |
| --- | --- | --- |
| Overall study rating | Critical risk of bias | See confounding-domain. |
| Confounding | Critical risk of bias | Single-group design but 4 out of 17 children changed between the two periods, therefore, the treatment effect may be confounded with child effects. Furthermore, children and staff were observed in the low ratio group a year after they were observed in high ratio group. Therefore, a risk of confounding with the age of children. Age ranges of children in the two conditions were "comparable" (p. 48), but the full distribution or means are not shown. No test of differences or balance is performed and no confounder except age is mentioned. Children also spend longer hours in preschool in the second period, which could affect the outcome (p. 50). |
| Selection |  |  |
| Classification |  |  |
| Deviation | Low risk of bias | Group size and ratio are observed. |
| Missing data | No information | Not reported. |
| Measurement | Serious risk of bias | Measures include audiotaped verbal interactions, which are then coded using an unstandardized categorical system, furthermore, no inter-rater reliability is provided. It must therefore be assumed that the coding was done by a single person. |
| Reporting | Moderate risk of bias | There is no a priori analysis plan mentioned, but otherwise no indication of reporting bias. |

### Pelligrino, & Scopesi (1990)

| **Bias** | **Authors’ judgment** | **Support for judgment** |
| --- | --- | --- |
| Overall study rating | Serious risk of bias | It is hard to say what a positive outcome is here, e.g. what is beneficial/better? |
| Confounding | Serious risk of bias | One daycare centre, 5 teachers. There is no information on confounding. |
| Selection | No information | It is not clear how selection of participants was performed. |
| Classification | Low risk of bias | No issues to be reported. Group sizes of 1; 3; 7. |
| Deviation | Low risk of bias | Nothing to suggest deviation bias |
| Missing data | Low risk of bias | As a rule, all teachers interacted with the same fourteen children. Because of irregular attendance, there were a few exceptions, i.e. sometimes one child out of seven was substituted when absent. |
| Measurement | Serious risk of bias | Observers are not blind. It is unclear if the used measures are pre-tested or standardized. Agreement rate is good. |
| Reporting | Serious risk of bias | There is mention of a pre-registered analysis plan. Furthermore, some information is missing. |

Pessanha et al. (2017)

| **Bias** | **Authors’ judgment** | **Support for judgment** |
| --- | --- | --- |
| Overall study rating | Critical risk of bias | There is no manipulation of ratio or group size. This is a correlational design, but well designed. It is unclear why changes in group size and ratio occur over time and why it varies so much between daycare centres (some have small negligible differences over time, while a large number have large differences) and it appears that for all centres group size increases and there are more children per adult. |
| Confounding | Critical risk of bias | Naturally occurring group sizes and ratios. No information on confounding is shown. It is inherently not possible with this design to control for confounding. |
| Selection | Low risk of bias | 90 infant childcare classrooms participated, randomly selected from the 223 institutions registered at the Ministry of Solidarity, Employment and Social Security website. |
| Classification | Low risk of bias | At Time 1, group sizes ranged from 1-12 (M=6.44, SD= 2.38), and the child/adult ratio was on average 2.65 (SD= 1.20), ranging from 0.5 to 7.00. Six months later, at Time 2, group size in each classroom varied between 2 and 18 infants (M= 8.76, SD= 2.54), and the child/adult ratio was on average 3.57 (SD=1.26), ranging from 1.09 to 10. Thus, naturally occurring differences (no manipulation). Group size and ratio observed during data collection. Averages were computed from the data collected both on the observations and in the questionnaires to teachers. |
| Deviation | Low risk of bias | Naturally occurring differences (no manipulation). Group size and ratio were observed during data collection. Averages were computed from the data collected both on the observations and in the questionnaires to teachers. |
| Missing data | Low risk of bias | Seemingly no missing data. |
| Measurement | Moderate risk of bias | The study uses standardized measures: ITERS-R, CLASS-Infant, and CIS. There were trained observers, but outcome assessors were not blind due to observational design. Good inter-rater reliabilities and procedures. |
| Reporting | Moderate risk of bias | There is no pre-registered analysis plan, otherwise no indication of selective reporting. |

Phillips et al. (1992)

| **Bias** | **Authors’ judgment** | **Support for judgment** |
| --- | --- | --- |
| Overall study rating | Critical risk of bias | This quasi-experimental study examines childcare in 5 different cities in the US with different regulations regarding ratio and group size. A number of potential confounders are also studied and found to vary across locations (centre auspice, teacher wages, education, and ongoing professional development). Thus, there are very good reasons to assume that the centres in the different states are not comparable. |
| Confounding | Critical risk of bias | A number of potential confounders are studied and found to vary across locations (centre auspice, teacher wages, education, and ongoing professional development).. |
| Selection | Critical risk of bias | Selection into the intervention was very strongly related to intervention and outcome |
| Classification | Moderate risk of bias | Intervention status is well defined |
| Deviation | Serious risk of bias | Compliance with state regulations varies in centres and across locations, but this is reported and the measure of ratio and group size is the observed one. |
| Missing data | No information |  |
| Measurement | Low risk of bias | Measures are validated such as ECERS etc. |
| Reporting | Moderate risk of bias | There is no mention of a pre-registered analysis plan. Nothing to suggest reporting bias. |

Phillips et al. 2000

| **Bias** | **Authors’ judgment** | **Support for judgment** |
| --- | --- | --- |
| Overall study rating | Critical risk of bias | QES that compares centers in states with different ratio requirements. A representative sample og day care centres in three different states was recruited, the three different locations have different regulation of adult/child ratio and group size (among other things) and thus represent different ratio + group size conditions, however many other confounders also vary by location. |
| Confounding | Critical risk of bias | Teacher wages, parental fees, teacher education are very unequally distributed and there are no reports of SES, minority status etc. of the parents. |
| Selection | Serious risk of bias |  |
| Classification | Serious risk of bias |  |
| Deviation | Serious risk of bias |  |
| Missing data | No information |  |
| Measurement | Low risk of bias |  |
| Reporting | Moderate risk of bias | There is no mention of a pre-registered analysis plan. Nothing to suggest reporting bias, but a lot of information is missing. |

Phillips, & Twardosz (2003)

| **Bias** | **Authors’ judgment** | **Support for judgment** |
| --- | --- | --- |
| Overall study rating | Moderate risk of bias | This is a really interesting study on children's participation during book reading in small and large groups. Authors were contacted but data is no longer available, so an effect size cannot be calculated (SDs are not reported). |
| Confounding | Moderate risk of bias | Only two classrooms are involved and only 15 children, confounders are not really reported. |
| Selection | Low risk of bias | Nothing to suggest selection bias |
| Classification | Moderate risk of bias | The group sizes for small and large groups vary a little from day to day, but over time an average is calculated which seems like a good approach. |
| Deviation | Moderate risk of bias | As the group sizes vary (due to the total number of children being present in the classroom each day varying), the intervention may be seen as less successfully implemented on some days. |
| Missing data | Moderate risk of bias | Not reported, it says that it is a naturalistic experiment and that the children present + the number of children varies every day and thus an average is calculated. |
| Measurement | Moderate risk of bias | The study uses observations/video recordings, which are coded by observers and the authors. This seems appropriate, but some of the measures are perhaps not validated on other samples. |
| Reporting | Moderate risk of bias | No a priori analysis plan is mentioned, otherwise nothing to suggest reporting bias. |

Pierce-Jones, & Jones (1968)

| **Bias** | **Authors’ judgment** | **Support for judgment** |
| --- | --- | --- |
| Overall study rating | Critical risk of bias | The study design is poorly described, measures are not standardized or validated, no confounders are mentioned or reported by group status and the intervention is not clearly defined. The experimental group are taught by mothers of whom no details regarding education and experience are reported,, as opposed to the experimental group who are taught by regular teachers. |
| Confounding | Critical risk of bias | Moderators are not really mentioned or reported by group status, however the experimental group are taught by mothers of whom no details regarding education and experience are reported,, as opposed to the experimental group who are taught by regular teachers. |
| Selection | Serious risk of bias |  |
| Classification | Moderate risk of bias |  |
| Deviation | Serious risk of bias |  |
| Missing data | No information |  |
| Measurement | Critical risk of bias |  |
| Reporting | Moderate risk of bias |  |

Russell (1990)

| **Bias** | **Authors’ judgment** | **Support for judgment** |
| --- | --- | --- |
| Overall study rating | Moderate risk of bias | Overall, this is a very well designed study; however, there is some missing information, which makes it difficult to judge the risk of bias. |
| Confounding | Moderate risk of bias | Single-group design. All children participate in all ratio conditions, therefore, the mean over sessions should be un-confounded by child characteristics, given that the sessions are close in time. It says that the order of observations in each preschool was random (method not specified) (p. 79). |
| Selection | Low risk of bias | All subjects are included in all conditions. There is no information about lack of observations or attrition of subjects. |
| Classification | Low risk of bias | The intervention groups were clearly defined and the information used to define intervention groups was recorded at the start of the intervention. Classification of intervention status was not affected by knowledge of the outcome. |
| Deviation | Low risk of bias | The observed ratio (calculated as an average across observation periods for each of the 3 conditions) differed slightly from the intended, but differences were still meaningful e.g. low ratio was defined as 7.7:1 (instead of 8:1), average ratio was defined as 9.2:1 instead of 10:1, and high ratio was defined as 11.2:1 instead of 12:1. |
| Missing data | Low risk of bias | No missing data reported. |
| Measurement | Serious risk of bias | The outcome measures could have been influenced by knowledge of received intervention and outcome assessors were aware of the intervention received by study participants. The methods of assessment were comparable across intervention groups and there were probably not any systematic errors in measurement of the outcome related to the intervention received. |
| Reporting | Moderate risk of bias | Given that there is no protocol, the authors might have had more outcomes that are not reported in this publication. |

Smith, McMillan, Kennedy, & Ratcliffe (1988)/ Smith et al. (1988)

| **Bias** | **Authors’ judgment** | **Support for judgment** |
| --- | --- | --- |
| Overall study rating | Serious risk of bias | Serious risk of confounding bias and high attrition. |
| Confounding | Serious risk of bias | Participants were probably not able to switch between treatment groups. Kindergartens are matched on location (by city). Not all pre-specified confounders are not reported. Child-teacher interaction and teacher behavior pre-treatment scores are, with possibly one or two exceptions, reasonably well-balanced. The group size is however different at baseline, there were larger groups in the control group. There is no adjustment for confounders in the analysis that we can use. Pre-treatment scores seem to be measured reliably. |
| Selection | Moderate risk of bias | The assignment mechanism is unclear, but not participants are not chosen based on post-test intervention characteristics. Most participants are followed from the start of the intervention, but see the missing data-domain. |
| Classification | Low risk of bias | Some data regarding pre-intervention adult/child ratio is missing, but this does not indicate problems. |
| Deviation | Serious risk of bias | There is no information regarding potential co-interventions. There are implementation problems in that not all preschools get an extra teacher as intended and there are other adult extras in the control group. I.e., the adult-child ratio difference between the treatment and control group is smaller than intended. |
| Missing data | Serious risk of bias | Originally 48 children, 47 children observed at first data collection, 35 participated in all three (attrition 26%). Participants are not purposefully excluded due to missing data on intervention status or other variables needed for the analysis but a larger proportion of control (2 teacher-kindergartens) children leave the sample (41-43% leave, compared to 13% in the treatment group, p. 28). There are no tests of robustness to missing data. |
| Measurement | Serious risk of bias | Outcome assessors could not be blinded to the intervention, which may have affected the measurement. Methods of outcome assessment otherwise comparable across groups. |
| Reporting | Moderate risk of bias | There is no mention of a pre-registered analysis plan. Nothing to suggest reporting bias, but a lot of information is missing. |

Smith, & Connolly (1986)

| **Bias** | **Authors’ judgment** | **Support for judgment** |
| --- | --- | --- |
| Overall study rating | Serious risk of bias | Looks at playgroups (3 hours a couple of days a week), not 5 days a week, full day preschool. This needs awareness in the further analysis. Study lasting 3 years, with manipulations of space and equipment, free play vs. structured activity, and class size and staff-child ratio. The results from the 3rd year are interesting for the analysis. One site, two group replication design with 24 children in each group. Direct observations on children and staff. It is not certain whether it is a problem that there was only 1 sit (with two groups). It is difficult to rate this study due to the lack of information. |
| Confounding | No information | No confounders are shown. It is not clear if/how the authors have performed analyses on confounding. What is manipulated is designed with variation in order to isolate effects. Furthermore, two groups are used to avoid unit effects. |
| Selection | Low risk of bias | It is stated that selection was tightly controlled (in that authors could control who to give a place in one of the two play groups). Therefore, sex and age of children was tightly balanced (non-random selection). |
| Classification | Low risk of bias | Group size and ratio variations are tightly controlled. |
| Deviation | Low risk of bias | Carefully designed manipulation and seemingly no flaws in implementation. |
| Missing data | No information | No information provided. |
| Measurement | Serious risk of bias | Observers are not blind. The used measures are not standardized. Inter-observer agreement is stated to be good. |
| Reporting | Moderate risk of bias | There is no pre-registered analysis plan. There is no indication of selective reporting; however, information is missing. |

Travers et al. (1980) / Smith, & Spence (1980)

| **Bias** | **Authors’ judgment** | **Support for judgment** |
| --- | --- | --- |
| Overall study rating | Moderate risk of bias | The quasi-experiment compared three groups of centers: Group 1 was treatment group, where low ratios were increased (from 1:9,1 to 1:5,9). Effects of this increase were compared with a matched group of 14 untreated low-ratio centers (1:9,1, Group 2), and with a group of 21 untreated high-ratio centers (1:5,9, Group 3). The randomized experiment included 8 centers, 29 classrooms. Random assignment within centers to classrooms that differed systematically in level of staff education and ratio. Ratio and education were crossed in a two-way factorial design. |
| Confounding | Moderate risk of bias | Authors account in detail for confounding (including tests for dependence between variables). Groups seem to have been created in order to achieve reasonable balance. It is not certain whether the lack of group size manipulation is problematic. |
| Selection | Low risk of bias | Random selection and matching in the quasi-experiment. |
| Classification | Low risk of bias | Measurement of ratio seems solid and based on extensive observation and roster/schedule data. |
| Deviation | Low risk of bias | Carefully designed observations and schedule/roster data. |
| Missing data | Low risk of bias | Moderate and almost unrelated to policy variables (Table 2.6). There is no systematic dropout. |
| Measurement | Low risk of bias | Observer effects are considered in detail. Use of standardized measures. |
| Reporting | Moderate risk of bias | It is not certain whether there is a pre-registered analysis plan. No indication of selective reporting biases (very detailed and full report). |

Travers et al. (1980 **)**

| **Bias** | **Authors’ judgment** | **Support for judgment** |
| --- | --- | --- |
| Overall study rating | Moderate risk of bias | The quasi-experiment compared three groups of centers: Group 1 was treatment group, where low ratios were increased (from 1:9,1 to 1:5,9). Effects of this increase were compared with a matched group of 14 untreated low-ratio centers (1:9,1, Group 2), and with a group of 21 untreated high-ratio centers (1:5,9, Group 3). The randomized experiment included 8 centers, 29 classrooms. Random assignment within centers to classrooms that differed systematically in level of staff education and ratio. Ratio and education were crossed in a two-way factorial design. |
| Confounding | Moderate risk of bias | Quasi-experiment: moderate and randomized experiment: low/moderate. Authors account in detail for confounding (including tests for dependence between variables). Groups seem to have been created in order to achieve reasonable balance. It is not certain whether the lack of group size manipulation is problematic. |
| Selection | Low risk of bias | Random selection and matching in the quasi-experiment. |
| Classification | Low risk of bias | Measurement of ratio seems solid and based on extensive observation and roster/schedule data. |
| Deviation | Low risk of bias | Carefully designed observations and schedule/roster data. |
| Missing data | Low risk of bias | Moderate and almost unrelated to policy variables (Table 2.6). There is no systematic dropout. |
| Measurement | Low risk of bias | Observer effects are considered in detail. Use of standardized measures. |
| Reporting | Moderate risk of bias | It is not certain whether there is a pre-registered analysis plan. No indication of selective reporting biases (very detailed and full report). |

## 2 Search Strategy

**Electronic searches**

Socindex

Searched through EBSCO-host. Search performed 23/01/2020.

| **Search** | **Terms** | **Results** |
| --- | --- | --- |
| S13 | S3 AND S8 AND S12 | 190 |
| S12 | S9 OR S10 OR S11 | 12,625 |
| S11 | AB (caretaker* OR teacher* OR staff* OR caregiver* OR adult*) AND AB (ratio*) | 5,311 |
| S10 | AB (“group size*” OR "class size*") | 1,063 |
| S9 | TI (“group size*” OR "class size*" OR ratio*) | 6,692 |
| S8 | S4 OR S5 OR S6 OR S7 | 27,093 |
| S7 | AB (care N2 (center* OR centre* OR day* OR child*)) | 15,194 |
| S6 | TI (care N2 (center* OR centre* OR day* OR child*)) | 4,346 |
| S5 | AB (preschool* OR “non parental” OR kindergarten* OR nurser* OR "early childhood education and care") | 9,786 |
| S4 | TI (preschool* OR “non parental” OR kindergarten* OR nurser* OR "early childhood") | 5,253 |
| S3 | S1 OR S2 | 390,672 |
| S2 | AB (Infant* OR toddler* OR child* OR pupil* OR student* OR newborn* OR neonate* OR baby* OR babies) | 372,387 |
| S1 | TI (Infant* OR toddler* OR child* OR pupil* OR student* OR newborn* OR neonate* OR baby* OR babies) | 147,118 |

*PsycINFO*

Searched through EBSCO-host. Search performed 22/02/2022.

| **Search** | **Terms** | **Results** |
| --- | --- | --- |
| S23 | S7 AND S17 AND S22 | 1,227 |
| S22 | S18 OR S19 OR S20 OR S21 *INTERVENTION* | 43,640 |
| S21 | DE "Class Size" | 339 |
| S20 | AB ( caretaker* OR teacher* OR staff* OR caregiver* OR adult* ) AND AB ratio* | 26,049 |
| S18 | TI "group size*" OR "class size*" OR ratio* | 14,861 |
| S17 | S8 OR S9 OR S10 OR S11 OR S12 OR S13 OR S14 OR S15 OR S16 *SETTING* | 117,910 |
| S16 | ((DE "Child Care" OR DE "Child Day Care") OR (DE "Kindergartens")) OR (DE "Preschool Education") | 18,888 |
| S15 | AB (care N2 (center* OR centre* OR day* OR child*)) | 34,613 |
| S15 | AB (care N2 (center* OR centre* OR day* OR child*)) | 34,613 |
| S14 | TI (care N2 (center* OR centre* OR day* OR child*)) | 9,787 |
| S13 | AB (early N5 education) | 9,191 |
| S12 | TI (early N5 education) | 2,922 |
| S11 | AB "ECE" OR "ECEC" OR "ECCE" OR "creche" OR prekindergarten OR "pre-kindergarten" OR "pre-K" OR "pre K" OR "head start" OR "community based child care" OR "community-based child care" OR "center based child care" OR "center-based child care" OR "family child care" OR "home based child care" OR "home-based child care" | 6,797 |
| S10 | AB preschool* OR "pre-school*" OR "non parental" OR "non-parental" OR kindergarten* OR nurser* OR "early childhood education and care"" | 58,147 |
| S9 | TI "ECE" OR "ECEC" OR "ECCE" OR "creche" OR prekindergarten OR "pre-kindergarten" OR "pre-K" OR "pre K" OR "head start" OR "community based child care" OR "community-based child care" OR "center based child care" OR "center-based child care" OR "family child care" OR "home based child care" OR "home-based child care" | 2,604 |
| S8 | TI preschool* OR "pre-school*" OR "non parental" OR "non-parental" OR kindergarten* OR nurser* OR "early childhood" | 38,254 |
| S7 | S1 OR S2 OR S3 OR S4 OR S5 OR S6 *POPULATION* | 1,305,104 |
| S6 | (ZG "infancy (2-23 mo)") or (ZG "neonatal (birth-1 mo)") or (ZG "preschool age (2-5 yrs)") | 182,871 |
| S5 | DE "Preschool Students" OR DE "Nursery School Students" OR DE "Kindergarten Students" | 18,187 |
| S4 | AB preschooler OR "one-year-old*" OR "one year old*" OR "1 year* old*" OR "two-year-old*" OR "two year old*" OR "2 year* old*" OR "three-year-old*" OR "three year old*" OR "3 year* old*" OR "four-year-old*" OR "four year old*" OR "4 year* old*" OR "five-year-old*" OR "five year old*" OR "5 year* old*" | 16,100 |
| S3 | AB infant* OR toddler* OR child* OR pupil* OR student* OR newborn* OR neonate* OR baby* OR babies | 1,209,143 |
| S2 | TI preschooler OR "one-year-old*" OR "one year old*" OR "1 year* old*" OR "two-year-old*" OR "two year old*" OR "2 year* old*" OR "three-year-old*" OR "three year old*" OR "3 year* old*" OR "four-year-old*" OR "four year old*" OR "4 year* old*" OR "five-year-old*" OR "five year old*" OR "5 year* old*" | 8,083 |
| S1 | TI infant* OR toddler* OR child* OR pupil* OR student* OR newborn* OR neonate* OR baby* OR babies | 566,341 |

*Econlit*

Searched through EBSCO-host. Search performed 23/01/2020.

| **Search** | **Terms** | **Results** |
| --- | --- | --- |
| S13 | S3 AND S8 AND S12 | 65 |
| S12 | S9 OR S10 OR S11 | 15,020 |
| S11 | AB (caretaker* OR teacher* OR staff* OR caregiver* OR adult*) AND AB (ratio*) | 879 |
| S10 | AB (“group size*” OR "class size*") | 816 |
| S9 | TI (“group size*” OR "class size*" OR ratio*) | 13,574 |
| S8 | S4 OR S5 OR S6 OR S7 | 3,202 |
| S7 | AB (care N2 (center* OR centre* OR day* OR child*)) | 1,831 |
| S6 | TI (care N2 (center* OR centre* OR day* OR child*)) | 889 |
| S5 | AB (preschool* OR “non parental” OR kindergarten* OR nurser* OR "early childhood education and care") | 869 |
| S4 | TI (preschool* OR “non parental” OR kindergarten* OR nurser* OR "early childhood") | 602 |
| S3 | S1 OR S2 | 48,886 |
| S2 | AB (Infant* OR toddler* OR child* OR pupil* OR student* OR newborn* OR neonate* OR baby* OR babies) | 43,505 |
| S1 | TI (Infant* OR toddler* OR child* OR pupil* OR student* OR newborn* OR neonate* OR baby* OR babies) | 17,603 |

*ERIC*

Searched through EBSCO-host. Search performed 22/02/2022.

| **Search** | **Terms** | **Results** |
| --- | --- | --- |
| S23 | S6 AND S17 AND S22 | 2,051 |
| S22 | S18 OR S19 OR S20 OR S21 | 23,273 |
| S21 | DE "Class Size" | 3,469 |
| S20 | AB ( caretaker* OR teacher* OR staff* OR caregiver* OR adult* ) AND AB ratio* | 14,298 |
| S19 | AB "group size*" OR "class size*" | 4,985 |
| S18 | TI "group size*" OR "class size*" OR ratio* | 4,334 |
| S17 | S7 OR S8 OR S9 OR S10 OR S11 OR S12 OR S13 OR S14 OR S15 OR S16 | 96,785 |
| S16 | (ZU "child care") or (ZU "child care centers") | 5,875 |
| S15 | AB (care N2 (center* OR centre* OR day* OR child*)) | 18,452 |
| S14 | TI (care N2 (center* OR centre* OR day* OR child*)) | 6,835 |
| S13 | AB (early N5 education) | 16,266 |
| S12 | TI (early N5 education) | 5,877 |
| S11 | (((ZF "preschool education")) or ((ZF "kindergarten"))) or ((ZU "preschool education") or (ZU "preschool learning")) | 38,367 |
| S10 | AB "ECE" OR "ECEC" OR "ECCE" OR "creche" OR prekindergarten OR "pre-kindergarten" OR "pre-K" OR "pre K" OR "head start" OR "community based child care" OR "community-based child care" OR "center based child care" OR "center-based child care" OR "family child care" OR "home based child care" OR "home-based child care" | 10,304 |
| S9 | AB preschool* OR "pre-school*" OR "non parental" OR "non-parental" OR kindergarten* OR nurser* OR "early childhood education and care" | 50,191 |
| S8 | TI "ECE" OR "ECEC" OR "ECCE" OR "creche" OR prekindergarten OR "pre-kindergarten" OR "pre-K" OR "pre K" OR "head start" OR "community based child care" OR "community-based child care" OR "center based child care" OR "center-based child care" OR "family child care" OR "home based child care" OR "home-based child care" | 3,802 |
| S7 | TI preschool* OR "pre-school*" OR "non parental" OR "non-parental" OR kindergarten* OR nurser* OR "early childhood" | 30,346 |
| S6 | S1 OR S2 OR S3 OR S4 OR S5 | 997,504 |
| S5 | DE "Preschool Children" OR DE "Kindergarten" OR DE "Young Children" OR DE "Infants" OR DE "Preschool Children" OR DE "Toddlers" | 72,329 |
| S4 | AB preschooler OR "one-year-old*" OR "one year old*" OR "1 year* old*" OR "two-year-old*" OR "two year old*" OR "2 year* old*" OR "three-year-old*" OR "three year old*" OR "3 year* old*" OR "four-year-old*" OR "four year old*" OR "4 year* old*" OR "five-year-old*" OR "five year old*" OR "5 year* old*" | 9,502 |
| S3 | AB infant* OR toddler* OR child* OR pupil* OR student* OR newborn* OR neonate* OR baby* OR babies | 966,923 |
| S2 | TI preschooler OR "one-year-old*" OR "one year old*" OR "1 year* old*" OR "two-year-old*" OR "two year old*" OR "2 year* old*" OR "three-year-old*" OR "three year old*" OR "3 year* old*" OR "four-year-old*" OR "four year old*" OR "4 year* old*" OR "five-year-old*" OR "five year old*" OR "5 year* old*" | 4,055 |
| S1 | TI infant* OR toddler* OR child* OR pupil* OR student* OR newborn* OR neonate* OR baby* OR babies | 343,347 |

*Teacher Reference Center*

Searched through EBSCO-host. Search performed 23/01/2020.

| **Search** | **Terms** | **Results** |
| --- | --- | --- |
| S13 | S3 AND S8 AND S12 | 104 |
| S12 | S9 OR S10 OR S11 | 3,636 |
| S11 | AB (caretaker* OR teacher* OR staff* OR caregiver* OR adult*) AND AB (ratio*) | 1,559 |
| S10 | AB (“group size*” OR "class size*") | 1,385 |
| S9 | TI (“group size*” OR "class size*" OR ratio*) | 1,115 |
| S8 | S4 OR S5 OR S6 OR S7 | 20,342 |
| S7 | AB (care N2 (center* OR centre* OR day* OR child*)) | 1,800 |
| S6 | TI (care N2 (center* OR centre* OR day* OR child*)) | 491 |
| S5 | AB (preschool* OR “non parental” OR kindergarten* OR nurser* OR "early childhood education and care") | 7,710 |
| S4 | TI (preschool* OR “non parental” OR kindergarten* OR nurser* OR "early childhood") | 13,647 |
| S3 | S1 OR S2 | 309,033 |
| S2 | AB (Infant* OR toddler* OR child* OR pupil* OR student* OR newborn* OR neonate* OR baby* OR babies) | 298,927 |
| S1 | TI (Infant* OR toddler* OR child* OR pupil* OR student* OR newborn* OR neonate* OR baby* OR babies) | 72,556 |

*Academic Search Premier*

Searched through EBSCO-host. Search performed 23/01/2020.

| **Search** | **Terms** | **Results** |
| --- | --- | --- |
| S13 | S3 AND S8 AND S12 | 981 |
| S12 | S9 OR S10 OR S11 | 142,958 |
| S11 | AB (caretaker* OR teacher* OR staff* OR caregiver* OR adult*) AND AB (ratio*) | 57,044 |
| S10 | AB (“group size*” OR "class size*") | 6,936 |
| S9 | TI (“group size*” OR "class size*" OR ratio*) | 82,554 |
| S8 | S4 OR S5 OR S6 OR S7 | 133,706 |
| S7 | AB (care N2 (center* OR centre* OR day* OR child*)) | 59,156 |
| S6 | TI (care N2 (center* OR centre* OR day* OR child*)) | 15,642 |
| S5 | AB (preschool* OR “non parental” OR kindergarten* OR nurser* OR "early childhood education and care") | 51,259 |
| S4 | TI (preschool* OR “non parental” OR kindergarten* OR nurser* OR "early childhood") | 37,465 |
| S3 | S1 OR S2 | 2,015,288 |
| S2 | AB (Infant* OR toddler* OR child* OR pupil* OR student* OR newborn* OR neonate* OR baby* OR babies) | 1,887,931 |
| S1 | TI (Infant* OR toddler* OR child* OR pupil* OR student* OR newborn* OR neonate* OR baby* OR babies) | 719,933 |

*Science Citation Index*

Searched through Web of Science. Search performed 24/01/2020.

| **Search** | **Results** | **Terms** |
| --- | --- | --- |
| # 13 | 161 | #3 AND #8 AND #12  *Indexes=SCI-EXPANDED Timespan=All years* |
| # 12 | 200,268 | #9 OR #10 OR #11  *Indexes=SCI-EXPANDED Timespan=All years* |
| # 11 | 131 | AB=((caretaker* OR teacher* OR staff* OR caregiver* OR adult*) AND AB ratio*)  *Indexes=SCI-EXPANDED Timespan=All years* |
| # 10 | 6,627 | AB=(“group size*” OR "class size*")  *Indexes=SCI-EXPANDED Timespan=All years* |
| # 9 | 194,473 | TI=(“group size*” OR "class size*" OR ratio*)  *Indexes=SCI-EXPANDED Timespan=All years* |
| # 8 | 110,337 | #4 OR #5 OR #6 OR #7  *Indexes=SCI-EXPANDED Timespan=All years* |
| # 7 | 50,910 | AB=(care NEAR/2 (center* OR centre* OR day* OR child*))  *Indexes=SCI-EXPANDED Timespan=All years* |
| # 6 | 18,889 | TI=(care NEAR/2 (center* OR centre* OR day* OR child*))  *Indexes=SCI-EXPANDED Timespan=All years* |
| # 5 | 31,213 | AB=(preschool* OR “non parental” OR kindergarten* OR nurser* OR "early childhood education and care")  *Indexes=SCI-EXPANDED Timespan=All years* |
| # 4 | 25,321 | TI=(preschool* OR “non parental” OR kindergarten* OR nurser* OR "early childhood")  *Indexes=SCI-EXPANDED Timespan=All years* |
| # 3 | 1,624,654 | #1 OR #2  *Indexes=SCI-EXPANDED Timespan=All years* |
| # 2 | 1,058,745 | AB=(Infant* OR toddler* OR child* OR pupil* OR student* OR newborn* OR neonate* OR baby* OR babies)  *Indexes=SCI-EXPANDED Timespan=All years* |
| # 1 | 988,059 | TI=(Infant* OR toddler* OR child* OR pupil* OR student* OR newborn* OR neonate* OR baby* OR babies)  *Indexes=SCI-EXPANDED Timespan=All years* |

*Social Science Citation Index*

Searched through Web of Science. Search performed 24/01/2020.

| **Search** | **Results** | **Terms** |
| --- | --- | --- |
| # 13 | 248 | #3 AND #8 AND #12  *Indexes=SSCI Timespan=All years* |
| # 12 | 31,819 | #9 OR #10 OR #11  *Indexes=SSCI Timespan=All years* |
| # 11 | [18](http://apps.webofknowledge.com/summary.do?product=WOS&doc=1&qid=45&SID=C1sy8RueJa8586dT164&search_mode=AdvancedSearch&update_back2search_link_param=yes) | AB=((caretaker* OR teacher* OR staff* OR caregiver* OR adult*) AND AB ratio*)  *Indexes=SSCI Timespan=All years* |
| # 10 | [2,768](http://apps.webofknowledge.com/summary.do?product=WOS&doc=1&qid=42&SID=C1sy8RueJa8586dT164&search_mode=AdvancedSearch&update_back2search_link_param=yes) | AB=(“group size*” OR "class size*")  *Indexes=SSCI Timespan=All years* |
| # 9 | [29,521](http://apps.webofknowledge.com/summary.do?product=WOS&doc=1&qid=39&SID=C1sy8RueJa8586dT164&search_mode=AdvancedSearch&update_back2search_link_param=yes) | TI=(“group size*” OR "class size*" OR ratio*)  *Indexes=SSCI Timespan=All years* |
| # 8 | [75,984](http://apps.webofknowledge.com/summary.do?product=WOS&doc=1&qid=37&SID=C1sy8RueJa8586dT164&search_mode=AdvancedSearch&update_back2search_link_param=yes) | #4 OR #5 OR #6 OR #7  *Indexes=SSCI Timespan=All years* |
| # 7 | [28,151](http://apps.webofknowledge.com/summary.do?product=WOS&doc=1&qid=34&SID=C1sy8RueJa8586dT164&search_mode=AdvancedSearch&update_back2search_link_param=yes) | AB=(care NEAR/2 (center* OR centre* OR day* OR child*))  *Indexes=SSCI Timespan=All years* |
| # 6 | [13,799](http://apps.webofknowledge.com/summary.do?product=WOS&doc=1&qid=31&SID=C1sy8RueJa8586dT164&search_mode=AdvancedSearch&update_back2search_link_param=yes) | TI=(care NEAR/2 (center* OR centre* OR day* OR child*))  *Indexes=SSCI Timespan=All years* |
| # 5 | [25,394](http://apps.webofknowledge.com/summary.do?product=WOS&doc=1&qid=28&SID=C1sy8RueJa8586dT164&search_mode=AdvancedSearch&update_back2search_link_param=yes) | AB=(preschool* OR “non parental” OR kindergarten* OR nurser* OR "early childhood education and care")  *Indexes=SSCI Timespan=All years* |
| # 4 | [27,525](http://apps.webofknowledge.com/summary.do?product=WOS&doc=1&qid=25&SID=C1sy8RueJa8586dT164&search_mode=AdvancedSearch&update_back2search_link_param=yes) | TI=(preschool* OR “non parental” OR kindergarten* OR nurser* OR "early childhood")  *Indexes=SSCI Timespan=All years* |
| # 3 | [876,512](http://apps.webofknowledge.com/summary.do?product=WOS&doc=1&qid=23&SID=C1sy8RueJa8586dT164&search_mode=AdvancedSearch&update_back2search_link_param=yes) | #1 OR #2  *Indexes=SSCI Timespan=All years* |
| # 2 | [619,098](http://apps.webofknowledge.com/summary.do?product=WOS&doc=1&qid=20&SID=C1sy8RueJa8586dT164&search_mode=AdvancedSearch&update_back2search_link_param=yes) | AB=(Infant* OR toddler* OR child* OR pupil* OR student* OR newborn* OR neonate* OR baby* OR babies)  *Indexes=SSCI Timespan=All years* |
| # 1 | [525,739](http://apps.webofknowledge.com/summary.do?product=WOS&doc=1&qid=17&SID=C1sy8RueJa8586dT164&search_mode=AdvancedSearch&update_back2search_link_param=yes) | TI=(Infant* OR toddler* OR child* OR pupil* OR student* OR newborn* OR neonate* OR baby* OR babies)  *Indexes=SSCI Timespan=All years* |

*Sociological Abstracts*

Searched through ProQuest-host. Search performed 24/01/2020.

| **Search** | **Terms** | **Results** |
| --- | --- | --- |
| S14 | [S3 AND S9 AND S13](https://search.proquest.com/recentsearches.recentsearchtabview.recentsearchesgridview.scrolledrecentsearchlist.checkdbssearchlink:rerunsearch/EE41AD0CC4F44713PQ/None?site=socabs&t:ac=RecentSearches) | [38](https://search.proquest.com/recentsearches.recentsearchtabview.recentsearchesgridview.scrolledrecentsearchlist.checkdbssearchlink_0:rerunsearch/EE41AD0CC4F44713PQ/None?site=socabs&t:ac=RecentSearches) |
| S13 | [S10 OR S11 OR S12](https://search.proquest.com/recentsearches.recentsearchtabview.recentsearchesgridview.scrolledrecentsearchlist.checkdbssearchlink:rerunsearch/878587C04C034C7CPQ/None?site=socabs&t:ac=RecentSearches) | [4,665](https://search.proquest.com/recentsearches.recentsearchtabview.recentsearchesgridview.scrolledrecentsearchlist.checkdbssearchlink_0:rerunsearch/878587C04C034C7CPQ/None?site=socabs&t:ac=RecentSearches) |
| S12 | [ab(“group size*” OR "class size*")](https://search.proquest.com/recentsearches.recentsearchtabview.recentsearchesgridview.scrolledrecentsearchlist.checkdbssearchlink:rerunsearch/5E8DC4CCA3A74C05PQ/None?site=socabs&t:ac=RecentSearches) | [857](https://search.proquest.com/recentsearches.recentsearchtabview.recentsearchesgridview.scrolledrecentsearchlist.checkdbssearchlink_0:rerunsearch/5E8DC4CCA3A74C05PQ/None?site=socabs&t:ac=RecentSearches) |
| S11 | [ab(caretaker* OR teacher* OR staff* OR caregiver* OR adult*) AND AB (ratio*](https://search.proquest.com/recentsearches.recentsearchtabview.recentsearchesgridview.scrolledrecentsearchlist.checkdbssearchlink:rerunsearch/E464D2B9B0E34F6BPQ/None?site=socabs&t:ac=RecentSearches)) | [244](https://search.proquest.com/recentsearches.recentsearchtabview.recentsearchesgridview.scrolledrecentsearchlist.checkdbssearchlink_0:rerunsearch/E464D2B9B0E34F6BPQ/None?site=socabs&t:ac=RecentSearches) |
| S10 | [TI(“group size*” OR "class size*" OR ratio*)](https://search.proquest.com/recentsearches.recentsearchtabview.recentsearchesgridview.scrolledrecentsearchlist.checkdbssearchlink:rerunsearch/8A3F4D11DB2E4724PQ/None?site=socabs&t:ac=RecentSearches) | [3,710](https://search.proquest.com/recentsearches.recentsearchtabview.recentsearchesgridview.scrolledrecentsearchlist.checkdbssearchlink_0:rerunsearch/8A3F4D11DB2E4724PQ/None?site=socabs&t:ac=RecentSearches) |
| S9 | [S4 OR S5 OR S7 OR S8](https://search.proquest.com/recentsearches.recentsearchtabview.recentsearchesgridview.scrolledrecentsearchlist.checkdbssearchlink:rerunsearch/ECA6A6488D584F8APQ/None?site=socabs&t:ac=RecentSearches) | [14,036](https://search.proquest.com/recentsearches.recentsearchtabview.recentsearchesgridview.scrolledrecentsearchlist.checkdbssearchlink_0:rerunsearch/ECA6A6488D584F8APQ/None?site=socabs&t:ac=RecentSearches) |
| S8 | [ab(care NEAR/2 (center* OR centre* OR day* OR child*))](https://search.proquest.com/recentsearches.recentsearchtabview.recentsearchesgridview.scrolledrecentsearchlist.checkdbssearchlink:rerunsearch/7CAD1465AD0346CEPQ/None?site=socabs&t:ac=RecentSearches) | [8,654](https://search.proquest.com/recentsearches.recentsearchtabview.recentsearchesgridview.scrolledrecentsearchlist.checkdbssearchlink_0:rerunsearch/7CAD1465AD0346CEPQ/None?site=socabs&t:ac=RecentSearches) |
| S7 | [ti(care NEAR/2 (center* OR centre* OR day* OR child*))](https://search.proquest.com/recentsearches.recentsearchtabview.recentsearchesgridview.scrolledrecentsearchlist.checkdbssearchlink:rerunsearch/52B7C11E88D54E91PQ/None?site=socabs&t:ac=RecentSearches) | [2,439](https://search.proquest.com/recentsearches.recentsearchtabview.recentsearchesgridview.scrolledrecentsearchlist.checkdbssearchlink_0:rerunsearch/52B7C11E88D54E91PQ/None?site=socabs&t:ac=RecentSearches) |
| S5 | [ab(preschool* OR “non parental” OR kindergarten* OR nurser* OR "early childhood education and care")](https://search.proquest.com/recentsearches.recentsearchtabview.recentsearchesgridview.scrolledrecentsearchlist.checkdbssearchlink:rerunsearch/72558D224BF04994PQ/None?site=socabs&t:ac=RecentSearches) | [4,313](https://search.proquest.com/recentsearches.recentsearchtabview.recentsearchesgridview.scrolledrecentsearchlist.checkdbssearchlink_0:rerunsearch/72558D224BF04994PQ/None?site=socabs&t:ac=RecentSearches) |
| S4 | [ti(preschool* OR “non parental” OR kindergarten* OR nurser* OR "early childhood")](https://search.proquest.com/recentsearches.recentsearchtabview.recentsearchesgridview.scrolledrecentsearchlist.checkdbssearchlink:rerunsearch/B97F376D1FFC456DPQ/None?site=socabs&t:ac=RecentSearches) | [2,262](https://search.proquest.com/recentsearches.recentsearchtabview.recentsearchesgridview.scrolledrecentsearchlist.checkdbssearchlink_0:rerunsearch/B97F376D1FFC456DPQ/None?site=socabs&t:ac=RecentSearches) |
| S3 | [ti((Infant* OR toddler* OR child* OR pupil* OR student* OR newborn* OR neonate* OR baby* OR babies)) OR ab((Infant* OR toddler* OR child* OR pupil* OR student* OR newborn* OR neonate* OR baby* OR babies) )](https://search.proquest.com/recentsearches.recentsearchtabview.recentsearchesgridview.scrolledrecentsearchlist.checkdbssearchlink:rerunsearch/E3B05FACBE9D4FCCPQ/None?site=socabs&t:ac=RecentSearches) | [224,244](https://search.proquest.com/recentsearches.recentsearchtabview.recentsearchesgridview.scrolledrecentsearchlist.checkdbssearchlink_0:rerunsearch/E3B05FACBE9D4FCCPQ/None?site=socabs&t:ac=RecentSearches) |
| S2 | [ab((Infant* OR toddler* OR child* OR pupil* OR student* OR newborn* OR neonate* OR baby* OR babies) )](https://search.proquest.com/recentsearches.recentsearchtabview.recentsearchesgridview.scrolledrecentsearchlist.checkdbssearchlink:rerunsearch/9FAE863B22954947PQ/None?site=socabs&t:ac=RecentSearches) | [209,292](https://search.proquest.com/recentsearches.recentsearchtabview.recentsearchesgridview.scrolledrecentsearchlist.checkdbssearchlink_0:rerunsearch/9FAE863B22954947PQ/None?site=socabs&t:ac=RecentSearches) |
| S1 | [ti((Infant* OR toddler* OR child* OR pupil* OR student* OR newborn* OR neonate* OR baby* OR babies))](https://search.proquest.com/recentsearches.recentsearchtabview.recentsearchesgridview.scrolledrecentsearchlist.checkdbssearchlink:rerunsearch/EA99C14E5CE3471APQ/None?site=socabs&t:ac=RecentSearches) | [80,217](https://search.proquest.com/recentsearches.recentsearchtabview.recentsearchesgridview.scrolledrecentsearchlist.checkdbssearchlink_0:rerunsearch/EA99C14E5CE3471APQ/None?site=socabs&t:ac=RecentSearches) |

**Searching other resources**

*ProQuest Dissertations & Theses Global*

Searched through the ProQuest search platform. Searches were peformed in the advanced search interface. Searches were limited to title and abstract. We used a combination of terms covering the population and the intervention:

Adult student ratio, adult infant ratio, adult toddler ratio, adult pupil ratio, staff child ratio, group size childhood, group size preschool, group size kindergarten, group size parental, teacher student ratio, teacher infant ratio, teacher toddler ratio, teacher child ratio, teacher pupil ratio, staff infant ratio, staff pupil ratio, staff student ratio and staff toddler ratio.

The searches were performed in September 2020 and yielded 257 results.

*EBSCO Open Dissertations*

<http://web.b.ebscohost.com/ehost/search/advanced?vid=0&sid=8447e1d5-bf3b-4126-9a0d-78349468ab56%40sessionmgr103>

Searched through the ProQuest search platform. Searches were peformed in the advanced search interface. Searches were limited to title and abstract. We used a combination of terms covering the population and the intervention:

Adult student ratio, adult infant ratio, adult toddler ratio, adult pupil ratio, staff child ratio, group size childhood, group size preschool, group size kindergarten, group size parental, teacher student ratio, teacher infant ratio, teacher toddler ratio, teacher child ratio, teacher pupil ratio, staff infant ratio, staff pupil ratio, staff student ratio and staff toddler ratio.

The searches were performed in September 2020 and yielded 34 results.

*Open Grey*

<http://www.opengrey.eu/search/>

Searches were performed in the main search field. We used a combination of terms covering the population and the intervention:

Adult student ratio, adult infant ratio, adult toddler ratio, adult pupil ratio, staff child ratio, group size childhood, group size preschool, group size kindergarten, group size parental, teacher student ratio, teacher infant ratio, teacher toddler ratio, teacher child ratio, teacher pupil ratio, staff infant ratio, staff pupil ratio, staff student ratio and staff toddler ratio.

The searches were performed in September 2020 and yielded 29 results.

*Google Scholar*

<https://scholar.google.com/>

Searches were performed in the main search field. We used a combination of terms covering the population and the intervention:

Adult student ratio, adult infant ratio, adult toddler ratio, adult pupil ratio, staff child ratio, group size childhood, group size preschool, group size kindergarten, group size parental, teacher student ratio, teacher infant ratio, teacher toddler ratio, teacher child ratio, teacher pupil ratio, staff infant ratio, staff pupil ratio, staff student ratio and staff toddler ratio.

We also searched Google Scholar using danish terms for the population and intervention:

normering daginstituitoner, gruppestørrelse daginstitutioner, gruppestørelse børnehave

The searches were performed in September 2020 and yielded 1264 results.

*Google searches*

<https://www.google.com/>

Searches were performed in the main search field. We used a combination of terms covering the population and the intervention:

"Adult student ratio", "adult infant ratio", "adult toddler ratio", "adult pupil ratio", "staff child ratio", "group size childhood", "group size preschool", "group size kindergarten", "group size parental", "teacher student ratio"," teacher infant ratio"," teacher toddler ratio", teacher child ratio", "teacher pupil ratio", "staff infant ratio", "staff pupil ratio", "staff student ratio and staff toddler ratio".

The searches were performed in September 2020 and 323 results were screened.

*Evidence Base*

<https://dpu.au.dk/forskning/publikationer/>

This resource has not been updated since 2016. Searches were performed in the Advanced Search search field in the “Publikationer” section. We used a combination of terms covering the population and the intervention:

Adult student ratio, adult infant ratio, adult toddler ratio, adult pupil ratio, staff child ratio, group size childhood, group size preschool, group size kindergarten, group size parental, teacher student ratio, teacher infant ratio, teacher toddler ratio, teacher child ratio, teacher pupil ratio, staff infant ratio, staff pupil ratio, staff student ratio and staff toddler ratio.

The searches were performed in September 2020 and yielded 258 results.

*Campbell Library*

<https://campbellcollaboration.org/better-evidence>

Searches were performed in the Advanced Search interface, limited to title. Searches were limited to non-peer-reviewed references. We searched using a combination of the follow terms:

Adult AND child AND ratio, Adult AND child AND ratio AND education, Adult AND child AND ratio AND achievement, Teacher AND child AND ratio, Group size AND kindergarten, Adult AND child AND ratio AND preschool, Adult AND child AND ratio AND day care, Staff AND child AND ratio

The searches were performed in September 2020 and yielded 13 results.

*Cochrane Library*

<https://www.cochranelibrary.com/>

Searches were performed in the advanced search interface. The searches were limited by using the filter “Developmental, psychosocial & learning problems” and excluding peer-reviewed articles, as well as searches without any limitations. We searched using a combination of the follow terms: adult, child, ratio. The strategy  of using a filter was chosen to limit the search to a managable number of hits, and after  the initial searches without a filter were deemed to be fruitless.

The searches were performed in September 2020 and yielded 758 results.

*Centre for Reviews and Dissemination Databases*

<https://www.crd.york.ac.uk/CRDWeb/>

Searches were limited to the NHS EED and the HTA bases. Searches were performed in “Any Fields”. We used a combination of terms covering the population and the intervention:

Adult AND child AND ratio, Adult AND child AND ratio AND education, Adult AND child AND ratio AND achievement, Teacher AND child AND ratio, Group size AND kindergarten, Adult AND child AND ratio AND preschool, Adult AND child AND ratio AND day care, Staff AND child AND ratio

The searches were performed in September 2020 and yielded 365 results.

*EPPI‐Centre Systematic Reviews – Database of Education Research*

<https://eppi.ioe.ac.uk/webdatabases/Intro.aspx?ID=6>

Searches were performed in the Freetext search interface. No limiters were implemented in the search. We used a combination of terms covering the population and the intervention:

Adult AND child AND ratio, Adult AND pupil AND ratio, Adult AND student AND ratio, Adult AND infant AND ratio, Group size AND non-parental, Group size AND day care, Group size AND Center care, Group size AND preschool, Group size AND childhood, Teacher AND infant AND ratio, Teacher AND student AND ratio, Teacher AND toddler AND ratio, Teacher AND child AND ratio, Teacher AND pupil AND ratio, Staff AND child AND ratio, Staff AND infant AND ratio, Staff AND toddler AND ratio, Staff AND pupil AND ratio, Staff AND student AND ratio, Caretaker AND child AND ratio, Caretaker AND infant AND ratio, caretaker AND pupil AND ratio, Caretaker AND toddler AND ratio

The searches were performed in September 2020 and yielded 41 results.

*Social Care Online*

<https://www.scie-socialcareonline.org.uk/User/login?ReturnUrl=%2fsearch%2fexpert>

Searches were performed in the standard search field. We used a combination of terms covering the population and the intervention:

Adult student ratio, adult infant ratio, adult toddler ratio, adult pupil ratio, staff child ratio, group size childhood, group size preschool, group size kindergarten, group size parental, teacher student ratio, teacher infant ratio, teacher toddler ratio, teacher child ratio, teacher pupil ratio, staff infant ratio, staff pupil ratio, staff student ratio and staff toddler ratio.

The searches were performed in September 2020 and yielded 93 results.

*Social Science Research Network*

<https://www.ssrn.com/index.cfm/en/>

Searches were performed in the standard search field. We used a combination of terms covering the population and the intervention:

Adult student ratio, adult infant ratio, adult toddler ratio, adult pupil ratio, staff child ratio, group size childhood, group size preschool, group size kindergarten, group size parental, teacher student ratio, teacher infant ratio, teacher toddler ratio, teacher child ratio, teacher pupil ratio, staff infant ratio, staff pupil ratio, staff student ratio and staff toddler ratio.

The searches were performed in September 2020 and yielded 150 results.

**European Educational Research Association (EERA)—https://eeraecer.de/**

Searches were performed in the standard search for conference papers field.

Adult student ratio, adult infant ratio, adult toddler ratio, adult pupil ratio, staff child ratio, group size childhood, group size preschool, group size kindergarten, group size parental, teacher student ratio, teacher infant ratio, teacher toddler ratio, teacher child ratio, teacher pupil ratio, staff infant ratio, staff pupil ratio, staff student ratio and staff toddler ratio.

Searches were performed i february 2022 and yielded 20 results

**Nordic Council of Ministers - https://www.norden.org/en/nordic-council-ministers**

we  searched for publications using the filter: " education and research". We used a combination of terms covering the population and the intervention:

| Normering børn |
| --- |
| ratio |
| ratio børn |
| bemandingsnorm |
| bemandingsnorm børn |
| gruppestørrelse dagtilbud |
| gruppestørrelse |
| daginstitution |
| børnehave |
| vuggestue |
| dagtilbud |
| børn |
| ECEC |
| Group size children |
| teacher child ratio |

Searches were performed i february 2022 and yielded  71 results

**NB-ECEC – Scandinavian research in early childhood education and care** :

We used advanced search and used the following filters:

Barn, Børnehave, Børnehaveklasse, Vuggestue, Pædagig, Dagpleje, Dagpleje. Emnekombination:  Kompetencer

Barn, Børnehave, Børnehaveklasse, Vuggestue, Pædagig, Dagpleje, Dagpleje. Emnekombination:  Trivsel

Ledelse og organisering i dagtilbud: Gruppestørrelse

Searches were performed i february 2022 and yielded  245 results

**OECD iLibrary—https://www.oecd-ilibrary.org/**

Advanced Search, Search terms were:

| Adult AND child AND ratio. |
| --- |
| "adult child ratio" |
| "child-staff ratio" |
| "teacher toddler ratio" |
| Teacher AND toddler AND ratio |
| Teacher AND Student AND ratio AND kindergarten |
| "Teacher student ratio" |
| "Teacher student ratio" AND preschool |
| "group size" AND preschool |
| "group size" AND kindergarten |

Searches were performed i february 2022 and yielded  593 result

**U.S. Department of Education -**<https://www.ed.gov/>

We used the search form for individual studies: https://ies.ed.gov/ncee/wwc/ReviewedStudies#/OnlyStudiesWithPositiveEffects:false%7CSetNumber:1%7CEssaRatingId:0,1,2,3

We used the filters: All ratings,Topic:  Early Childhood (pre-K), Design: Randomised controlled trials and Quasi-experimental design, and Evidence rating (all options selected)

Searches were performed i february 2022 and yielded  147 result

**Eurydice Network- https://eacea.ec.europa.eu/national-policies/eurydice/**

We used "simple search" and the search terms used were:

| child-staff ratio |
| --- |
| group size kindergarten |
| group size preschool |
| class size preschool |

Searches were performed i february 2022 and yielded  347 result (including duplicates)

**Citation tracking:**

References lists of previous systematic reviews as well as all included primary studies were screened for relevant new studies to include by at least one review author.
